# Supplementary material for: Evolutionary origins and interactomes of human, young microproteins and small peptides translated from short open reading frames
Source: Mol Cell. 2023 Mar 16;83(6):994–1011.e18. doi: 10.1016/j.molcel.2023.01.023 (PMC10032668; doi:10.1016/j.molcel.2023.01.023)
Supplement: Document S1. Figures S1–S6, Data S1, and supplemental references [file mmc1.pdf]

Molecular Cell, Volume *83*

Supplemental information

Evolutionary origins and interactomes of human, young microproteins and small peptides translated from short open reading frames

Clara-L. Sandmann, Jana F. Schulz, Jorge Ruiz-Orera, Marieluise Kirchner, Matthias Ziehm, Eleonora Adami, Maike Marczenke, Annabel Christ, Nina Liebe, Johannes Greiner, Aaron Schoenenberger, Michael B. Muecke, Ning Liang, Robert L. Moritz, Zhi Sun, Eric W. Deutsch, Michael Gotthardt, Jonathan M. Mudge, John R. Prensner, Thomas E. Willnow, Philipp Mertins, Sebastiaan van Heesch, and Norbert Hubner

Supplemental Figures and Legends

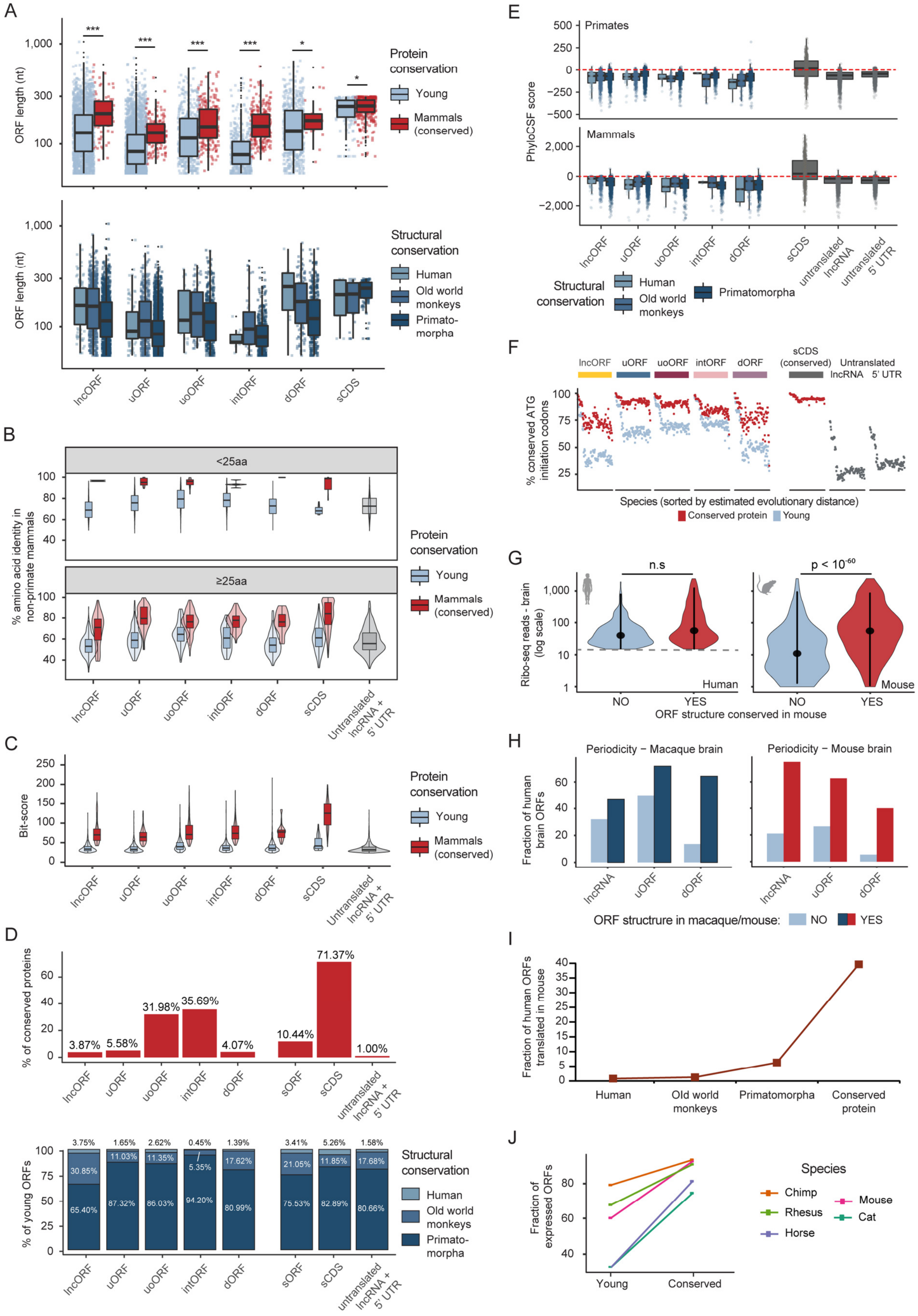

Figure S1. The majority of human sORFs are young and have emerged *de novo*, Related to Figure 1.

(A) Boxplots with length of sORFs (n = 7,264) and sCDS (n = 527) divided by ORF biotype and conservation of amino acid (aa) sequences (top) or ORF structures (bottom, only young ORFs (CS < 8)). Significance of difference was assessed by Wilcoxon test (*, $p < 0.05$; ***, $p < 10^{-5}$).

(B) Boxplots with percentages of average aa identity across non-primate mammals for sORF- and sCDS-encoded microproteins as well as untranslated controls (n = 4,982) divided by ORF biotype, length, and conservation of aa sequences.

(C) Boxplots with ORF alignment bit-scores calculated with BLASTP across the counterpart regions of non-primate mammals for 7,264 sORF- and 527 sCDS-encoded microproteins, as well as 4,982 untranslated RNA regions. ORFs are divided by biotype and conservation of aa sequences.

(D) (Top) Bar plots with the percentages of sORFs, sCDS and untranslated controls whose amino acid sequences are significantly conserved (CS ≥ 8). (Bottom) Bar plots with the percentages of young sORFs (n = 6,506), young sCDSs (n = 148 and untranslated controls (n = 4,982) by evolutionary age based on the conservation of ORF structures. Conserved proteins (CS ≥ 8) are not included.

(E) Boxplots with PhyloCSF scores calculated across all aligned primate (top) and mammalian (bottom) genomes, for young sORFs, sCDSs and untranslated controls. sORFs are divided by ORF biotype and conservation of ORF structures. The distribution of PhyloCSF scores in sORFs is not statistically higher than in untranslated regions (Wilcoxon one-sided signed rank test $\text{p-value}_{\text{primates}} = 1$, $\text{p-value}_{\text{mammals}} = 1$)

(F) Dot plots displaying the percentage of conserved ATG initiation codons in the non-human counterpart sequences of sORF, sCDS, and untranslated ORFs. ORFs are divided by biotype and conservation of aa sequences. ATG initiation codons from young sORFs are significantly more conserved than the ones from untranslated lncRNA and 5' UTR regions (Wilcoxon signed rank test, P-value = 1.45×10^{-21}).

(G) Violin plots with the numbers of mapped Ribo-seq reads (log-scale) of sORFs translated in the human brain (n = 830), by presence (light blue) or absence (red) of conservation in mouse. Mapped reads correspond to human brain (left) or mouse brain (right). Ribo-seq samples had three biological replicates each[1]. Statistical differences in the number of Ribo-seq reads were assessed by Wilcoxon signed rank test. Dot and vertical bar represent the median and the distribution of minimum to maximum values, limited to the most extreme data point that is no more than 1.5x interquantile range (IQR), respectively.

(H) Bar plots with the percentages of human brain translated sORFs with aligned counterpart regions displaying significant Ribo-seq periodicity biases in macaque (left) and mouse (right) brain. sORFs are divided by biotype and by presence (light blue) or absence (dark blue, red) of conservation in macaque or mouse. Upstream overlapping ORFs (uoORFs) and internal ORFs (intORFs) were not considered since the periodicity signal could be masked by translated coding sequences in alternative frames. Significance of periodicity bias was assessed by Binomial test ($p < 0.01$).

(I) Fraction of human sORFs translated in public mouse datasets[1,2] by estimated evolutionary age category.

(J) Fraction of young and conserved human sORFs expressed in chimpanzee, rhesus macaque, horse, mouse, and cat transcriptomes.

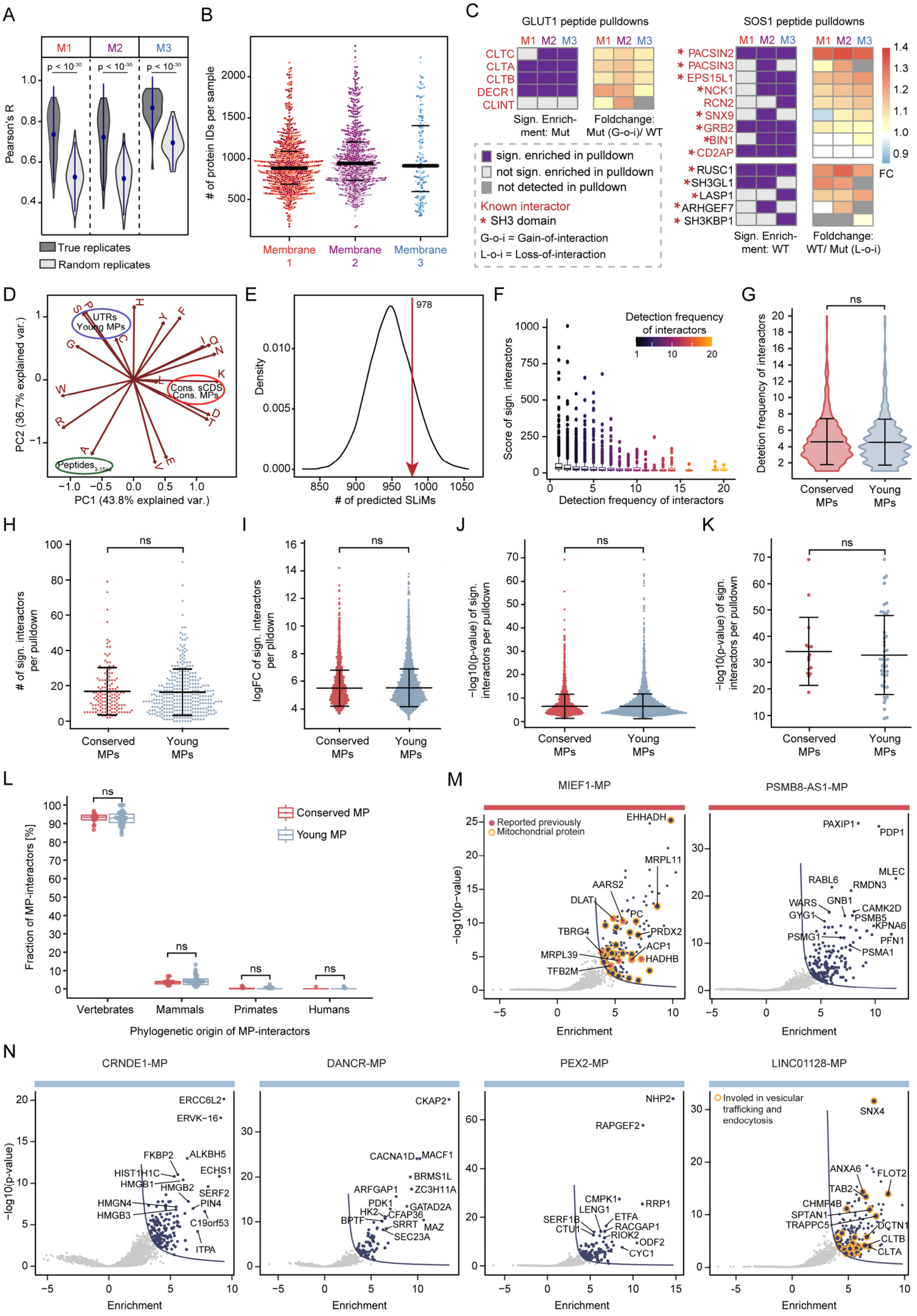

Figure S2. Interactome profiling of microproteins translated from young sORFs with PRISMA, Related to Figure 2.

(A) Pearson's correlation of true replicates is significantly higher than correlations of random samples for each membrane as assessed by Welch's t-test. Indicated are the mean ± 2 standard deviations.

(B) Distribution of total numbers of identified proteins per membrane. Dots are colored by replicates. The horizontal lines indicate 25, 50 and 75% quartiles, respectively.

(C) Left: four of the five known interactors of the GLUT1 gain-of-interaction (G-o-i) mutant peptide can consistently be detected across all three biologically distinct membranes M1, M2 and M3. Fold change (FC) of LFQ intensity of GLUT1 gain-of-interaction (G-o-i) versus wild type (WT) peptide: as expected, known interactors are enriched in G-o-i peptide pulldown (FC > 0) showing that depicted interactors bind preferentially to the mutant sequence in comparison to the wild type sequence. Right: up to eight of the nine known interactors as well as five novel SH3-domain-containing interactors of SOS1 wild type peptide can be detected across the three biologically distinct membranes M1, M2 and M3. Fold change of LFQ intensity of SOS1 WT versus Loss-of-interaction (L-o-i) mutant: as expected, most known interactors are enriched in the WT peptide (FC > 0) showing that depicted interactors bind preferentially to the WT sequence.

(D) Principal component analysis (PCA) of the amino acid frequencies of sequences included in the PRISMA dataset (45 young microproteins, 15 conserved microproteins, 221 small peptides), 271 arbitrary peptides derived from *in silico* translation of untranslated regions from 5' UTRs and lncRNAs, and 379 conserved annotated microproteins (sCDS). Frequencies were determined by calculating the number of occurrences of each amino acid per group, divided by the summed length of the sequences of each group. Methionines were excluded from the frequency calculations.

(E) Density plot with the distribution of the numbers of predicted SLiMs in 10,000 sampled sets of shuffled amino acid sequences for each of the 45 young microproteins included in the PRISMA screen. The number of SLiMs predicted in the true set of 45 young microproteins is depicted with a red arrow (978) and is not statistically different from the distribution of the number of SLiMs in shuffled sequences (Pearson's χ^2 test, p-value = 0.16).

(F) Boxplot of detection frequencies of microprotein interactors. The detection frequency of interactors describes in how many microprotein interactomes a single interactor was detected. The detection frequency decreases with the interaction score of the interactor (defined as product of p-value and fold change).

(G-K) Comparisons of **(G)** detection frequency of interactors, **(H)** number of interactors, **(I)** fold change of interactors, **(J)** p-value of interactors, and **(K)** p-value of top interactors per microprotein between conserved (n = 15) and young (n = 40) microproteins show no significant differences. Assessed by two-tailed student's t-test. Horizontal lines indicate the mean ± standard deviation.

(L) Phylogenetic origins of interactors of conserved and young microproteins. There is no significant difference when fractions of interactors per phylogenetic age are compared between young and conserved microproteins (Wilcoxon rank sum test, FDR adjusted > 0.05). For both, the majority of interactors (> 90%) originated in vertebrates.

(M) Volcano plots with interactome results of the conserved and annotated mitochondrial sCDS MIEF1-MP and the conserved microprotein PSMB8-AS1-MP (interactors from all tiles are summarized). Known interactors and novel mitochondrial interactors of MIEF1-MP are highlighted in pink and with an orange circle, respectively.

(N) Volcano plots with interactome results of the four evolutionarily young microproteins CRNDE1-MP, DANCR-MP, PEX2-MP and LINC01128-MP (interactors from all tiles are summarized). Interactors of LINC01128-MP that are involved in vesicular trafficking and/ or endocytosis are highlighted with an orange circle

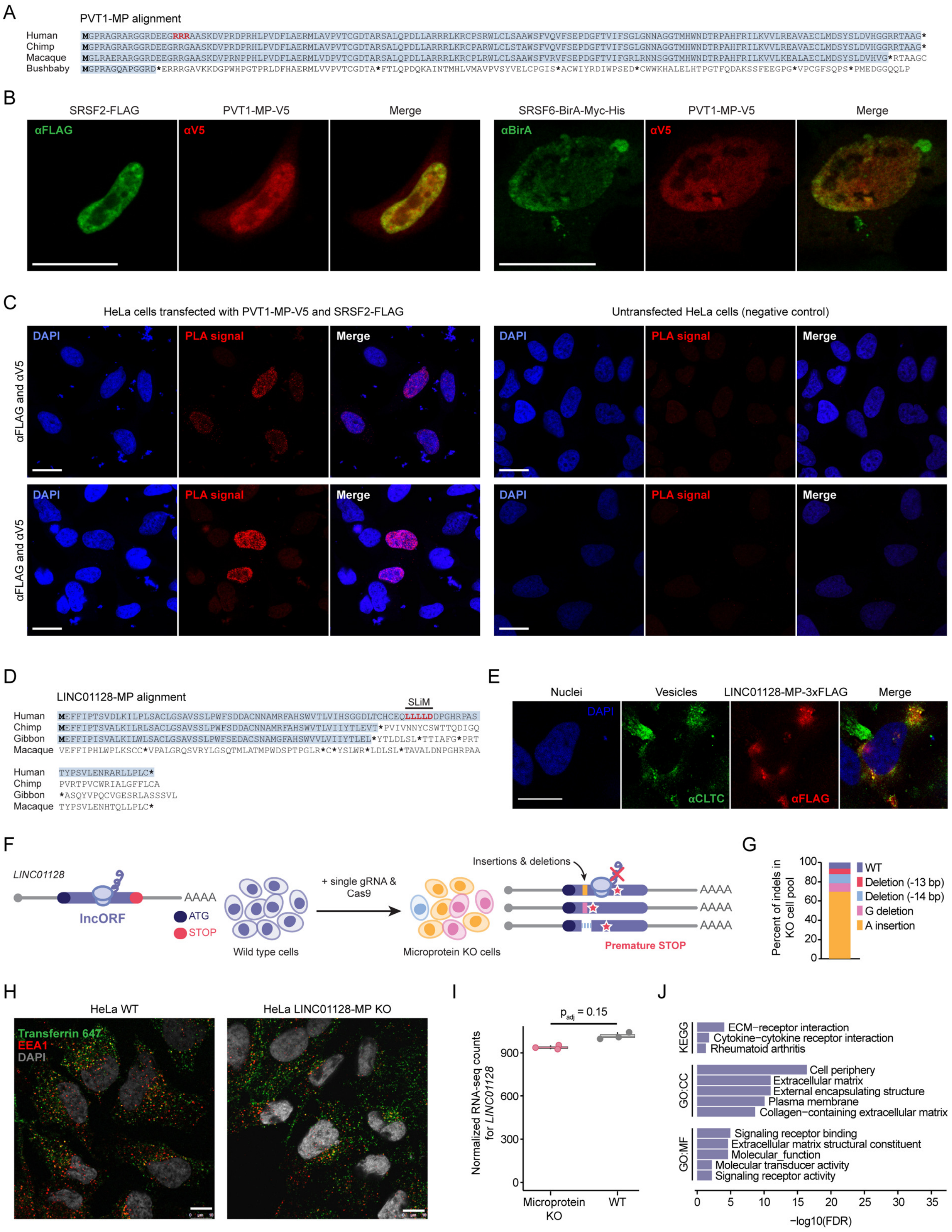

Figure S3. Short linear motifs (SLiMs) may drive microprotein-protein interactions, Related to Figure 3.

(A) Amino acid sequence alignment of the human microprotein PVT1-MP and its orthologous regions in three selected primate genomes. The stretch of three arginines that potentially mediate PVT1-MP interactions with splicing factors is highlighted in red. Intact open reading frame structures are highlighted in light blue.

(B) Partial co-localization of V5-tagged PVT1-MP with FLAG-tagged SRSF2 (left) and BirA-tagged SRSF6 (right) after overexpression in HeLa cells. PVT1-MP was stained with anti-V5 antibody, SRSF2 with anti-FLAG antibody and SRSF6 with anti-BirA antibody. Scale bars represent 20 µm.

(C) Proximity ligation assay (PLA) with anti-V5 and anti-FLAG antibodies in HeLa cells transfected with V5-tagged PVT1-MP and FLAG-tagged SRSF2 (left) and untransfected HeLa cells as negative control (right) to complement **Figure 3G**. Red fluorescent spots indicate PVT1-MP-V5 and SRSF2-FLAG interactions. Cell nuclei were stained with DAPI (blue). Scale bar represent 20 µm.

(D) Amino acid sequence alignment of the human microprotein LINC01128-MP and its orthologous regions in three selected primate genomes. Intact open reading frame structures are highlighted in light blue. The C-terminal region that carries the clathrin box motif (highlighted in red) is specific to humans.

(E) FLAG-tagged LINC01128-MP co-localizes with clathrin heavy chain protein (CLTC) after overexpression in HeLa cells. Single channel images to complement images in **Figure 3I**. Cell nuclei were stained with DAPI, CLTC with anti-CLTC antibody and LINC01128-MP with anti-FLAG antibody. Scale bar represents 20 µm.

(F) Schematic of knock-out strategy to disrupt the sORF encoding LINC01128-MP using CRISPR/Cas9 in HeLa cells. Non-homologous end joining (NHEJ) following the Cas9-mediated double-strand break leads to insertions and deletions (indels) that cause premature STOP codons and ultimately a disruption of the microprotein-encoding sORF. For RNA-seq and endocytosis experiments, a pool of cells carrying different indels was used.

(G) Proportion of indels within the CRISPR/Cas9 targeted cell pool based on RNA-seq data. The analysis was based on 76 RNA-seq reads covering the region of interest within *LINC01128*. Displayed is the average of three replicates.

(H) Representative images of fluorescently labeled transferrin (green) and EEA1 (red) detection in HeLa wild type and LINC01128-MP knock-out (KO) cells complementing **Figure 3J**. Cell nuclei were stained with DAPI (gray) and EEA1 with anti-EEA1 antibody. Scale bar represents 10 µm.

(I) Boxplots with DESeq2-normalized RNA-seq counts of *LINC01128* transcripts in HeLa wild type and LINC01128-MP-KO cells. Genome-wide corrected p-values are given.

(J) GO enrichment analysis of genes differentially expressed upon LINC01128-MP-KO in HeLa cells. The top five significant terms are plotted per group.

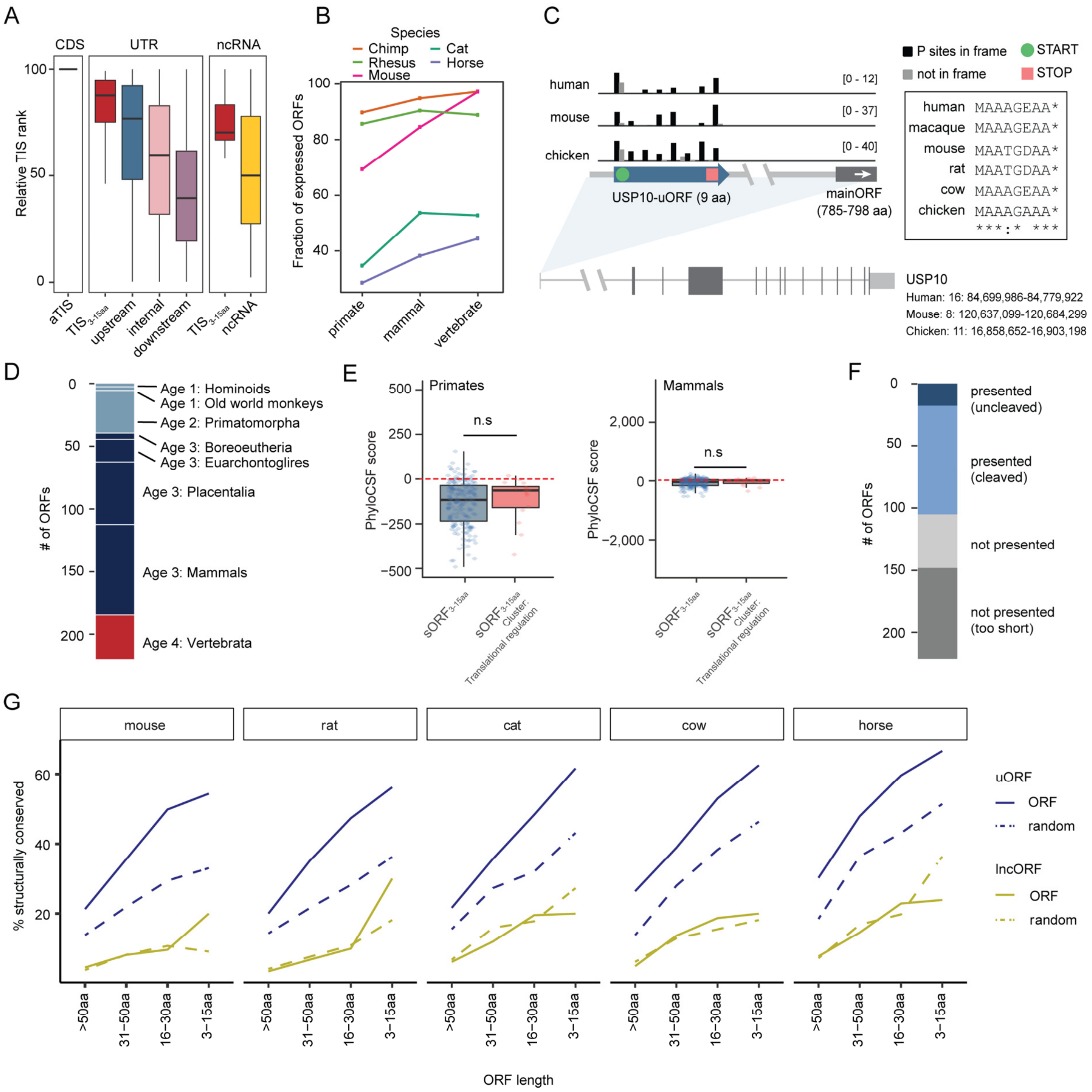

Figure S4. sORFs smaller than 16 aa (sORFs$_{3\text{-}15aa}$) are highly translated in multiple tissues and often conserved across mammals, Related to Figure 4.

(A) Results of all sORFs$_{3\text{-}15aa}$ analyzed with TIS (translation initiation site) transformer[3]. The y-axis represents the relative rank of the TIS compared to the rest of ATGs in the respective transcript. A rank of 100% means that the TIS is the ATG predicted with the highest probability. aTIS: annotated TIS. p-values calculated by Wilcoxon test, in UTRs: TIS$_{3\text{-}15aa}$ vs. upstream: $p = 1.29*10^{-9}$; TIS$_{3\text{-}15aa}$ vs. internal: $p = 9.37*10^{-37}$; TIS$_{3\text{-}15aa}$ vs. downstream: $p = 5.17*10^{-82}$; in ncRNAs: TIS$_{3\text{-}15aa}$ vs. ncRNA: $p = 0.04$.

(B) Fraction of human sORFs$_{3\text{-}15aa}$ (n = 221) expressed in chimpanzee, rhesus macaque, horse, mouse, and cat transcriptomes by conservation category.

(C) Genomic view and sequence alignment of the highly conserved USP10-uORF locus in three species[1].

(D) Conservation of ORF structures based on alignment of the 221 sORFs$_{3\text{-}15aa}$.

(E) Boxplots with PhyloCSF scores calculated across all aligned primate (left) and mammalian (right) genomes, for all sORFs$_{3\text{-}15aa}$ and a subset of sORFs$_{3\text{-}15aa}$ related to protein translation (see also **Figures 6A-D** and **Figures S6A-F**). The distribution of PhyloCSF scores for all sORFs$_{3\text{-}15aa}$ is not

statistically different compared to the subset of translational sORFs$_{3\text{-}15aa}$ (Wilcoxon one-sided signed rank test p-value$_{primates}$ = 0.77 and p-value$_{mammals}$ = 0.32).

(F) Prediction of MHC presentation of the peptides$_{3\text{-}15aa}$ translated from sORFs$_{3\text{-}15aa}$ by MHCpan-4.1[4].

(G) Fraction of uORFs (n = 3,286, including novel sORFs$_{3\text{-}15aa}$, blue) and lncORFs (n = 2,225, including novel sORFs$_{3\text{-}15aa}$, yellow) with conserved structures per length interval, for five selected mammalian species. For comparison, non-translated random ORF sequences were sampled from the same transcript regions (5' UTRs or lncRNAs, dashed line). See also **STAR Methods**.

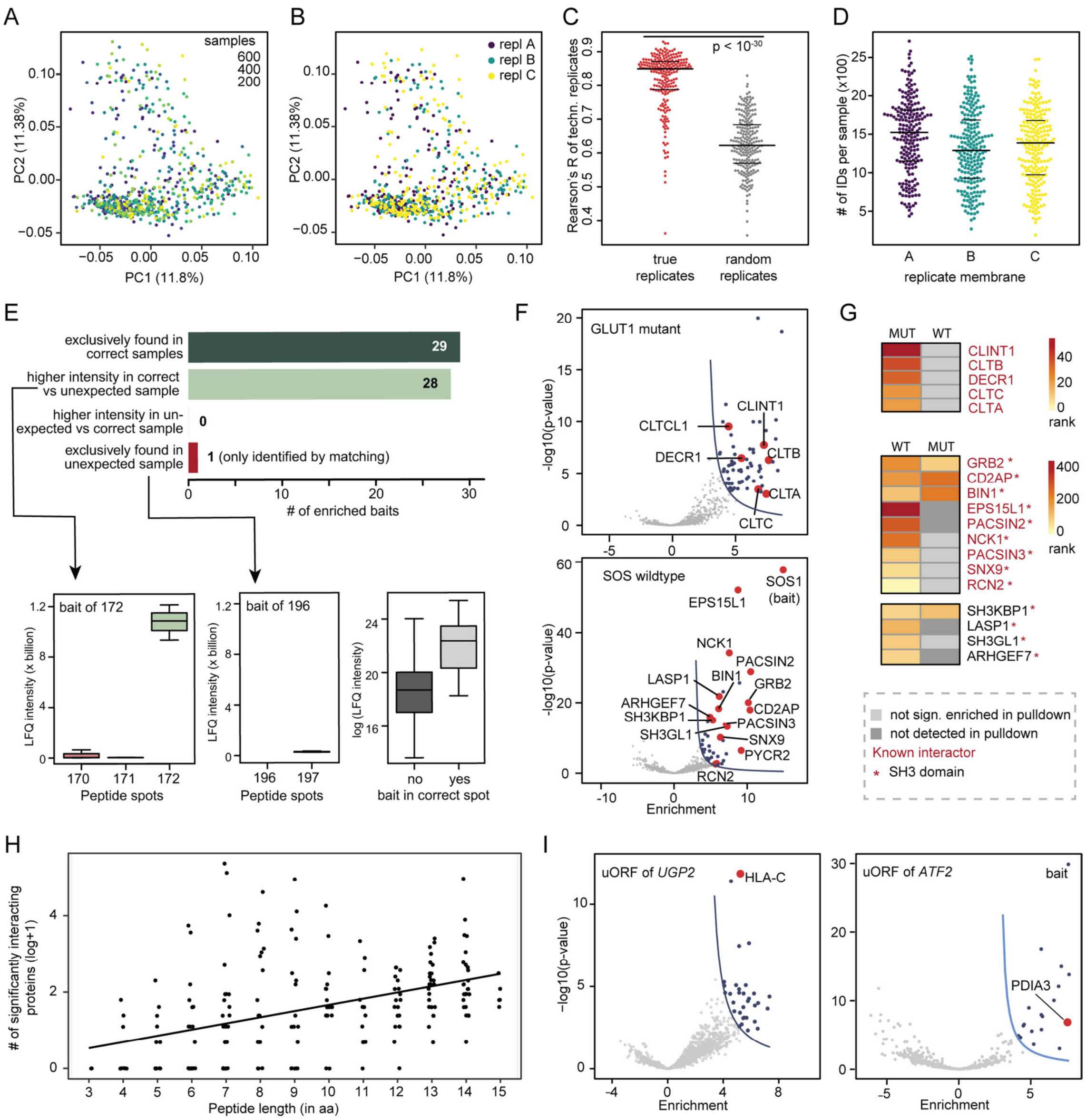

Figure S5. Peptides encoded by sORFs$_{3\text{-}15aa}$ have distinct interaction profiles, Related to Figure 5.
(A) PCA analysis of all individual samples of all replicates (colored by number) analyzed by PRISMA showing no bias.

(B) PCA analysis of the three replicates per PRISMA sample (colored by replicate) showing no bias.

(C) Pearson's correlation of true PRISMA replicates is significantly higher than correlations of random samples as assessed by Welch's t-test. The horizontal lines indicate 25, 50 and 75% quartiles, respectively.

(D) Distribution of total numbers of identified proteins per replicate. The horizontal lines indicate 25, 50 and 75% quartiles, respectively.

(E) Analysis of bait identification (**STAR Methods**).

(F) Volcano plots of GLUT1 mutant (top) and SOS1 wild type (bottom) with known interactors highlighted. All five known interactors of GLUT1 mutant were detected in the mutant and not in the wild type (see also **Figure S5G**). All nine known interactors of SOS1 wild type and four novel interactors with SH3-domain were detected in the wild type and mostly not in the mutant (see also **Figure S5G**).

(G) Top: all five known interactors of GLUT1 mutant, colored by their interaction ranks (calculated as product of fold change and p-value). As expected, the interactors are significantly binding to the mutant and not to the wild type. Bottom: all nine known interactors of SOS1 wild type and four novel interactors with SH3-domain, colored by their ranks. As expected, the interactors bind significantly to the wild type and mostly not to the mutant. An interactor that is not significantly enriched in a specific pulldown is depicted in light gray, an interactor that is not found at all in the respective pulldown is colored in dark gray.

(H) Number of significantly interacting proteins per peptide$_{3\text{-}15aa}$ by peptide length, baits were excluded.

(I) Volcano plots with the interactomes of the uORF-peptides$_{3\text{-}15aa}$ of *UGP2* (left) and *ATF2* (right). Highlighted are two proteins that are involved in MHC presentation. Both uORF-peptides are predicted to be presented on MHC *in silico* and ATF2-uORF was additionally detected in immunopeptidomics datasets (**Table S4**).

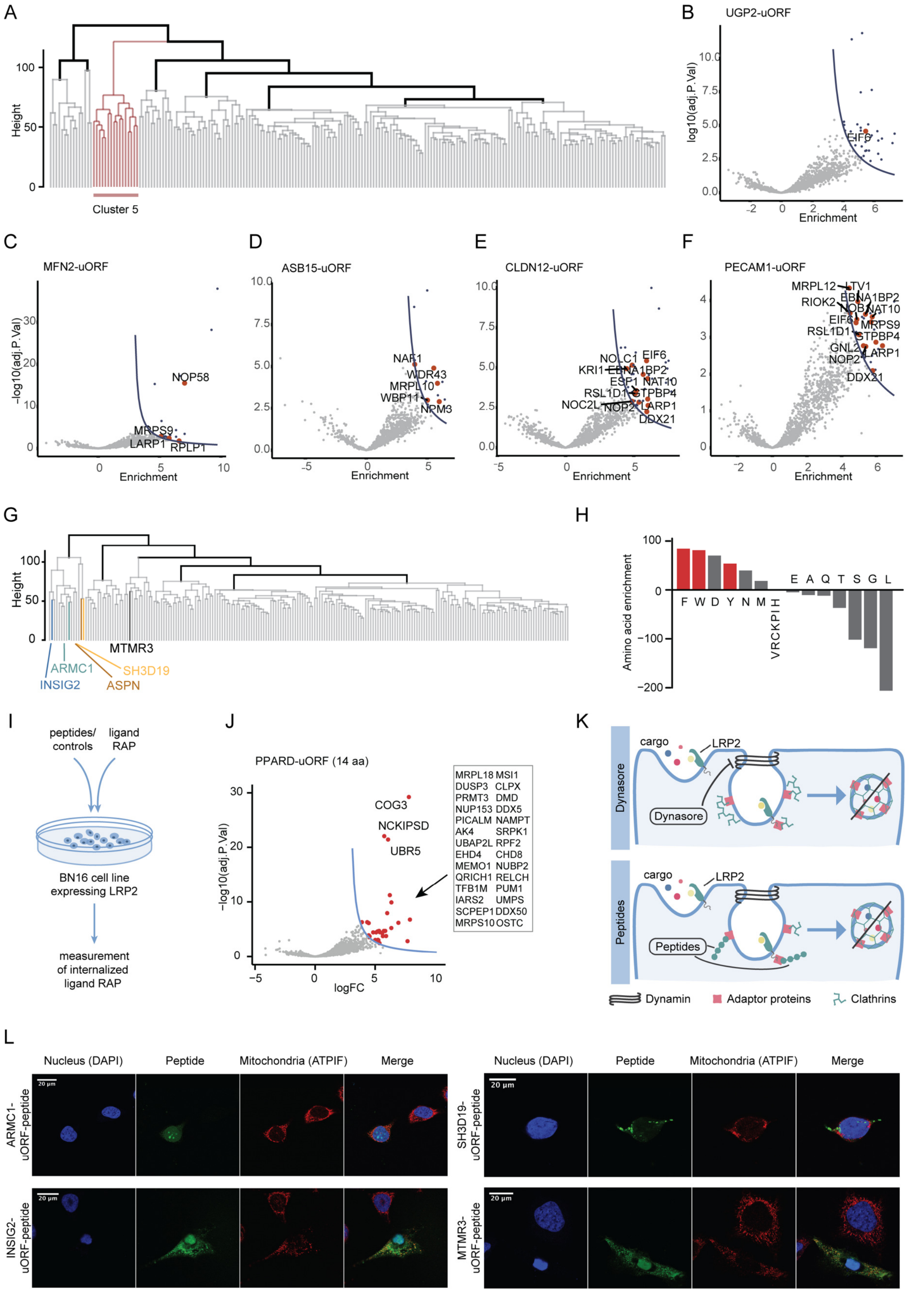

Figure S6. Peptide interactomes can predict modulators of cellular function, Related to Figure 6.

(A) Cluster of ribosome-binding peptides$_{3\text{-}15aa}$ highlighted in the global clustering overview.

(B-F) Volcano plots presenting interactomes of the five peptides$_{3\text{-}15aa}$ used in the luciferase assay. Interacting proteins highlighted in red are associated with the GO terms *translation* (GO:0006412) and *ribosome assembly* (GO:0042255).

(G) Localization within the global cluster overview of the four AP-binding uORF-peptides$_{3\text{-}15aa}$ translated from *ASPN, SH3D19, INSIG2* and *ARMC1*, as well as the uORF-peptides$_{3\text{-}15aa}$ translated from *MTMR3* that interacted with clathrins.

(H) Relative amino acid composition of the four endocytosis-related uORF-peptides$_{3\text{-}15aa}$ of *ASPN*, *SH3D19*, *ARMC1* and *INSIG2*. The enrichment is calculated by the formula: 100 - (% aa in the four endocytosis-peptides*100 / % aa in all peptides). Highlighted in red are aromatic, hydrophobic amino acids.

(I) Schematic overview of the endocytosis assay in BN16 cells. We selected the three uORF-peptides$_{3\text{-}15aa}$ of *INSIG2*, *ARMC1* and *SH3D19* for the assay, as the uORF-peptides$_{3\text{-}15aa}$ of *ASPN* also bound several proteins related to RNA-binding and splicing (**Figure 6E and Table S4**). For the assay, we measured the uptake of a physiological ligand (receptor-associated protein, RAP) by the potent endocytic receptor low-density lipoprotein (LDL) receptor-related protein 2 (LRP2/megalin) in Brown Norway rat choriocarcinoma (BN16) cells[5] in the presence or absence of peptide treatment.

(J) Volcano plot of the uORF-peptide$_{3\text{-}15aa}$ of the gene *PPARD* that does not bind any adaptor proteins and was used as a negative control in the endocytosis assay (**Figure 6H and 6I**).

(K) Top: schematic of how Dynasore inhibits dynamin and thereby endocytic vesicle formation. Bottom: possible mechanism for peptide-mediated inhibition of endocytosis.

(L) Immunofluorescence images of the synthetic, fluorescently labelled peptides$_{3\text{-}15aa}$ used in the endocytosis assay (**Figure 6H and 6I**). Additionally shown is the uORF-peptide$_{3\text{-}15aa}$ of *MTMR3* (**Figure 5C–5E**). All peptides have a TAT-sequence attached at the N-terminus and enter the cell. Cell nuclei were stained with DAPI and mitochondria with antibodies against ATPIF1.

Supplemental Items

A

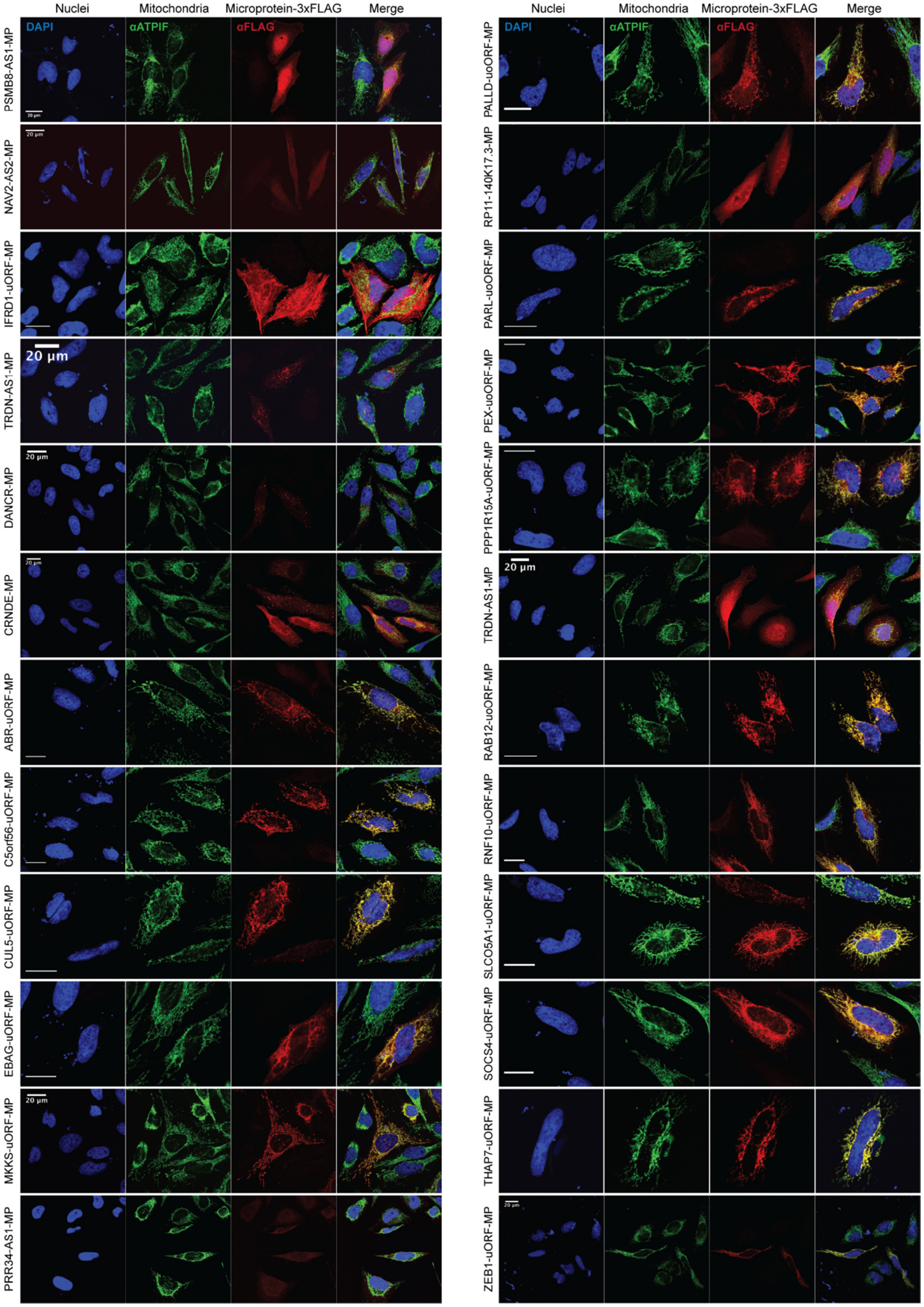

Data S1. Detection of overexpressed microproteins using immunofluorescence (IF) stainings, related to Figure 2.

(A) Twenty-six out of 31 FLAG-tagged microproteins were detected upon overexpression in HeLa cells using IF stainings. Twenty-four of the 26 are displayed above. PVT1-MP and LINC01128-MP are displayed in **Figure 3F and I; Figure S3E.** Cell nuclei were stained with DAPI, mitochondria with anti-ATPIF antibody and microproteins with anti-FLAG antibody. Scale bars represent 20 µm

Supplemental References

1. Wang, Z.-Y., Leushkin, E., Liechti, A., Ovchinnikova, S., Mößinger, K., Brüning, T., Rummel, C., Grützner, F., Cardoso-Moreira, M., Janich, P., et al. (2020). Transcriptome and translatome co-evolution in mammals. Nature *588*, 642–647.

2. Ruiz-Orera, J., Hernandez-Rodriguez, J., Chiva, C., Sabidó, E., Kondova, I., Bontrop, R., Marqués-Bonet, T., and Albà, M.M. (2015). Origins of De Novo Genes in Human and Chimpanzee. PLoS Genet. *11*, e1005721.

3. Clauwaert, J., McVey, Z., Gupta, R., and Menschaert, G. TIS Transformer: Re-annotation of the human proteome using deep learning. 10.1101/2021.11.18.468957.

4. Reynisson, B., Alvarez, B., Paul, S., Peters, B., and Nielsen, M. (2020). NetMHCpan-4.1 and NetMHCIIpan-4.0: improved predictions of MHC antigen presentation by concurrent motif deconvolution and integration of MS MHC eluted ligand data. Nucleic Acids Res. *48*, W449–W454.

5. Christensen, E.I., and Birn, H. (2002). Megalin and cubilin: multifunctional endocytic receptors. Nat. Rev. Mol. Cell Biol. *3*, 256–266.
