# Supplementary material for: Evolutionary origins and interactomes of human, young microproteins and small peptides translated from short open reading frames
Source: Mol Cell. 2023 Mar 16;83(6):994–1011.e18. doi: 10.1016/j.molcel.2023.01.023 (PMC10032668; doi:10.1016/j.molcel.2023.01.023)
Supplement: Document S2. Article plus supplemental information [file mmc7.pdf]

Molecular Cell

Resource

Evolutionary origins and interactomes of human, young microproteins and small peptides translated from short open reading frames

Graphical abstract

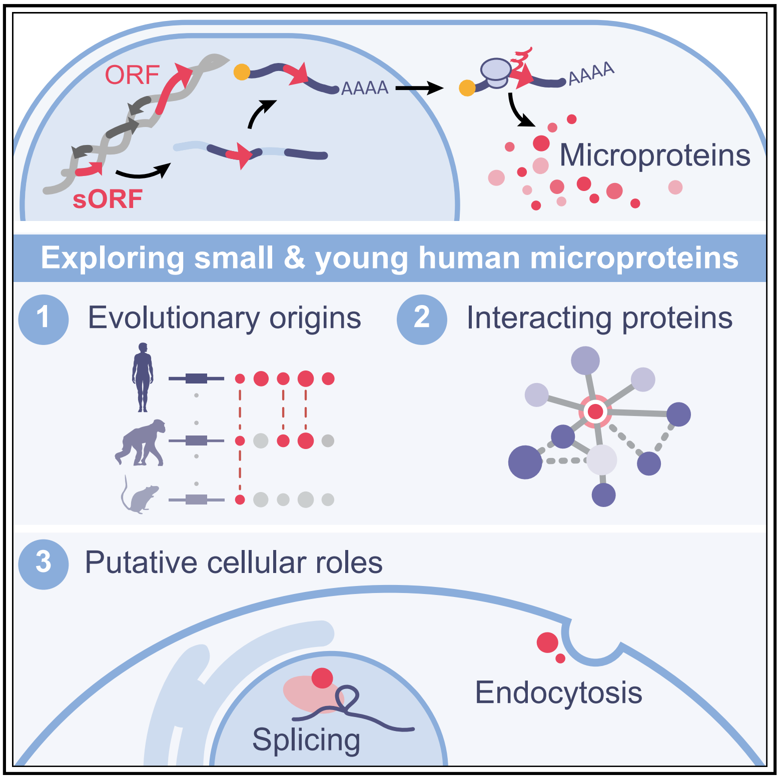

Highlights

- Most sORF-encoded human microproteins emerged in primates and often evolved *de novo*
- We identify new human sORF-encoded peptides that are smaller than 16 amino acids
- We detect interacting proteins for 266 sORF-encoded proteins in MS-based screens
- This study builds a resource to investigate small and evolutionarily young proteins

Authors

Clara-L. Sandmann, Jana F. Schulz, Jorge Ruiz-Orera, ..., Philipp Mertins, Sebastiaan van Heesch, Norbert Hubner

Correspondence

s.vanheesch@prinsesmaximacentrum.nl (S.v.H.),
nhuebner@mdc-berlin.de (N.H.)

In brief

Sandmann et al. explore the evolution and interactomes of the smallest and evolutionarily youngest members of the human proteome. They identify previously unknown human short ORF-encoded microproteins and show that conserved and young microproteins that emerged *de novo* in primates can engage with vital biological processes.

Sandmann et al., 2023, Molecular Cell *83*, 994–1011
March 16, 2023 © 2023 The Author(s). Published by Elsevier Inc.
https://doi.org/10.1016/j.molcel.2023.01.023

CellPress
OPEN ACCESS

Molecular Cell

Resource

Evolutionary origins and interactomes of human, young microproteins and small peptides translated from short open reading frames

Clara-L. Sandmann,[1,2,12] Jana F. Schulz,[1,2,12] Jorge Ruiz-Orera,[1,12] Marieluise Kirchner,[1,3] Matthias Ziehm,[1,3] Eleonora Adami,[1] Maike Marczenke,[1] Annabel Christ,[1] Nina Liebe,[1] Johannes Greiner,[1] Aaron Schoenenberger,[1] Michael B. Muecke,[1,2,4] Ning Liang,[1] Robert L. Moritz,[5] Zhi Sun,[5] Eric W. Deutsch,[5] Michael Gotthardt,[1,2,4] Jonathan M. Mudge,[6] John R. Prensner,[7,8,9] Thomas E. Willnow,[1,10] Philipp Mertins,[1,3] Sebastiaan van Heesch,[11,13,*] and Norbert Hubner[1,2,4,13,14,*]
[1]Max Delbrück Center for Molecular Medicine in the Helmholtz Association (MDC), 13125 Berlin, Germany
[2]DZHK (German Centre for Cardiovascular Research), Partner Site Berlin, 13347 Berlin, Germany
[3]Berlin Institute of Health at Charité – Universitätsmedizin Berlin, Core Facility Proteomics, 10117 Berlin, Germany
[4]Charité-Universitätsmedizin, 10117 Berlin, Germany
[5]Institute for Systems Biology, Seattle, WA 98109, USA
[6]European Molecular Biology Laboratory, European Bioinformatics Institute, Wellcome Genome Campus, Hinxton, Cambridge CB10 1SD, UK
[7]Broad Institute of MIT and Harvard, Cambridge, MA 02142, USA
[8]Department of Pediatric Oncology, Dana-Farber Cancer Institute, Boston, MA 02215, USA
[9]Division of Pediatric Hematology/Oncology, Boston Children's Hospital, Boston, MA 02115, USA
[10]Department of Biomedicine, Aarhus University, 8000 Aarhus, Denmark
[11]Princess Máxima Center for Pediatric Oncology, 3584 CS Utrecht, the Netherlands
[12]These authors contributed equally
[13]Senior author
[14]Lead contact
*Correspondence: s.vanheesch@prinsesmaximacentrum.nl (S.v.H.), nhuebner@mdc-berlin.de (N.H.)
https://doi.org/10.1016/j.molcel.2023.01.023

SUMMARY

All species continuously evolve short open reading frames (sORFs) that can be templated for protein synthesis and may provide raw materials for evolutionary adaptation. We analyzed the evolutionary origins of 7,264 recently cataloged human sORFs and found that most were evolutionarily young and had emerged *de novo*. We additionally identified 221 previously missed sORFs potentially translated into peptides of up to 15 amino acids—all of which are smaller than the smallest human microprotein annotated to date. To investigate the bioactivity of sORF-encoded small peptides and young microproteins, we subjected 266 candidates to a mass-spectrometry-based interactome screen with motif resolution. Based on these interactomes and additional cellular assays, we can associate several candidates with mRNA splicing, translational regulation, and endocytosis. Our work provides insights into the evolutionary origins and interaction potential of young and small proteins, thereby helping to elucidate this underexplored territory of the human proteome.

INTRODUCTION

Ribosome profiling (Ribo-seq)[1] has revealed the translation of thousands of short open reading frames (sORFs) in human cell lines and tissues,[2] which can result in the production of short proteins denoted as microproteins, micropeptides, or short ORF-encoded polypeptides (SEPs).[3] Many human microproteins have been selected for functional characterization based on high inter-species conservation[2,4] and a minimum amino acid (aa) size. However, evolutionarily young microproteins can have biological roles and have for instance been implicated in cell survival,[5–7] human brain development,[8] and cancer.[9–11] Similarly, a lower size cutoff for protein investigations seems unjustified: peptides as small as 11 aa can control morphogenetic development in insects[12–14] and muscle metabolism in humans,[15] respectively, and short bioactive peptides cleaved from bigger precursor proteins can function as peptide hormones.[16] These examples suggest that many more human sORFs encoding young microproteins, as well as very short peptides, may have yet unknown biological roles.

Here, we investigated these two underappreciated elements of the human proteome. To this end, we first analyzed the

 © 2023 The Author(s). Published by Elsevier Inc.
This is an open access article under the CC BY license (http://creativecommons.org/licenses/by/4.0/).

CellPress
OPEN ACCESS

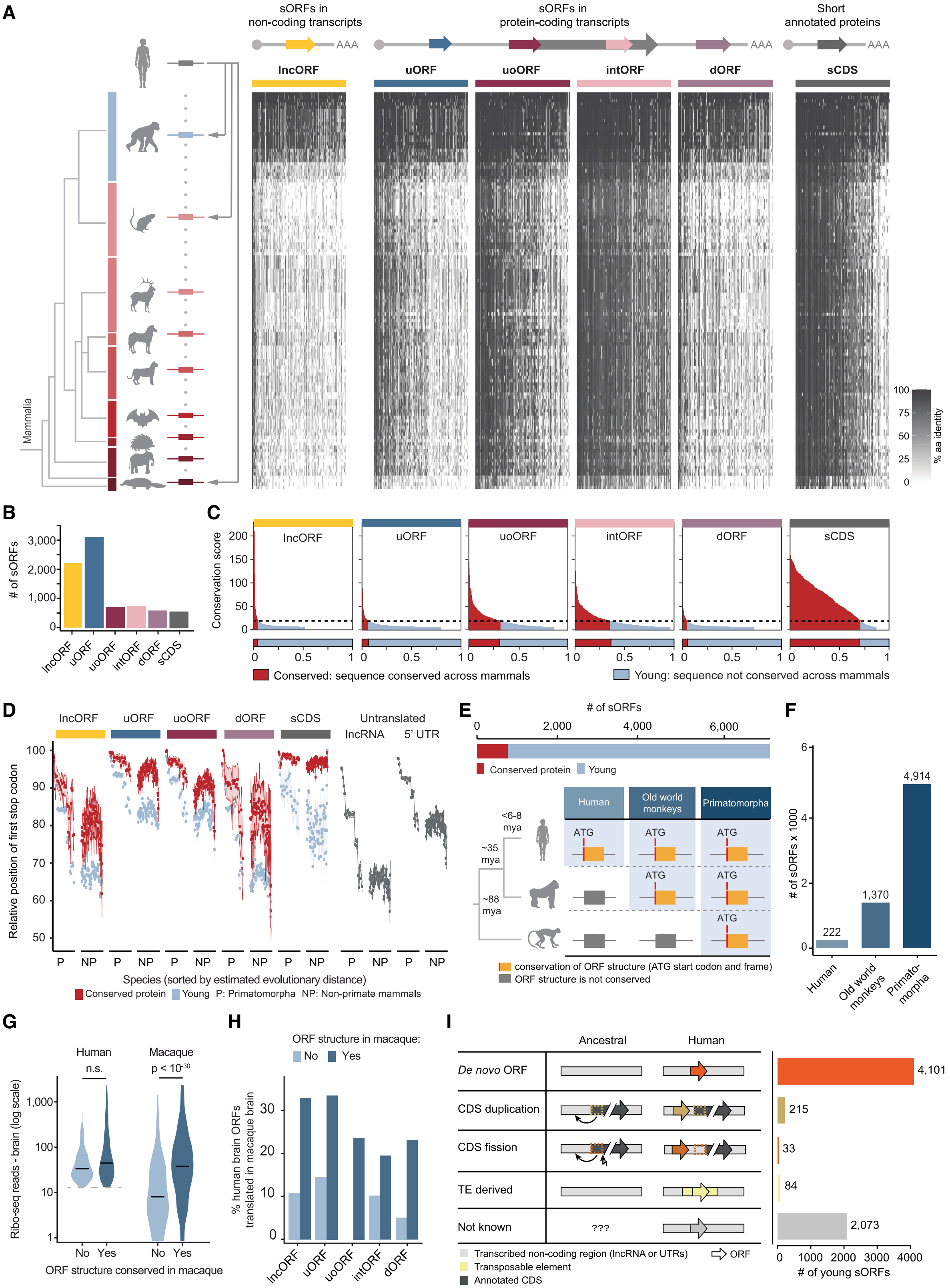

(legend on next page)

conservation and evolutionary mechanisms of origin of 7,264 human-translated Ribo-seq ORFs included in a recently published GENCODE reference catalog.[2] We found that almost 90% were evolutionarily young, of which 4,101 emerged *de novo* from ancestral non-coding regions.

To gain first insights into the potential bioactivity of these young microproteins, we selected 45 microproteins of recent evolutionary origin from the set of 7,264 sORFs and performed a high-throughput protein interaction screen on peptide matrix (PRISMA).[17–20] For this approach, proteins are divided into short peptides that are synthesized onto cellulose membranes and incubated with a protein lysate. The protein interactome of each peptide is then analyzed by mass spectrometry (MS), making PRISMA well suited to detect interactions mediated by short linear motifs (SLiMs) within disordered protein regions,[17–20] which are common in microproteins.[21]

Since the GENCODE Ribo-seq ORF catalog[2] introduced a lower length cutoff of 15 aa, we next analyzed previously published Ribo-seq datasets of human tissues[22,23] and performed (targeted) MS searches to identify and validate translated sORFs potentially encoding peptides below this cutoff (denoted $sORFs_{3–15\ aa}$ and $peptides_{3–15\ aa}$, respectively). We identified 221 translated $sORFs_{3–15\ aa}$, including 38 with endogenous peptide-level evidence, and showed that many of these are also translated in rodents and conserved across mammals. We designed an additional PRISMA screen that incorporated each of these 221 $peptides_{3–15\ aa}$.

With our two PRISMA screens, we identified a number of recently evolved microproteins and very small peptides that interacted with proteins involved in cellular processes including splicing, translational regulation, and endocytosis. In line with these interactome results, we performed independent cellular assays that indicated that several candidates can modulate translation and endocytosis, respectively.

We here present evolutionary and interactome analyses of two underexplored parts of the human proteome. Our study can serve as a resource and blueprint to investigate the cellular roles of young and small human proteins that are being detected at a rapid rate but have been difficult to characterize.

RESULTS

Most human sORFs are young and have emerged *de novo*

Recently, a community-driven effort supported by major gene and protein annotation projects (GENCODE-Ensembl, UniProt, HGNC, PeptideAtlas, and HUPO) produced a reference catalog of human Ribo-seq ORFs.[2] This catalog comprises 7,264 human sORFs longer than 15 aa (average length 43 aa) found in presumed long non-coding RNAs (lncRNAs), untranslated regions (UTRs), and alternative mRNA coding frames (Figures 1A and 1B). We assessed the amino acid conservation of the putative microproteins encoded by these sORFs and compared our results with (1) a negative control set in form of length-matched sORFs sampled from UTRs and (2) a positive control set composed of 527 annotated proteins encoded by known short protein-coding sequences (sCDSs; length <100 aa, average length 76 aa) (STAR Methods).

We found that most of the putative 7,264 sORF-encoded microproteins (n = 6,506; 89.6%) lack significant protein homology across non-primate mammals and could therefore be classified as evolutionarily young (Figures 1A–1D and S1A–S1C; Table S1; STAR Methods). The remaining 10.4% were conserved across non-primate mammals—a significantly higher fraction than observed in the negative control set (1.0%) (Figure S1D). This indicated that the putative microproteins were more conserved than expected by chance. However, the fraction of conserved microproteins encoded by sORFs (10.4%) was still lower than what we observed for annotated sCDS (71.4% conserved) (Figures 1C and S1D; Table S1).

Since primatomorpha species (primates and colugos) are closely related, counterpart sequences of human sORFs are highly similar across their genomes, showing an average aa identity of 90.62% (Figure 1A). Hence, sORF-encoded microprotein homologs in primatomorpha cannot be solely identified based on *sequence* similarity since unconstrained regions have not diverged enough. Therefore, we additionally evaluated the conservation of ORF *structures* across primatomorpha for all 6,506 young sORFs by reconstructing their ancestral sequences

Figure 1. Most human sORFs are young and have emerged *de novo*

(A) Phylogenetic tree of the mammalian taxa comprising 120 mammalian species used for sORF genomic alignments (n = 7,264). sORFs were classified into lncRNA-ORFs (lncORFs), upstream ORFs (uORFs), upstream overlapping ORFs (uoORFs), internal ORFs (intORFs), and downstream ORFs (dORFs). For comparison, we included 527 sCDS. The heatmap displays the pairwise aa identity (%) of all sORFs and sCDSs (columns) across the 120 species' genomes (rows).

(B) Numbers of evaluated sORFs and sCDS separated by ORF biotype.

(C) Conservation scores (CSs) calculated across non-primate mammalian species. Dotted lines represent the CS cutoff of 8 (STAR Methods). sORFs and sCDS with (red) or without (light blue) significant protein sequence conservation are displayed below.

(D) Dot plots displaying the average and 95% confidence interval of sORF, sCDS, and untranslated ORF truncation introduced by the most upstream stop codon in the aligned counterpart regions of the sequences. sORFs are divided by biotype and conservation of aa sequences. Internal sORFs (intORFs) were not considered due to additional constraints acting to preserve the frame of the sequence.

(E) Top: total numbers of conserved (CS $\geq$ 8) and young sORFs (CS < 8). Bottom: schematic of the classification of young sORFs (n = 6,506) based on conservation of ORF structures. We defined three levels of conservation: humans, old world monkeys, and primatomorpha.

(F) Numbers of evolutionarily young sORFs per level of conservation of ORF structures.

(G) Violin plots with the numbers of human[23] (left) and macaque[23] (right) brain Ribo-seq reads mapped to human brain translated ORFs (n = 830), by absence (light blue) or presence (dark blue) of conservation in macaque. Statistical differences were assessed by Wilcoxon signed-rank test. Horizontal bars represent the median values. ns, not significant.

(H) Percentages of sORFs translated in the human brain with aligned counterpart regions translated in macaque. sORFs are divided by biotype and by the presence (dark blue) or absence (rlight blue) of conservation in macaques.

(I) Schematic of modes of sORF evolution and numbers of young sORFs per category.

CellPress
OPEN ACCESS

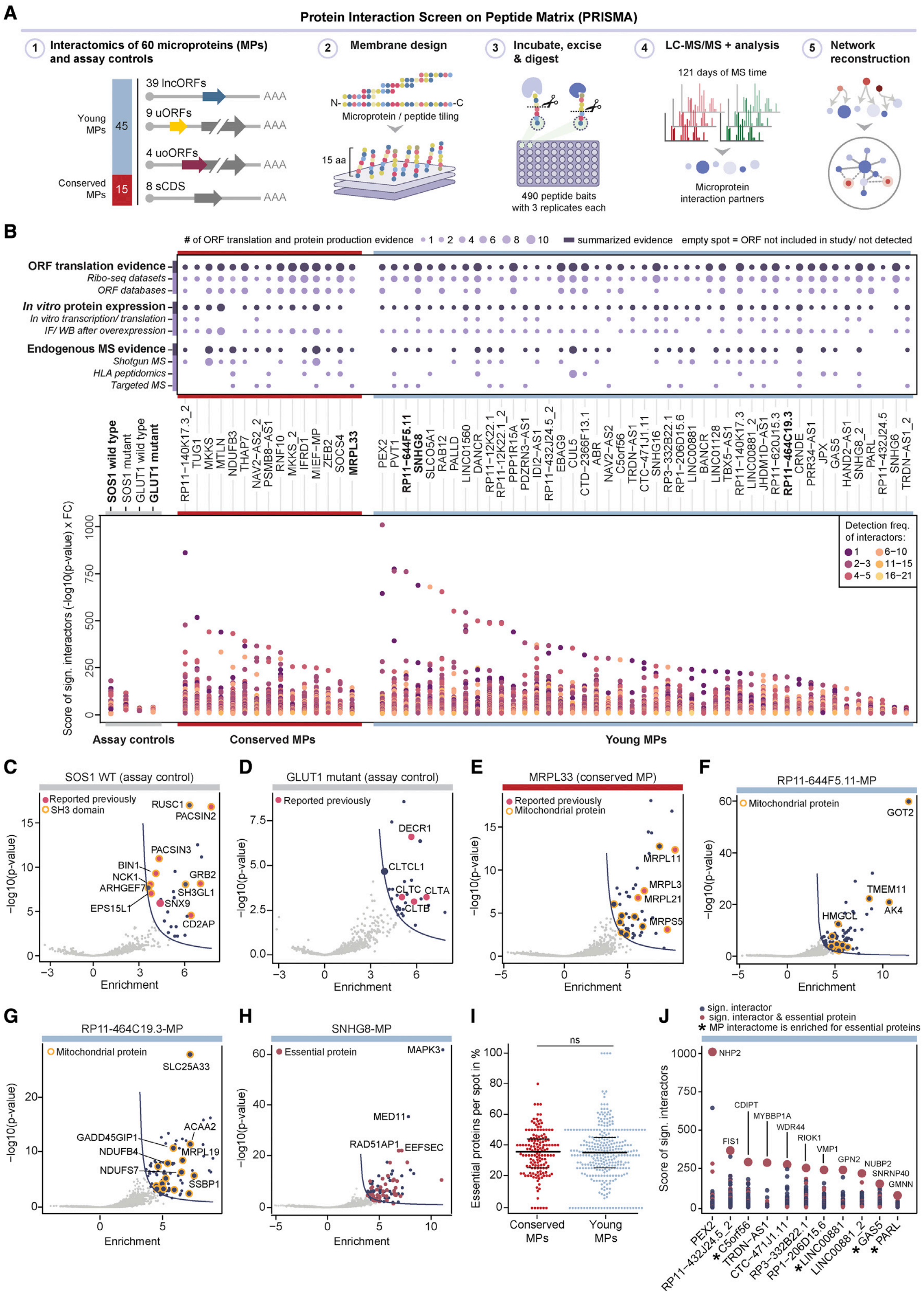

(legend on next page)

and inspecting the positional conservation of the start codon and the presence of an intact ORF (i.e., an ORF that is not truncated by premature stop codons) (Figures 1E, S1A, and S1E–S1J; STAR Methods). Within the subset of young sORFs, we found 4,914 (75.5%) with conserved structures across primatomorpha (Figures 1E and 1F). There were 1,370 ORF structures that emerged during old-world monkey evolution (after the human-macaque split), and 222 ORFs were human specific. The conservation of untranslated and translated ORF structures showed a similar phylogenetic distribution across primatomorpha lineages (Figures 1D and S1D). However, we observed a significantly higher proportion of conserved ATG initiation codons in young sORFs compared with untranslated control sequences (63.30% ± 16.14% versus 35.83% ± 12.41%, Wilcoxon signed rank test, p value = 1.45×10^{-21}, Figure S1F). These results suggest that there is significant selection pressure acting to preserve the initiation codons of young sORFs but not their frame structures.

We next compared published Ribo-seq data of human, macaque, and mouse brain samples and asked whether the absence of conserved ORF structures in non-human species led to a decrease in the levels of translation of the aligned counterpart sequences. Indeed, in comparison with regions with conserved ORF structures, we found that regions with non-conserved ORF structures exhibited lower levels of expression and ribosome occupancy, lower periodicity bias in ribosome footprints, and fewer actively translated ORFs (Figures 1G, 1H, S1G, and S1H).

When we traced the genomic changes (i.e., DNA mutations) that led to the formation of the ORFs throughout primate evolution, we found that most young sORFs (63.0%) emerged *de novo* from ancestral non-CDSs of which 162 evolved in the human lineage (Figure 1I; STAR Methods). Far less common was emergence through CDS duplication or fission of older protein-coding regions (3.3% and 0.5%, respectively) or through inserted endogenous retrovirus (ERV) elements and *Alu* repeats (1.3%). The mode of evolution could not be determined for the remainder of the investigated young sORFs (31.9%) (Figure 1I).

Interactome profiling of microproteins translated from young sORFs with PRISMA

Demonstrating that proteins engage in specific interactions with other proteins is a well-established step in gathering evidence toward putative protein functionality.[24] We applied PRISMA[17–20] to investigate the interactomes of young microproteins and the sequence motifs through which they mediate protein-protein interactions (PPIs) (Figure 2A; STAR Methods). We selected 45 recently evolved microproteins with a median length of 53 aa based on their reproducible translation across human tissues and cell lines,[2,25–27] and for having protein-level evidence[2,5,6,22,28,29] from *in vitro* translation assays (21/45), ectopic expression in cultured cells (39/45), and endogenous detection by (targeted) MS-based proteomics as reported by previously published studies[2,22,29] (30/45) (Figure 2B top panel; Table S2; Data S1; STAR Methods). Nineteen out of 45 young microproteins were translated from presumed lncRNAs that had been associated with human diseases and six affected transcriptional profiles after microprotein overexpression or knockout based on previous publications[5,6] (Table S2; STAR Methods). We included 15 conserved microproteins with a median length of 65 aa for comparison, some with known interactomes (e.g., MRPL33,[30] MIEF1-MP [MP, microprotein][31]), as well as the well-characterized wild-type (WT) and mutated peptides of SOS1 and GLUT1[18,32] as positive controls (STAR Methods).

For PRISMA, each of the selected microproteins was divided into 15 aa long, overlapping peptides (tiles) with an offset of eight aa. This way, each aa per microprotein was represented in two subsequent tiles except for the first and last eight aa of each microprotein. Following this approach and including the control peptides, we analyzed 490 peptides in total (Figures 2A, S2A, and S2B; Table S3; STAR Methods). In order to determine specific versus non-specific interactions, we compared protein identifications in each peptide spot against all other peptide spots. The ratio of each $\log_2$-fold change to its standard error was computed for each detected protein, and the resulting p values were adjusted using the Benjamini and Hochberg method to control the false discovery rate (FDR). As performed previously,[18,32] we determined the significance cutoff based on known interactors of the SOS1 and GLUT1 control peptides (STAR Methods), retaining as many known interactors and as few non-reported interactors as possible. After applying this significance filter, we retained roughly 1.4% of all identified proteins as significant binders and detected up to 89% and 80% of known SOS1 and GLUT1 interactors (Figures 2C and 2D; Table S3; STAR Methods). Additionally, we identified the expected sequence-specific interactome changes provoked by a single amino acid substitution between their WT and mutant versions (Figure S2C) and recapitulated previously reported, endogenous interactors for conserved microproteins (including MRPL33[30] and MIEF1-MP[31]; Figures 2E and S2M). These results demonstrate that PRISMA can identify sequence-specific and biologically relevant protein interactions.

Figure 2. Interactome profiling of microproteins translated from young sORFs with PRISMA

(A) Schematic of PRISMA including 60 microproteins and four assay controls.

(B) Top: protein evidence per microprotein (Table S2). Bottom: conserved (red) and young (blue) microproteins were sorted based on the highest interaction score (product of fold change and p value).

(C–H) Volcano plots with interactomes of the (C) SOS1 wild-type (WT) control peptide, (D) GLUT1 mutant control peptide, and (E) annotated mitochondrial microprotein MRPL33 (interactors from all tiles are summarized). Additional examples of conserved microproteins are shown in Figure S2M. Volcano plots of summarized interactome results of the three young microproteins (F) RP11-644F5.11-MP, (G) RP11-464C19.3-MP, and (H) SNHG8-MP, the latter being enriched for essential proteins (52 out of 106 interactors; $p_{adj} = 0.00013$; Fisher's exact test). Additional examples of young microproteins are shown in Figure S2N.

(I) Percentage of essential proteins detected in the interactomes of conserved and young microproteins. No statistical differences were found among both groups (assessed by two-tailed Student's t test). The horizontal lines indicate 25%, 50%, and 75% quartiles, respectively.

(J) Interaction scores for eleven young microproteins whose top interactor is an essential protein. Asterisks mark microprotein interactomes significantly enriched for essential proteins (assessed by Fisher's exact test, FDR < 0.05) (Table S3).

CellPress
OPEN ACCESS

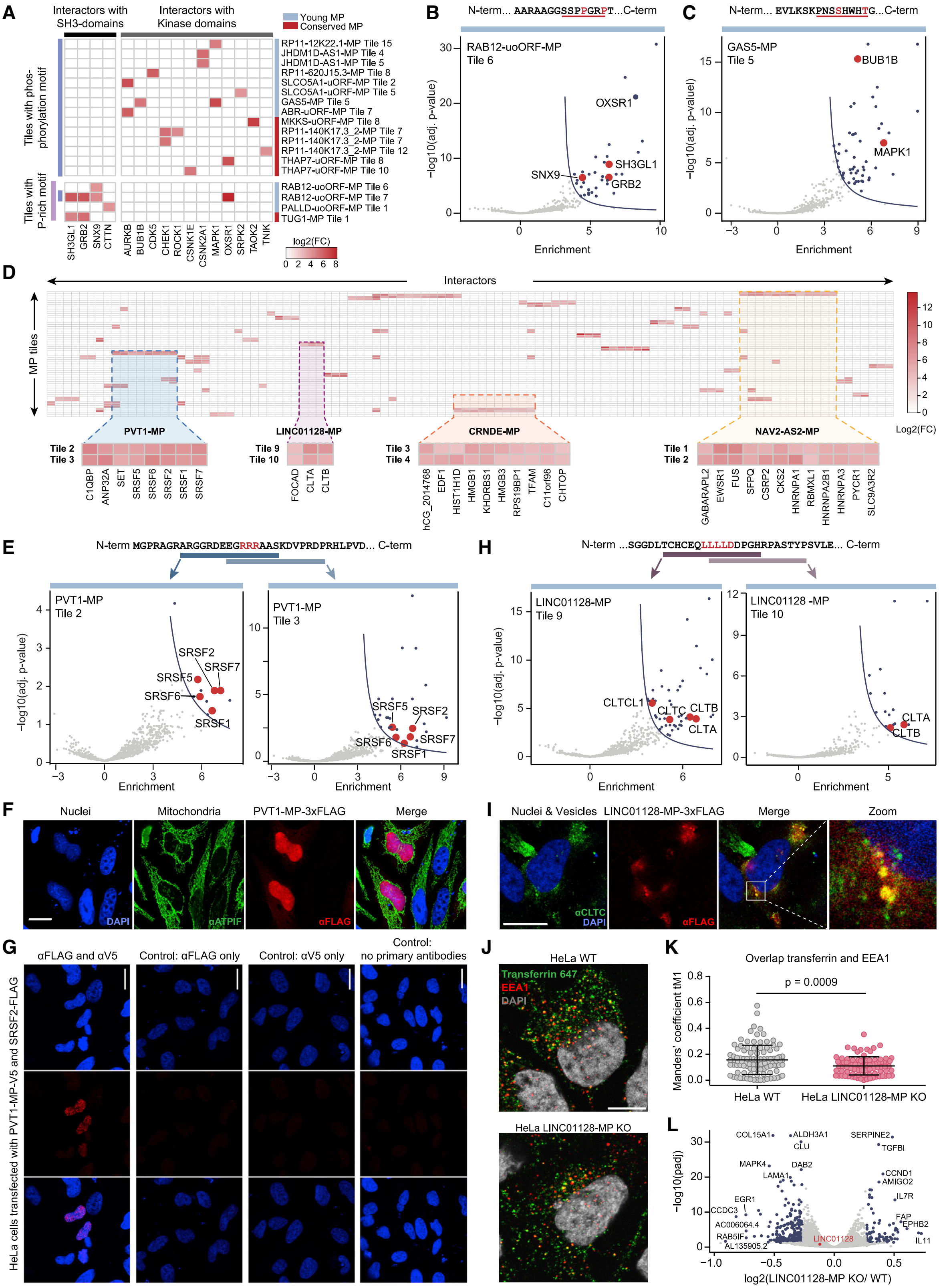

(legend on next page)

Young microproteins formed specific interactions with different classes of proteins (Figures 2B, bottom panel, 2F–2H, and S2F; Table S3). For example, the mitochondrial proteins GOT2 and SLC25A33 stood out as the most significantly enriched interactors for the microproteins RP11-644F5.11-MP and RP11-464C19.3-MP (Figures 2F and 2G), respectively, in line with their previously observed mitochondrial localization.[22] Between young and conserved microproteins, there were no apparent differences in the specificity, number, strength, or phylogenetic age of interactors (Figures S2G–S2L). Notably, young and conserved microproteins both displayed an equal capacity to interact with proteins required for cell survival[33] (Figures 2H–2J; Table S3), and the interactomes of 11 young microproteins were enriched for essential proteins (Table S3). These results suggest that young proteins have the potential to engage in vital cellular processes.

SLiMs may drive microprotein-protein interactions

SLiMs[17–19]—three to ten aa-long stretches within intrinsically disordered regions of proteins[34]—might be important contributors to the interaction potential of microproteins since microproteins are less structured and enriched for such motifs.[21,35] We identified putative disordered regions within the 60 investigated microproteins using the disorder prediction tool IUPred[36] and interrogated the eukaryotic linear motif (ELM) resource for functional sites in proteins[37] to annotate potential SLiMs (STAR Methods). Our predictions revealed 1,428 SLiMs in total, of which 429 are located within putative disordered regions of the microprotein candidates. Out of these 429, our PRISMA design covered 412 complete motifs. It should be noted that these included 87 proteolytic cleavage sites and 71 SLiMs whose binding capacity depends on additional requirements, e.g., post-translational modifications (PTMs) or free C-terminal regions (Table S3) and are thus not likely to be validated by our PRISMA screen.

Based on known protein-SLiM interactions annotated in the ELM resource,[37] we searched for matches within interactomes of each microprotein tile, i.e., if a microprotein tile carrying a SLiM bound to an interactor that is known to bind this particular motif. In total, we detected 47 matching protein-SLiM interactions (Table S3; STAR Methods). Those included proline-rich sequences within the young microproteins PALLD-uORF-MP and RAB12-uoORF-MP that interacted with proteins containing Src homology 3 (SH3) domains involved in actin cytoskeleton organization and endocytosis (Figures 3A and 3B), in a manner similar to the proline-rich SLiM of the SOS1 control peptide (Figures 2C and S2C). Furthermore, six young and three conserved microproteins interacted with kinases and harbored kinase phosphorylation and docking motifs (Figures 3A and 3C). A general enrichment of kinases in interactomes of microprotein tiles carrying such motifs could not be observed (p value = 0.079, Fisher's exact test; STAR Methods). However, we detected phosphorylated tryptic peptides after MP overexpression for two of the nine microproteins (THAP7-uORF-MP and JHDM1D-AS1-MP) (Table S3; STAR Methods), indicating that the lack of PRISMA-wide significance does not necessarily preclude the potential relevance of our observed kinase interactions.

We next focused on 190 interactors that were detected in pairs of neighboring, partly overlapping tiles within 39 microproteins (Figure 3D; Table S3). Their repeated identification strengthened these interactions and the confidence that they were mediated by the overlapping sequence. For instance, two overlapping tiles of the young and nuclearly localized microprotein PVT1-MP each bound five serine/arginine-rich splicing factors (SRSF1, SRSF2, SRSF5, SRSF6, and SRSF7; enriched GO term: *regulation of mRNA splicing*, p_{adj} = 0.011; CORUM: *spliceosome*, p_{adj} =

Figure 3. SLiMs may drive microprotein-protein interactions

(A) Heatmap with fold changes of kinases and SH3-domain-containing proteins that interact with microproteins carrying a phosphorylation/kinase-docking motif or a proline-rich motif.

(B) Peptide sequence and volcano plot with PRISMA results of a RAB12-uoORF-MP-derived peptide carrying a proline-rich motif (underlined). SH3-domain-containing proteins are highlighted in red.

(C) Peptide sequence and volcano plot with PRISMA results of the GAS5-MP-derived peptide carrying a phosphorylation motif (underlined). Kinases are highlighted in red.

(D) Heatmap with fold changes of interactors detected in two overlapping peptides within one microprotein. Only microprotein tiles that share at least three interactors are plotted (Table S3).

(E) Peptide sequences and volcano plots with PRISMA results of tile 2 and tile 3 of PVT1-MP. Splicing factors are highlighted in red.

(F) Immunofluorescence stainings of FLAG-tagged PVT1-MP after overexpression in HeLa cells. Cell nuclei were stained with DAPI, mitochondria with anti-ATPIF1 antibody, and PVT1-MP-3xFLAG with anti-FLAG antibody. Scale bar represents 20 μm.

(G) PLA in HeLa cells transfected with V5-tagged PVT1-MP and FLAG-tagged SRSF2. Red spots indicate PVT1-MP-V5 and SRSF2-FLAG interactions (additional images in Figure S3C). Cell nuclei were stained with DAPI. Controls: anti-FLAG single primary antibody only; anti-V5 single primary antibody only; both primary antibodies were omitted. As an additional control, the PLA was performed in untransfected HeLa cells (Figure S3C). Scale bar represents 20 μm.

(H) Peptide sequences and volcano plots with PRISMA results of tile 9 and tile 10 of LINC01128-MP. Tile 10 lacks the first amino acid of the clathrin box motif. Clathrins are highlighted in red.

(I) Immunofluorescence stainings of FLAG-tagged LINC01128-MP after overexpression in HeLa cells. Cell nuclei were stained with DAPI, mitochondria with anti-ATPIF1 antibody, CLTC with anti-CLTC antibody, and LINC01128-MP-3xFLAG with anti-FLAG antibody. Scale bar represents 20 μm.

(J) Representative images of fluorescently labeled transferrin (green) and EEA1 (red) detection in HeLa WT and LINC01128-MP knockout (KO) cells. Cell nuclei were stained with DAPI (gray) and EEA1 with anti-EEA1 antibody. Scale bar represents 10 μm. Images with lower magnification are shown in Figure S3H.

(K) Beeswarm plot for quantification of transferrin and EEA1 co-localization in HeLa WT and LINC01128-MP KO cells using Manders' coefficient tM1. Each dot represents one analyzed cell. Per experiment, an average of 30 cells were quantified (n = 3). Statistical significance was determined using Student's t test.

(L) Volcano plot depicting significantly differentially expressed genes (in blue, $-0.26 \leq \log_2(FC) \geq 0.26$, p_{adj} = 0.05) in RNA-seq data of wild-type versus LINC01128-MP KO cells. *LINC01128* is highlighted in red and its transcript levels are not differentially expressed between wild-type and KO cells (p_{adj} = 0.15); also see Figure S3H.

0.009), likely provoked by a stretch of three consecutive arginines within the sequence shared by both tiles (EGRRRAAS, Figures 3D–3F and S3A). This arginine stretch, which is located in a region of low complexity close to an RGG motif[38] (Table S3), resembles basic patches found in some human but mostly viral RNA-binding proteins.[38] As cell lysates were treated with benzonase before the PRISMA binding assay, these PPIs should be direct and not mediated by RNA. To support the PRISMA results, we tested SRSF2 and SRSF6 in overexpression studies and observed a partial co-localization with PVT1-MP with both splicing factors in HeLa cells (Figure S3B). Moreover, we selected SRSF2 to perform an *in situ* proximity ligation assay (PLA). PLA is better suited to corroborate protein interactions than co-localization experiments as the PLA signal, provoked by hybridization of antibody-conjugated PLA probes, only occurs when the target proteins are in close proximity (<40 nm) to each other.[39] We observed a PLA signal in HeLa cells overexpressing V5-tagged PVT1-MP and FLAG-tagged SRSF2 but not in any of the negative controls (Figures 3G and S3C). These results indicate that the interaction between PVT1-MP and SRSF2 detected with PRISMA can indeed occur in living cells.

We also found two overlapping tiles within the young LINC01128-MP to interact with multiple clathrin heavy-chain (CLTC and CLTCL1) and light-chain proteins (CLTA and CLTB) involved in vesicular trafficking and endocytosis. The binding to CLTC and CLTCL1 may be driven by a partially shared clathrin box motif (LLLLD, Figures 3D and 3H), which is usually present in endocytic cargo adaptor proteins (APs).[40,41] Within LINC01128-MP, the motif resides in a C-terminal extension that evolved in the human lineage through the loss of a stop codon (Figure S3D). In line with the observed binding profile, LINC01128-MP localized to clathrin-rich foci (Figures 3I and S3E), and the knockdown of the presumed *LINC01128* lncRNA decreased endocytosis of epidermal growth factor (EGF) and transferrin in a previous genome-wide small interfering RNA (siRNA) screen performed by Collinet et al.[42] The microprotein had remained undetected at that time, and it was not investigated whether the effect could be attributed to the lncRNA molecule or the potential microprotein. To further investigate the role of LINC01128-MP in endocytosis and trafficking, we employed CRISPR-Cas9 to create a pool of cells carrying short insertions and deletions (indels) that lead to premature stop codons truncating the endogenous sORF encoding for LINC01128-MP (Figure S3F; STAR Methods). When assessing transferrin trafficking in WT cells and the pool of modified cells (with 93% carrying premature STOP codons, Figure S3G), we observed a similar effect as in the study by Collinet and colleagues,[42] specifically a reduction of transferrin accumulation in early endosomes by approximately 30% (Figures 3J, 3K, and S3H; STAR Methods). RNA sequencing followed by differential expression analysis revealed that the sORF-truncating mutations did not significantly alter *LINC01128* transcript levels (Figures 3L and S3I; STAR Methods) suggesting that the loss of the microprotein contributed to reduced transferrin accumulation. Although our approach reduced changes to the *LINC001128* lncRNA to a minimum, we cannot completely exclude the impact of the short indels on potential RNA-mediated roles. Notably, genes differentially expressed upon microprotein truncation are enriched for extracellular matrix proteins and components of the plasma membrane. These are involved in signaling receptor binding and growth factor activity, processes associated with endocytosis and vesicular trafficking[43,44] (Figure S3J). Combined, these observations suggest a role for LINC01128-MP in the control of intracellular trafficking and endocytosis.

sORFs smaller than 16 aa (sORFs$_{3–15\ aa}$) are highly translated in multiple tissues and often conserved across mammals

Next, we investigated the smallest potential peptides translated from independent ORFs in the human genome. For this purpose, we expanded the lower size cutoff of the currently cataloged Ribo-seq ORFs[2] to include ORFs below 16 aa in length (denoted sORFs$_{3–15\ aa}$). We reanalyzed Ribo-seq data obtained from 96 human samples of five different tissues (brain, testis, liver, kidney, and heart)[22,23] using RiboTaper[45] and ORFquant[46]-methods that leverage the 3-nt periodicity in ribosome footprints to newly identify translated ORFs. This resulted in the detection of 221 translated sORFs$_{3–15\ aa}$ (Figures 4A and 4B; Table S4) of which 69% were independently identified in at least two out of five analyzed tissues (Figure 4C). We validated the translation of 182/221 (82%) sORFs$_{3–15\ aa}$ using a probabilistic algorithm for ORF detection (PRICE[47]). We further retrieved a set of predicted translation initiation sites (TISs) from a deep learning transformer model trained on the sequence context of canonical TISs[48] and observed that TISs from sORFs$_{3–15\ aa}$ were significantly more likely to initiate translation than untranslated TISs from the same transcripts (p values ranging from 0.04 to 5.17×10^{-82}; Figure S4A; STAR Methods). The translation of the majority of these newly identified sORFs$_{3–15\ aa}$ was thus substantiated by independently developed frameworks that utilize ribosome footprint periodicity, probabilistic inference, and machine learning.

The vast majority of sORFs$_{3–15\ aa}$ (92%) were translated from 5′ UTRs of protein-coding genes (uORFs) and detected at much higher levels than the downstream primary CDS (average fold change of 3.06 ± 1.09 per tissue (Figures 4D and 4E). As the conservation of smaller sORFs is challenging to determine with approaches based on *sequence* homology,[49] we calculated the conservation of the sORF *structures* across 27 primates, 92 mammals, and three non-mammalian vertebrate species. We found that 181 out of 221 sORF$_{3–15\ aa}$ structures (~82%) were conserved across mammals, and for 36 cases, the conservation was extended to vertebrates (Figures S4B–S4D; STAR Methods). In support of this, most were found to be translated in at least two out of four mouse tissues (61%; heart, brain, liver, and testis) and two rat tissues (76%; heart and liver)[22,23,50] (Figure 4F; STAR Methods). We further validated the translation of a highly conserved peptide encoded by *USP10*-uORF in bird tissues (Figure S4C). Noticeably, sORF$_{3–15\ aa}$ structures displayed higher levels of species conservation compared with longer sORFs. This is possibly explained by their localization to 5′ UTRs, as these regions contain conserved sequences that can impose additional evolutionary constraints on sORFs$_{3–15\ aa}$ located there, as well as the reduced likelihood of disrupting substitutions truncating very short sequences (Figure S4G; STAR Methods).

To collect proteomic evidence for the peptides translated from the identified sORFs$_{3–15\ aa}$, we searched the Human Proteome

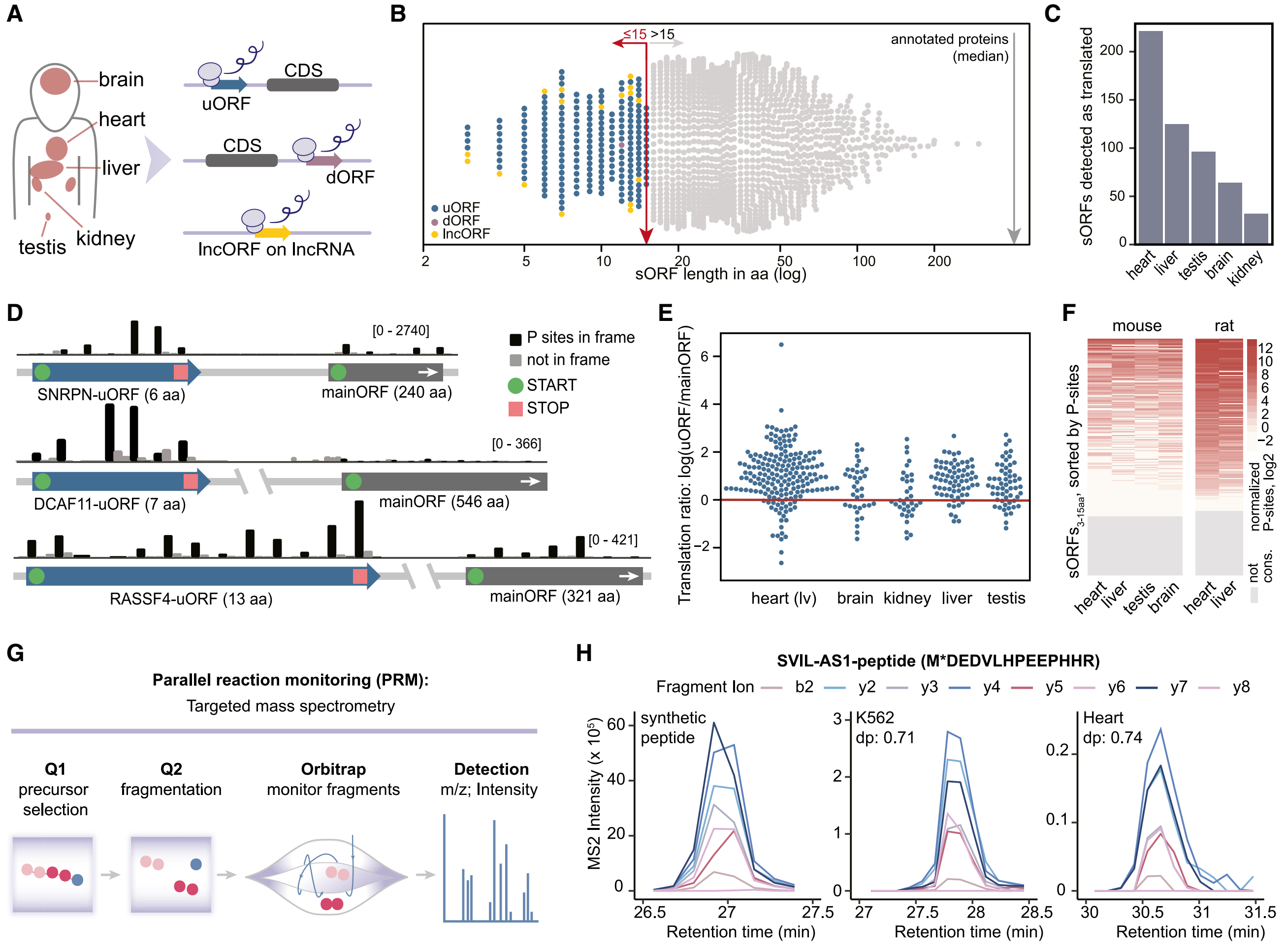

Figure 4. sORFs smaller than 16 aa (sORFs$_{3–15\ aa}$) are highly translated in multiple tissues and often conserved across mammals

(A) Detection of 221 candidate sORFs$_{3–15\ aa}$ using ribosome profiling in five human tissues.

(B) Distribution of sORF$_{3–15\ aa}$ length separated by sORF biotype and source (gray: GENCODE catalog).

(C) Numbers of sORFs called in each human tissue.

(D) Genomic view of three loci with uORFs$_{3–15\ aa}$ and the respective mainORFs. The gene orientation of *SNRPN* was reversed for clarity.

(E) Ratio of P-sites per aa of the uORFs$_{3–15\ aa}$ versus their respective mainORFs.

(F) Normalized P-sites for all candidate sORFs$_{3–15\ aa}$ whose structures are mapped and conserved in mouse (n = 166) and rat (n = 150). Gray bars represent sORFs$_{3–15\ aa}$ without conserved structures or with a length of less than 70% of the human ORF. Heatmaps are individually sorted by mean P-sites of the respective tissues.

(G) Schematic of the PRM-MS assay.

(H) Peptide sequence and chromatograms of fragment ions from synthetic and endogenous signature peptides of the SVIL-AS1-peptide$_{3–15\ aa}$ in K562 cells and the human heart. The star represents the oxidation of methionine. The dot product (dp) indicates the similarity to the matching spectrum of the synthetic peptide and ranges from 0 to 1 with higher scores indicating better similarities. We note that the detected peptide also matches an alternative microprotein isoform of SVIL-AS1 of 81 aa (Table S4).

Map[51] resource as well as global proteome[52,53] and immunopeptidomic[6,54,55] datasets. For the peptide identifications of both shotgun and immunopeptidome data, we applied an FDR filter of <0.01 on the peptide level using the reverse-sequence-based target decoy approach implemented in MaxQuant.[56] The protein FDR filter was disabled, as had been performed previously for identifications of small proteins.[45,57] Moreover, the peptides$_{3–15\ aa}$ were included in the search database of the recent human HLA 2022-09 PeptideAtlas build[29,58] (STAR Methods), and analyzed with the PeptideAtlas build pipeline[29,58] and the Trans-Proteomic Pipeline[58] using a non-specific (no protease) search strategy. In total, this led to the endogenous identification of 27 peptides from public datasets, including 16 from the HLA PeptideAtlas build. Five peptides$_{3–15\ aa}$ were identified in multiple datasets with up to four different, unique peptides. Fourteen of the peptides$_{3–15\ aa}$ detected in immunopeptidomics datasets were supported by at least one MS peptide that was *in silico* predicted to be a high-affinity binder of the major histocompatibility complex I (MHC I) (Figure S4F; Table S4).

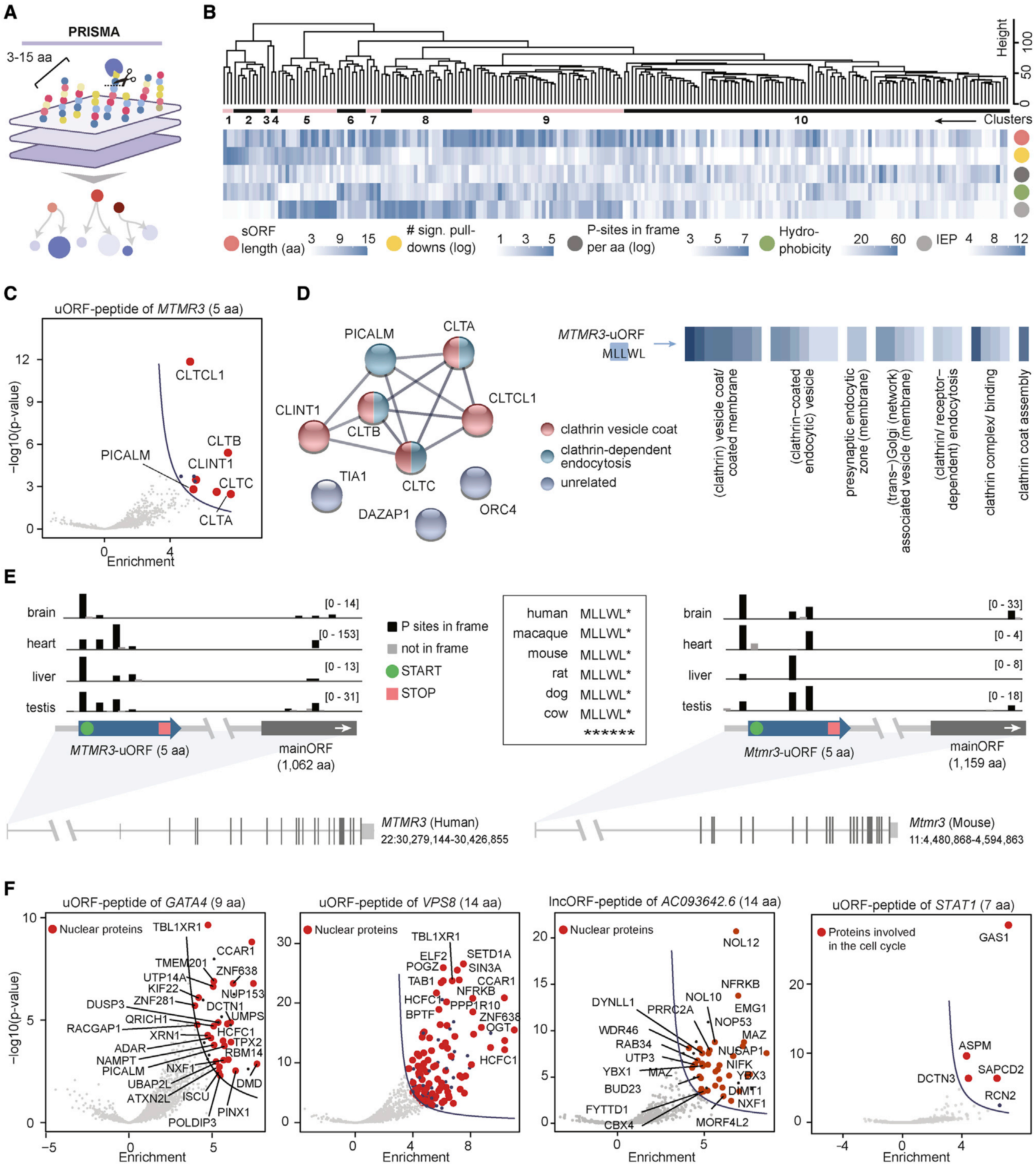

Figure 5. Peptides encoded by sORFs$_{3\text{–}15\ aa}$ have distinct interaction profiles

(A) Schematic of the PRISMA approach with all 221 sORF-encoded peptides$_{3\text{–}15\ aa}$.

(B) Hierarchical clustering of the enrichment values of all interacting proteins per peptide$_{3\text{–}15\ aa}$ (STAR Methods). Factors potentially influencing the clustering (length, number of pull-downs per peptide$_{3\text{–}15\ aa}$ [logarithmic scale], in-frame P-sites per aa [logarithmic scale], hydrophobicity, and isoelectric point) are depicted below the heatmap.

(legend continued on next page)

To enhance the endogenous detection of these very small peptides, we additionally set up a targeted MS assay (parallel reaction monitoring [PRM]) in five human hearts and three cell lines (HEK239T, K562, and HeLa) (Figures 4G and 4H; Table S5; STAR Methods). Each identification was manually confirmed based on the analytical runs of synthetic peptide mixtures as well as the internal library-based fragment ranking, and only peptides detected in at least two biological replicates with a dot product of $\geq$0.7 were considered. This yielded evidence for 18 peptides$_{3\text{–}15\ \text{aa}}$, increasing the peptide-level evidence to 38 out of 221 candidates (Tables S4 and S5).

Peptides encoded by sORFs$_{3\text{–}15\ \text{aa}}$ have distinct interaction profiles

To interrogate their ability to interact with other proteins, we synthesized all 221 peptides$_{3\text{–}15\ \text{aa}}$ in their entirety and investigated the interactomes via PRISMA (Figures 5A and S5A–S5G; Table S4). Hierarchical clustering of the enrichment values of all proteins identified in each peptide's pull down revealed several peptide features (length, hydrophobicity, and isoelectric point) that contributed to specific binding profiles (Figure 5B).

After applying stringent filtering (STAR Methods), we detected on average 16 significant interaction partners for 166 out of 221 peptides$_{3\text{–}15\ \text{aa}}$ (Figure S5H), several of which stood out for their particular interaction profiles (Figures 5C–5F and S5I). For instance, a peptide of only 5 aa translated from a highly conserved uORF within the *MTMR3* gene (peptide sequence MLLWL) bound four clathrins (CTLA, CLTB, CLTC, and CLTCL1) as well as the clathrin assembly protein PICALM and the clathrin interactor CLINT1 (Figures 5C–5E). Interestingly, the interactome of this peptide resembled that of the GLUT1 mutant peptide carrying a di-leucine motif (Figures 2D, S5F, and S5G), which is known to recruit clathrins via adapter proteins.[18] Furthermore, the interactomes of several peptides were enriched for proteins from distinct subcellular compartments, i.e., the nucleus (Figure 5F, panels 1–3), suggesting an organelle-restricted function. A seven aa long peptide encoded by the uORF of *STAT1* interacted specifically with four proteins regulating mitotic spindle assembly and cytokinesis (Figure 5F, panel 4). These four proteins are not known to interact with each other, which excludes a secondary binder effect and supports direct interaction of the uORF-peptide with these proteins.

Peptide interactomes can predict modulators of cellular function

Out of the 221 small peptide interactomes, we identified 16 hydrophilic uORF-derived peptides$_{3\text{–}15\ \text{aa}}$ rich in arginine residues that predominantly interacted with components of the translational machinery (Figures 6A–6C and S6A–S6F). In order to assess whether the presence of the translated uORF impacted downstream translation, we set up reporter assays for five of these candidates. To retain the natural sequence context of the uORFs, we inserted the candidates embedded within their endogenous 5′ UTRs in front of the luciferase reporter. As controls for each candidate, we included ATG mutants (interrupted sORF translation) as well as arginine-to-alanine mutants (translated sORF with an altered sequence) (Figure 6D, upper panel; STAR Methods). In four cases, the presence of the intact uORF significantly reduced reporter translation rates when compared against the respective uORF with mutated ATG (Figure 6D). For two candidates (ASB15-uORF and PECAM1-uORF), the arginine-to-alanine mutants reversed the observed effect, i.e., downstream translation was not impacted (Figure 6D). This indicates that the arginines are important for the repression of downstream translation of the main ORF. However, we cannot discern if the amino acid or the encoding nucleotides are responsible for the effect.

PRISMA also revealed four peptides$_{3\text{–}15\ \text{aa}}$ enriched for aromatic amino acids that bound APs involved in clathrin-mediated endocytosis (Figures 6E–6G, S6G, and S6H). Two of these peptides$_{3\text{–}15\ \text{aa}}$ contain a tandem di-hydrophobic motif that was previously proposed as a non-classical AP-binding motif.[59] When assessing the impact of the AP-binding peptides$_{3\text{–}15\ \text{aa}}$ on endocytosis *in vitro* (Figure S6I), we found that two out of three reduced the cellular uptake of a ligand by the endocytic receptor low-density lipoprotein receptor-related protein 2 (LRP2) in BN16 cells by 50% (Figures 6H and 6I). This effect was akin to the reduction observed upon treatment with dynasore (Figures 6H and 6I), a pharmacological inhibitor of clathrin-mediated endocytosis, but not upon treatment with a control peptide$_{3\text{–}15\ \text{aa}}$ that did not interact with APs in PRISMA (Figure S6J). Based on these results, we hypothesized that the binding between these uORF-peptides and APs may inhibit clathrin recruitment during the formation of new vesicles and thereby reduce endocytosis (Figure S6K).

In summary, our findings illustrate how PPIs detected by PRISMA can hint toward putative biological roles of previously unknown peptides, stimulating future efforts into the mechanistic roles of both evolutionarily young and very small peptides translated from short ORFs in the human genome.

DISCUSSION

Microproteins have gained increasing attention in recent years, but the biological significance of human evolutionarily young and very small microproteins has remained less well studied.[2,5,7,22,60–63] We aimed to address this knowledge gap by investigating the evolutionary origins and interactomes of the putative microproteins and peptides encoded by young and very small ORFs.

To define the fraction of young human microproteins, we evaluated the conservation of the amino acid sequences of over

(C) Volcano plot of the peptide encoded by *MTMR3*-uORF. Proteins assigned to the GO term *clathrin-dependent endocytosis* (GO:0072583) as well as the clathrin-binding protein CLINT1 are highlighted in red.

(D) Left: string network of all significantly bound proteins of the MTMR3-uORF-peptide. Lines indicate confidence based on experiments, databases, and co-occurrence; high confidence (0.7). Right: MTMR3-uORF-peptide sequence (di-leucine motif highlighted) and GO enrichment analysis of its interactors.

(E) Genomic view and sequence alignment of the highly conserved *MTMR3*-uORF locus in four human (left) and four mouse (right) tissues.[22,23]

(F) Volcano plots summarizing the PRISMA results of the peptides$_{3\text{–}15\ \text{aa}}$ translated from *GATA4*-uORF, *VPS8*-uORF, *AC093642.6*-lncORF, and *STAT1*-uORF.

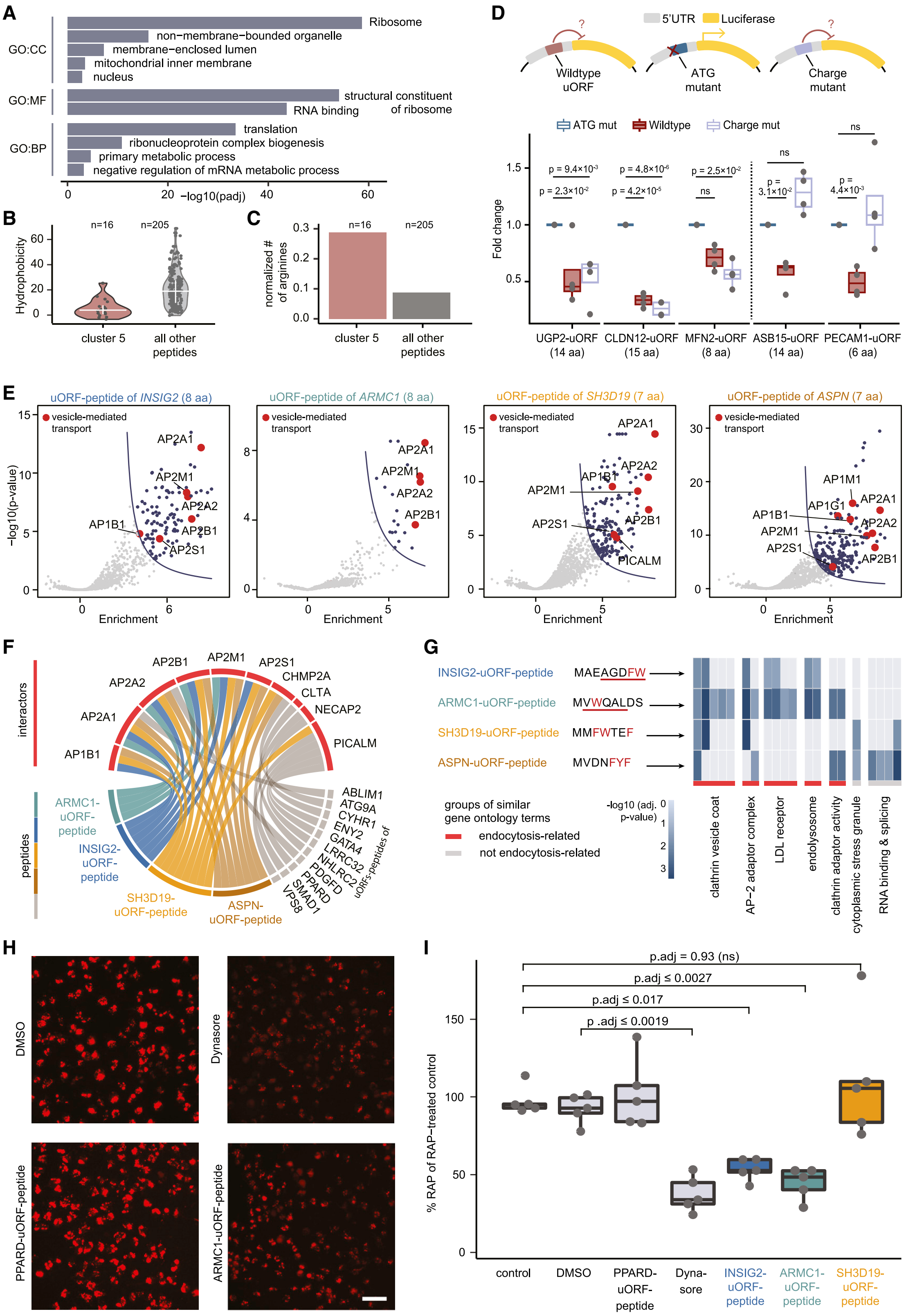

(legend on next page)

7,000 recently cataloged human sORFs across more than 90 mammalian species and found that most were not conserved to non-primate mammals. We further present a detailed resource describing the evolutionary age and mechanisms of origin of these sORFs across primatomorpha, which included the genomes of 27 different primate and colugo species. Since the genomes of these species are highly similar, standard sequence similarity search methods were not sufficient to reliably infer homology for constrained proteins and estimate their evolutionary ages.[64] We circumvented these limitations by assessing the conservation of translated sORF structures, i.e., we evaluated the conservation of the start codon and the presence of an intact ORF in the ancestor sequences of human young sORFs. We further estimated that 4,101 sORFs emerged *de novo* during primatomorpha evolution, including 162 human-specific ones. When translated into stable microproteins, this would increase the number of human-specific *de novo* microproteins reported previously[7] by an order of magnitude. This substantiates observations that sporadic protein evolution "from scratch" may occur at a higher rate than previously thought.[65–68] Of note, our evolutionary analyses are conservative because the presence of a homologous ORF sequence in other primate species does not indicate its expression per se. In primates, the extent of transcription and translation of young ORFs with conserved structures will require future studies that generate new pan-transcriptomes, -translatomes, and -proteomes from non-human primates.

In order to further investigate these novel microproteins, we employed PRISMA, which is highly suitable for the analysis of microprotein interactomes due to several technical advantages: (1) its high scalability allows the inclusion of hundreds of peptides which increases the statistical power to define significant interactions, (2) it does not rely on the ectopic expression of CDS vectors, (3) it does not require the addition of a tag (e.g., HA and FLAG), which is particularly problematic for small proteins, and (4) it does not depend on antibody-based affinity pull-downs that introduce antibody-specific background binding.

We show that young microproteins can bind proteins that are involved in diverse cellular processes, including proteins essential for cell survival. Moreover, our results illustrate how short sequence features such as SLiMs, which are prevalent within intrinsically disordered regions of microproteins,[21] may contribute to the ability of recently evolved microproteins to engage with the more conserved human proteome. This capacity to interact may be present as early as at, or shortly after their evolutionary origin, without the need to evolve larger and more complex three-dimensional structures. For example, and although we could not completely rule out potential RNA-mediated effects, LINC01128-MP KO experiments suggested that this microprotein plays a role in transferrin accumulation in early endosomes *in vitro*, potentially enabled through interactions of the microprotein's human-specific C terminus with endocytic proteins. This supports the idea that many young proteins can quickly become functional after they emerge *de novo*.[69,70] Further investigations are, however, necessary to delineate how *LINC01128* may affect endocytosis.

Furthermore, we identified 221 novel small translated sORFs$_{3–15\ aa}$ by Ribo-seq in five human tissues. We demonstrated the translation of most of them by independently developed methodologies that exploit ribosome footprint periodicity (RiboTaper,[45] ORFquant,[46] and PRICE[47]), machine learning-based inference of TISs transformer,[48] and evolutionary sequence alignments demonstrating conservation across mammals. The fact that the sORFs$_{3–15\ aa}$ were translated at high levels and the majority of their structures were conserved to rodents, as supported by Ribo-seq, might indicate biological relevance. Of note, we obtained putative peptide-level evidence for 38 out of 221 predicted very small peptides$_{3–15\ aa}$ with proteomics technologies. Each of these 38 peptides$_{3–15\ aa}$ was smaller than the smallest human peptide translated from an individual sORF annotated as protein-coding to date (MOTS-c[15]; 16 aa). We would like to point out that MS identification of such small peptides is technologically challenging[4] and the possibility of false-positive identifications cannot be completely excluded.

Following the PRISMA results obtained for newly detected peptides$_{3–15\ aa}$, we highlight a group of hydrophilic, arginine-rich uORF-encoded peptides$_{3–15\ aa}$ that bound components of the translational machinery. We observed in a luciferase reporter assay that four translated uORFs$_{3–15\ aa}$ reduced downstream translation, potentially through *cis*-mediated effects such as re-initiation inhibition[71] or ribosome stalling.[72] For two candidates, the translational inhibition of the downstream reporter seemed to depend on the peptide sequence and charge (i.e., presence of arginine residues). However, we cannot exclude that the

Figure 6. Peptide interactomes can predict modulators of cellular functions

(A) GO enrichment analysis of all interacting proteins of 16 ribosome-binding peptides$_{3–15\ aa}$ compared with all other peptides.

(B) Violin plot with hydrophobicity values of the 16 ribosome-binding peptides$_{3–15\ aa}$ compared with all other peptides$_{3–15\ aa}$. Horizontal lines indicate the mean ± standard deviation.

(C) Number of arginines of the 16 ribosome-binding peptides$_{3–15\ aa}$ compared with all other peptides$_{3–15\ aa}$, normalized to the total number of amino acids.

(D) Schematic and results of the luciferase reporter assay performed with five randomly selected ribosome-binding peptides$_{3–15\ aa}$. The significance was calculated using ANOVA and Tukey post hoc test.

(E) Volcano plots of four AP-binding peptides$_{3–15\ aa}$. Proteins assigned to the GO term *vesicle-related transport* (GO:0016192) are highlighted in red.

(F) Circos plot of all peptides$_{3–15\ aa}$ that interact with endocytic proteins.

(G) Peptide sequences of the four AP-binding peptides$_{3–15\ aa}$ (aromatic aa highlighted in red, di-hydrophobic motifs underlined) and GO enrichment analysis of their interactomes.

(H) Representative immunofluorescence images of fluorescently labeled RAP internalized by BN16 cells treated with DMSO, dynasore, PPARD- and ARMC1-uORF-peptide, respectively. Scale bar represents 200 μm.

(I) Results of the RAP endocytosis assay (five replicates per condition). Values were normalized to total protein content, and samples without RAP treatment were subtracted and then normalized to the treatment with RAP only (=100%). The PPARD-uORF-peptide, which did not bind APs, was included as a control (Figure S6J). The statistical significance was calculated using ANOVA and Tukey post hoc test.

effect stems from the underlying nucleotide change. We continued to show that members of a separate class of novel peptides$_{3\text{–}15\ aa}$ could bind APs of the endocytic machinery and were capable of reducing endocytosis levels by 50% *in vitro*. As a means to control for a possible influence that the transactivating transcriptional activator (TAT) sequence attached to our candidate peptides might have on endocytosis, we included a control peptide that did not bind any endocytosis-related proteins in the PRISMA screen. This peptide did not influence endocytosis levels, indicating that the TAT-peptide per se does likely not (solely) impact the observed effect. We hypothesize that the candidate peptides$_{3\text{–}15\ aa}$ that reduce endocytosis might hinder clathrin recruitment and vesicle formation by competitive binding to APs. Interestingly, the protein encoded by the main CDS of one of these uORF-peptides—INSIG2—is known to mediate the feedback control of cholesterol synthesis.[73] This hints that the *INSIG2*-uORF could contribute to the impact of INSIG2 on circulating cholesterol levels by regulating endocytic uptake of cholesterol-rich LDL particles, possibly indicating a co-evolution of the uORF and the main protein. In light of the great potential that similarly sized synthetic peptides have shown as pharmacological compounds,[74] these peptides$_{3\text{–}15\ aa}$ may also be exploited therapeutically as modulators of endocytosis, or new inhibitors of translation, a mechanism commonly used in antibiotics.[75]

In summary, our study provides new insights into the evolutionary origins and potential roles of evolutionarily young microproteins and very small peptides in humans. We describe motif-resolution interactomes for hundreds of human microproteins and peptides, which may serve as a basis for candidate-focused, independent validation experiments. We anticipate that these insights will set the stage for future investigations of this underexplored part of the human proteome, which will be crucial for our understanding of protein evolution, adaptation, and human-specific biology.

Limitations of the study

Although the interactions detected by PRISMA suggest that small peptides and young microproteins can take part in fundamental cellular processes, we recognize certain limitations of the assay. Longer microproteins need to be tiled into smaller segments, which leads to the loss of the natural protein context, and the impact of globular protein folds and domains on microprotein interactomes will be missed. Moreover, PRISMA employs synthetic peptides and does not recapitulate a peptide's stability, expression level, and concentration within cells. As a possible instability of the microproteins and peptides$_{3\text{–}15\ aa}$ would preclude them from building stable interactions *in vivo*, PRISMA can only yield information on possible interactions that can be used to derive functional hypotheses but need further validation. Furthermore, the use of a cell protein lysate may yield interactions that would otherwise not occur due to cellular compartmentalization, it is not possible to discriminate between direct and indirect interactions, and we lose the fraction of cell-type-specific interactions that may occur in cell types other than the cell lysate used in this study. We highlight that PRISMA works with any cell or tissue lysate that is not suitable for conventional interactome approaches, such as hard-to-transfect cells (e.g., cardiomyocytes) or (human) disease-specific clinical samples. Lastly, we did not evaluate the impact of PTMs on microprotein interactomes, which will be of interest in future studies, particularly in regard to SLiMs that depend on PTMs.[41]

STAR★METHODS

Detailed methods are provided in the online version of this paper and include the following:

- KEY RESOURCES TABLE
- RESOURCE AVAILABILITY
  - Lead contact
  - Materials availability
  - Data and code availability
- EXPERIMENTAL MODEL AND SUBJECT DETAILS
  - Cell culture
  - Human primary material
- METHOD DETAILS
  - Retrieval of sORF and control sequences
  - Whole-genome alignments across mammalian species
  - Protein sequence conservation of sORFs and sCDS
  - Conservation of sORF structures
  - Expression and translation of sORFs in mammals
  - Mode of evolution of sORFs
  - Comparison of the modes of evolution with a previous resource
  - Candidate selection for PRISMA of microproteins translated from recently evolved sORFs
  - Detection of overexpressed microproteins by immunofluorescence
  - PRISMA of microproteins translated from cataloged sORFs
  - Expression of microproteins and peptides$_{3\text{-}15\ aa}$ in HEK293 cells
  - Annotation and enrichment of essential proteins detected in microprotein interactomes
  - Phylogenetic origin of microprotein interaction partners
  - Computational prediction of disordered regions and short linear motifs within microproteins and peptides
  - Detection of protein domain-SLiM matches in PRISMA interactomes
  - Kinase-enrichment by microproteins with kinase-related SLiMs
  - Phosphorylation of kinase-binding microproteins with domain-SLiM matches
  - Gene ontology analysis
  - Co-localization analysis of PVT1-MP with SRFS2 and SRSF6
  - Proximity ligation assay
  - Generation of LINC01128-MP knock-out cells and RNA-seq analysis
  - The role of LINC01128-MP in transferrin endocytosis
  - Detection of sORFs$_{3\text{-}15\ aa}$ in human tissues
  - Conservation of sORFs$_{3\text{-}15\ aa}$ in mammalian tissues

- ○ Effect of genomic location and length in the conservation of sORF structures
- ○ Additional evidences of $sORF_{3\text{-}15\ aa}$ translation
- ○ Detection of sORF-translated $peptides_{3\text{-}15\ aa}$ in public mass spectrometry data
- ○ PRM proteomics
- ○ PRISMA of peptides translated from $sORFs_{3\text{-}15\ aa}$
- ○ Peptide properties
- ○ Analysis of CORUM complexes in interactomes of $peptides_{3\text{-}15\ aa}$
- ○ Luciferase assay for ribosome-binding peptides
- ○ Endocytosis assay for adaptor-protein-binding $peptides_{3\text{-}15\ aa}$
- ○ Immunofluorescence of $peptides_{3\text{-}15\ aa}$
- ● QUANTIFICATION AND STATISTICAL ANALYSIS

SUPPLEMENTAL INFORMATION

Supplemental information can be found online at https://doi.org/10.1016/j.molcel.2023.01.023.

ACKNOWLEDGMENTS

We thank Russ Hodge for critical discussions and editing; Sylvia Niquet, BIH Core Unit Proteomics, for technical assistance; Henrik Zauber (MDC) for implementing hydrophobicity calculations; Anje Sporbert, Anca Margineanu, Maik Schumann, Matthias Richter, and the Advanced Light Microscopy Technology Platform (MDC) and the Systems Biology Imaging Platform (MDC), especially Andrew Woehler and Marie Walde, for technical support and assistance; Janine Fröhlich for their technical assistance with co-localization experiments; the BIH/MDC Genomics Technology Platform for their support; and Jim Clauwaert and Gerben Menschaert (Ghent University, Belgium) for implementation and discussion of the TIS transformer results. N.H. was supported by a grant from the Leducq Foundation, an ERC Advanced Grant under the European Union Horizon 2020 Research and Innovation Program (AdG788970), and a British Heart Foundation and a Deutsches Zentrum für Herz-Kreislauf-Forschung grant (BHF/DZHK: SP/19/1/34461). This project has been made possible in part by grant number 2019-202666 from the Chan Zuckerberg Foundation. E.W.D., R.L.M., and Z.S. were supported by the National Institutes of Health grants R01 GM087221 and U19 AG023122 and by the National Science Foundation grant DBI-1933311. J.R.P. was supported by an Alex's Lemonade Stand Foundation Young Investigator Award (#21-23983). J.M.M. was supported by the National Human Genome Research Institute of the National Institutes of Health under award number 2U41HG007234 and the European Molecular Biology Laboratory. The content is solely the responsibility of the authors and does not necessarily represent the official views of the National Institutes of Health. Ensembl is a registered trademark of EMBL. S.v.H. was supported by a DZHK (German Centre for Cardiovascular Research) Excellence Program PostDoc Start-up Grant (81X3100106). This work was also supported by the Deutsche Forschungsgemeinschaft (DFG, German Research Foundation) under grant agreement SFB 1470 "HFpEF" (projects B03, B04, and B05) to N.H., M.G., and P.M.

AUTHOR CONTRIBUTIONS

Conceptualization, N.H., J.R.-O., C.-L.S., J.F.S., and S.v.H.; methodology, N.H., M.G., P.M., J.R.-O., C.-L.S., J.F.S., S.v.H., and T.E.W.; validation, A.C., E.W.D., M.K., M.M., R.L.M., C.-L.S., J.F.S., Z.S., and M.Z.; formal analysis, E.W.D., M.K., R.L.M., J.R.-O., C.-L.S., J.F.S., Z.S., and M.Z.; investigation, A.C., J.G., M.K., M.M., N. Liang, N. Liebe, C.-L.S., A.S., J.F.S., and M.Z.; resources, M.G., J.M.M., and J.R.P.; data curation, J.R.-O., C.-L.S., and J.F.S.; writing – original draft, J.R.-O., C.-L.S., and J.F.S.; writing – review & editing, E.A., N.H., J.R.-O., C.-L.S., J.F.S., and S.v.H. with input from all authors; visualization, J.R.-O., C.-L.S., and J.F.S.; supervision, N.H., M.G., P.M., S.v.H., and T.E.W.; project administration: N.H. and S.v.H.; funding acquisition, N.H. and S.v.H.

DECLARATION OF INTERESTS

The authors declare no competing interests.

INCLUSION AND DIVERSITY

We support inclusive, diverse, and equitable conduct of research.

Received: September 8, 2022
Revised: December 12, 2022
Accepted: January 25, 2023
Published: February 17, 2023

REFERENCES

1. Ingolia, N.T., Ghaemmaghami, S., Newman, J.R.S., and Weissman, J.S. (2009). Genome-wide analysis in vivo of translation with nucleotide resolution using ribosome profiling. Science *324*, 218–223.
2. Mudge, J.M., Ruiz-Orera, J., Prensner, J.R., Brunet, M.A., Calvet, F., Jungreis, I., Gonzalez, J.M., Magrane, M., Martinez, T.F., Schulz, J.F., et al. (2022). Standardized annotation of translated open reading frames. Nat. Biotechnol. *40*, 994–999.
3. Wright, B.W., Yi, Z., Weissman, J.S., and Chen, J. (2022). The dark proteome: translation from noncanonical open reading frames. Trends Cell Biol. *32*, 243–258.
4. Makarewich, C.A., and Olson, E.N. (2017). Mining for micropeptides. Trends Cell Biol. *27*, 685–696.
5. Prensner, J.R., Enache, O.M., Luria, V., Krug, K., Clauser, K.R., Dempster, J.M., Karger, A., Wang, L., Stumbraite, K., Wang, V.M., et al. (2021). Noncanonical open reading frames encode functional proteins essential for cancer cell survival. Nat. Biotechnol. *39*, 697–704.
6. Chen, J., Brunner, A.D., Cogan, J.Z., Nuñez, J.K., Fields, A.P., Adamson, B., Itzhak, D.N., Li, J.Y., Mann, M., Leonetti, M.D., et al. (2020). Pervasive functional translation of noncanonical human open reading frames. Science *367*, 1140–1146.
7. Vakirlis, N., Vance, Z., Duggan, K.M., and McLysaght, A. (2022). De novo birth of functional microproteins in the human lineage. Cell Rep. *41*, 111808.
8. An, N.A., Zhang, J., Mo, F., Luan, X., Tian, L., Shen, Q.S., Li, X., Li, C., Zhou, F., Zhang, B., et al. (2023). De novo genes with an lncRNA origin encode unique human brain developmental functionality. Nat. Ecol. Evol. https://doi.org/10.1038/s41559-022-01925-6.
9. Takita, J., Chen, Y., Okubo, J., Sanada, M., Adachi, M., Ohki, K., Nishimura, R., Hanada, R., Igarashi, T., Hayashi, Y., et al. (2011). Aberrations of NEGR1 on 1p31 and MYEOV on 11q13 in neuroblastoma. Cancer Sci. *102*, 1645–1650.
10. Suenaga, Y., Islam, S.M.R., Alagu, J., Kaneko, Y., Kato, M., Tanaka, Y., Kawana, H., Hossain, S., Matsumoto, D., Yamamoto, M., et al. (2014). NCYM, a Cis-antisense gene of MYCN, encodes a de novo evolved protein that inhibits GSK3β resulting in the stabilization of MYCN in human neuroblastomas. PLoS Genet. *10*, e1003996.
11. Buhl, A.M., Jurlander, J., Jørgensen, F.S., Ottesen, A.M., Cowland, J.B., Gjerdrum, L.M., Hansen, B.V., and Leffers, H. (2006). Identification of a gene on chromosome 12q22 uniquely overexpressed in chronic lymphocytic leukemia. Blood *107*, 2904–2911.
12. Kondo, T., Hashimoto, Y., Kato, K., Inagaki, S., Hayashi, S., and Kageyama, Y. (2007). Small peptide regulators of actin-based cell morphogenesis encoded by a polycistronic mRNA. Nat. Cell Biol. *9*, 660–665.
13. Galindo, M.I., Pueyo, J.I., Fouix, S., Bishop, S.A., and Couso, J.P. (2007). Peptides encoded by short ORFs control development and define a new eukaryotic gene family. PLoS Biol. *5*, e106.
14. Savard, J., Marques-Souza, H., Aranda, M., and Tautz, D. (2006). A segmentation gene in Tribolium produces a polycistronic mRNA that codes for multiple conserved peptides. Cell *126*, 559–569.

CellPress
OPEN ACCESS

15. Lee, C., Zeng, J., Drew, B.G., Sallam, T., Martin-Montalvo, A., Wan, J., Kim, S.J., Mehta, H., Hevener, A.L., de Cabo, R., et al. (2015). The mitochondrial-derived peptide MOTS-c promotes metabolic homeostasis and reduces obesity and insulin resistance. Cell Metab. *21*, 443–454.

16. Kastin, A. (2013). Handbook of Biologically Active Peptides (Academic Press).

17. Dittmar, G., Hernandez, D.P., Kowenz-Leutz, E., Kirchner, M., Kahlert, G., Wesolowski, R., Baum, K., Knoblich, M., Hofstätter, M., Muller, A., et al. (2019). PRISMA: protein interaction screen on peptide matrix reveals interaction footprints and modifications- dependent interactome of intrinsically disordered C/EBPβ. iScience *13*, 351–370.

18. Meyer, K., Kirchner, M., Uyar, B., Cheng, J.Y., Russo, G., Hernandez-Miranda, L.R., Szymborska, A., Zauber, H., Rudolph, I.M., Willnow, T.E., et al. (2018). Mutations in disordered regions can cause disease by creating dileucine motifs. Cell *175*, 239–253.e17.

19. Ramberger, E., Sapozhnikova, V., Kowenz-Leutz, E., Zimmermann, K., Nicot, N., Nazarov, P.V., Perez-Hernandez, D., Reimer, U., Mertins, P., Dittmar, G., et al. (2021). PRISMA and BioID disclose a motifs-based interactome of the intrinsically disordered transcription factor C/EBPα. iScience *24*, 102686.

20. Ramberger, E., Suarez-Artiles, L., Perez-Hernandez, D., Haji, M., Popp, O., Reimer, U., Leutz, A., Dittmar, G., and Mertins, P. (2021). A universal peptide matrix interactomics approach to disclose motif-dependent protein binding. Mol. Cell. Proteomics *20*, 100135.

21. Mackowiak, S.D., Zauber, H., Bielow, C., Thiel, D., Kutz, K., Calviello, L., Mastrobuoni, G., Rajewsky, N., Kempa, S., Selbach, M., et al. (2015). Extensive identification and analysis of conserved small ORFs in animals. Genome Biol. *16*, 179.

22. van Heesch, S., Witte, F., Schneider-Lunitz, V., Schulz, J.F., Adami, E., Faber, A.B., Kirchner, M., Maatz, H., Blachut, S., Sandmann, C.L., et al. (2019). The translational landscape of the human heart. Cell *178*, 242–260.e29.

23. Wang, Z.Y., Leushkin, E., Liechti, A., Ovchinnikova, S., Mößinger, K., Brüning, T., Rummel, C., Grützner, F., Cardoso-Moreira, M., Janich, P., et al. (2020). Transcriptome and translatome co-evolution in mammals. Nature *588*, 642–647.

24. Kustatscher, G., Collins, T., Gingras, A.C., Guo, T., Hermjakob, H., Ideker, T., Lilley, K.S., Lundberg, E., Marcotte, E.M., Ralser, M., et al. (2022). Understudied proteins: opportunities and challenges for functional proteomics. Nat. Methods *19*, 774–779.

25. Hao, Y., Zhang, L., Niu, Y., Cai, T., Luo, J., He, S., Zhang, B., Zhang, D., Qin, Y., Yang, F., et al. (2018). SmProt: a database of small proteins encoded by annotated coding and non-coding RNA loci. Brief. Bioinform. *19*, 636–643.

26. Choteau, S.A., Wagner, A., Pierre, P., Spinelli, L., and Brun, C. (2021). MetamORF: a repository of unique short open reading frames identified by both experimental and computational approaches for gene and metagene analyses. Database *2021*, baab032. https://doi.org/10.1093/database/baab032.

27. Olexiouk, V., Van Criekinge, W., and Menschaert, G. (2018). An update on sORFs.org: a repository of small ORFs identified by ribosome profiling. Nucleic Acids Res. *46*, D497–D502.

28. Zhang, S., Reljić, B., Liang, C., Kerouanton, B., Francisco, J.C., Peh, J.H., Mary, C., Jagannathan, N.S., Olexiouk, V., Tang, C., et al. (2020). Mitochondrial peptide BRAWNIN is essential for vertebrate respiratory complex III assembly. Nat. Commun. *11*, 1312.

29. Desiere, F., Deutsch, E.W., King, N.L., Nesvizhskii, A.I., Mallick, P., Eng, J., Chen, S., Eddes, J., Loevenich, S.N., and Aebersold, R. (2006). The PeptideAtlas project. Nucleic Acids Res. *34*, D655–D658.

30. Szklarczyk, D., Gable, A.L., Lyon, D., Junge, A., Wyder, S., Huerta-Cepas, J., Simonovic, M., Doncheva, N.T., Morris, J.H., Bork, P., et al. (2019). STRING v11: protein-protein association networks with increased coverage, supporting functional discovery in genome-wide experimental datasets. Nucleic Acids Res. *47*, D607–D613.

31. Rathore, A., Chu, Q., Tan, D., Martinez, T.F., Donaldson, C.J., Diedrich, J.K., Yates, J.R., and Saghatelian, A. (2018). MIEF1 microprotein regulates mitochondrial translation. Biochemistry *57*, 5564–5575.

32. Schulze, W.X., and Mann, M. (2004). A novel proteomic screen for peptide-protein interactions. J. Biol. Chem. *279*, 10756–10764.

33. Blomen, V.A., Májek, P., Jae, L.T., Bigenzahn, J.W., Nieuwenhuis, J., Staring, J., Sacco, R., van Diemen, F.R., Olk, N., Stukalov, A., et al. (2015). Gene essentiality and synthetic lethality in haploid human cells. Science *350*, 1092–1096.

34. Davey, N.E., Van Roey, K., Weatheritt, R.J., Toedt, G., Uyar, B., Altenberg, B., Budd, A., Diella, F., Dinkel, H., and Gibson, T.J. (2012). Attributes of short linear motifs. Mol. Biosyst. *8*, 268–281.

35. Erady, C., Boxall, A., Puntambekar, S., Suhas Jagannathan, N., Chauhan, R., Chong, D., Meena, N., Kulkarni, A., Kasabe, B., Prathivadi Bhayankaram, K., et al. (2021). Pan-cancer analysis of transcripts encoding novel open-reading frames (nORFs) and their potential biological functions. NPJ Genom. Med. *6*, 4.

36. Dosztányi, Z., Csizmok, V., Tompa, P., and Simon, I. (2005). IUPred: web server for the prediction of intrinsically unstructured regions of proteins based on estimated energy content. Bioinformatics *21*, 3433–3434. https://doi.org/10.1093/bioinformatics/bti541.

37. Gouw, M., Michael, S., Sámano-Sánchez, H., Kumar, M., Zeke, A., Lang, B., Bely, B., Chemes, L.B., Davey, N.E., Deng, Z., et al. (2018). The eukaryotic linear motif resource - 2018 update. Nucleic Acids Res. *46*, D428–D434.

38. Castello, A., Fischer, B., Eichelbaum, K., Horos, R., Beckmann, B.M., Strein, C., Davey, N.E., Humphreys, D.T., Preiss, T., Steinmetz, L.M., et al. (2012). Insights into RNA biology from an atlas of mammalian mRNA-binding proteins. Cell *149*, 1393–1406.

39. Bagchi, S., Fredriksson, R., and Wallén-Mackenzie, Å. (2015). In situ proximity ligation assay (PLA). Methods Mol. Biol. *1318*, 149–159.

40. Lafer, E.M. (2002). Clathrin-protein interactions. Traffic *3*, 513–520.

41. Kumar, M., Michael, S., Alvarado-Valverde, J., Mészáros, B., Sámano-Sánchez, H., Zeke, A., Dobson, L., Lazar, T., Örd, M., Nagpal, A., et al. (2022). The Eukaryotic Linear Motif resource: 2022 release. Nucleic Acids Res. *50*, D497–D508.

42. Collinet, C., Stöter, M., Bradshaw, C.R., Samusik, N., Rink, J.C., Kenski, D., Habermann, B., Buchholz, F., Henschel, R., Mueller, M.S., et al. (2010). Systems survey of endocytosis by multiparametric image analysis. Nature *464*, 243–249. https://doi.org/10.1038/nature08779.

43. Sorkin, A., and von Zastrow, M. (2009). Endocytosis and signalling: intertwining molecular networks. Nat. Rev. Mol. Cell Biol. *10*, 609–622. https://doi.org/10.1038/nrm2748.

44. Rainero, E. (2016). Extracellular matrix endocytosis in controlling matrix turnover and beyond: emerging roles in cancer. Biochem. Soc. Trans. *44*, 1347–1354.

45. Calviello, L., Mukherjee, N., Wyler, E., Zauber, H., Hirsekorn, A., Selbach, M., Landthaler, M., Obermayer, B., and Ohler, U. (2016). Detecting actively translated open reading frames in ribosome profiling data. Nat. Methods *13*, 165–170.

46. Calviello, L., Hirsekorn, A., and Ohler, U. (2020). Quantification of translation uncovers the functions of the alternative transcriptome. Nat. Struct. Mol. Biol. *27*, 717–725.

47. Erhard, F., Halenius, A., Zimmermann, C., L'Hernault, A., Kowalewski, D.J., Weekes, M.P., Stevanovic, S., Zimmer, R., and Dölken, L. (2018). Improved Ribo-seq enables identification of cryptic translation events. Nat. Methods *15*, 363–366.

48. Clauwaert, J., McVey, Z., Gupta, R., and Menschaert, G. (2021). TIS Transformer: re-annotation of the human proteome using deep learning. https://doi.org/10.1101/2021.11.18.468957.

49. Ladoukakis, E., Pereira, V., Magny, E.G., Eyre-Walker, A., and Couso, J.P. (2011). Hundreds of putatively functional small open reading frames in Drosophila. Genome Biol. *12*, R118.

50. Witte, F., Ruiz-Orera, J., Mattioli, C.C., Blachut, S., Adami, E., Schulz, J.F., Schneider-Lunitz, V., Hummel, O., Patone, G., Mücke, M.B., et al. (2021). A trans locus causes a ribosomopathy in hypertrophic hearts that affects mRNA translation in a protein length-dependent fashion. Genome Biol. *22*, 191.

51. Kim, M.S., Pinto, S.M., Getnet, D., Nirujogi, R.S., Manda, S.S., Chaerkady, R., Madugundu, A.K., Kelkar, D.S., Isserlin, R., Jain, S., et al. (2014). A draft map of the human proteome. Nature *509*, 575–581.

52. Mills, R.J., Titmarsh, D.M., Koenig, X., Parker, B.L., Ryall, J.G., Quaife-Ryan, G.A., Voges, H.K., Hodson, M.P., Ferguson, C., Drowley, L., et al. (2017). Functional screening in human cardiac organoids reveals a metabolic mechanism for cardiomyocyte cell cycle arrest. Proc. Natl. Acad. Sci. USA *114*, E8372–E8381.

53. Chothani, S.P., Adami, E., Widjaja, A.A., Langley, S.R., Viswanathan, S., Pua, C.J., Zhihao, N.T., Harmston, N., D'Agostino, G., Whiffin, N., et al. (2022). A high-resolution map of human RNA translation. Mol. Cell *82*, 2885–2899.e8.

54. Chong, C., Müller, M., Pak, H., Harnett, D., Huber, F., Grun, D., Leleu, M., Auger, A., Arnaud, M., Stevenson, B.J., et al. (2020). Integrated proteogenomic deep sequencing and analytics accurately identify non-canonical peptides in tumor immunopeptidomes. Nat. Commun. *11*, 1293.

55. Racle, J., Michaux, J., Rockinger, G.A., Arnaud, M., Bobisse, S., Chong, C., Guillaume, P., Coukos, G., Harari, A., Jandus, C., et al. (2019). Robust prediction of HLA class II epitopes by deep motif deconvolution of immunopeptidomes. Nat. Biotechnol. *37*, 1283–1286.

56. Cox, J., and Mann, M. (2008). MaxQuant enables high peptide identification rates, individualized p.p.b.-range mass accuracies and proteome-wide protein quantification. Nat. Biotechnol. *26*, 1367–1372.

57. Ma, J., Saghatelian, A., and Shokhirev, M.N. (2018). The influence of transcript assembly on the proteogenomics discovery of microproteins. PLoS One *13*, e0194518.

58. Deutsch, E.W., Mendoza, L., Shteynberg, D., Slagel, J., Sun, Z., and Moritz, R.L. (2015). Trans-proteomic Pipeline, a standardized data processing pipeline for large-scale reproducible proteomics informatics. Proteomics Clin. Appl. *9*, 745–754. https://doi.org/10.1002/prca.201400164.

59. Ortega, B., Mason, A.K., and Welling, P.A. (2012). A tandem Di-hydrophobic motif mediates clathrin-dependent endocytosis via direct binding to the AP-2 $\alpha\sigma2$ subunits. J. Biol. Chem. *287*, 26867–26875.

60. Ruiz-Orera, J., and Albà, M.M. (2019). Translation of Small Open Reading Frames: Roles in Regulation and Evolutionary Innovation. Trends Genet. *35*, 186–198.

61. Lu, S., Zhang, J., Lian, X., Sun, L., Meng, K., Chen, Y., Sun, Z., Yin, X., Li, Y., Zhao, J., et al. (2019). A hidden human proteome encoded by "non-coding" genes. Nucleic Acids Res. *47*, 8111–8125.

62. McLysaght, A., and Hurst, L.D. (2016). Open questions in the study of de novo genes: what, how and why. Nat. Rev. Genet. *17*, 567–578.

63. Tautz, D. (2009). Polycistronic peptide coding genes in eukaryotes–how widespread are they? Brief. Funct. Genomic. Proteomic. *8*, 68–74.

64. Ruiz-Orera, J., and Albà, M.M. (2019). Conserved regions in long non-coding RNAs contain abundant translation and protein–RNA interaction signatures. NAR Genom. Bioinform. *1*, e2. https://doi.org/10.1093/nargab/lqz002.

65. Carvunis, A.R., Rolland, T., Wapinski, I., Calderwood, M.A., Yildirim, M.A., Simonis, N., Charloteaux, B., Hidalgo, C.A., Barbette, J., Santhanam, B., et al. (2012). Proto-genes and de novo gene birth. Nature *487*, 370–374.

66. Vakirlis, N., Carvunis, A.R., and McLysaght, A. (2020). Synteny-based analyses indicate that sequence divergence is not the main source of orphan genes. eLife *9*, e53500. https://doi.org/10.7554/eLife.53500.

67. Van Oss, S.B., and Carvunis, A.R. (2019). De novo gene birth. PLOS Genet. *15*, e1008160.

68. Tautz, D., and Domazet-Lošo, T. (2011). The evolutionary origin of orphan genes. Nat. Rev. Genet. *12*, 692–702.

69. Xie, C., Bekpen, C., Künzel, S., Keshavarz, M., Krebs-Wheaton, R., Skrabar, N., Ullrich, K.K., and Tautz, D. (2019). A de novo evolved gene in the house mouse regulates female pregnancy cycles. 10.7554. eLife *44392*.

70. Neme, R., Amador, C., Yildirim, B., McConnell, E., and Tautz, D. (2017). Random sequences are an abundant source of bioactive RNAs or peptides. Nat. Ecol. Evol. *1*, 0217.

71. Schleich, S., Strassburger, K., Janiesch, P.C., Koledachkina, T., Miller, K.K., Haneke, K., Cheng, Y.S., Kuechler, K., Stoecklin, G., Duncan, K.E., et al. (2014). DENR-MCT-1 promotes translation re-initiation downstream of uORFs to control tissue growth. Nature *512*, 208–212.

72. Dever, T.E., Ivanov, I.P., and Sachs, M.S. (2020). Conserved upstream open reading frame nascent peptides that control translation. Annu. Rev. Genet. *54*, 237–264. https://doi.org/10.1146/annurev-genet-112618-043822.

73. Yabe, D., Brown, M.S., and Goldstein, J.L. (2002). Insig-2, a second endoplasmic reticulum protein that binds SCAP and blocks export of sterol regulatory element-binding proteins. Proc. Natl. Acad. Sci. USA *99*, 12753–12758.

74. Muttenthaler, M., King, G.F., Adams, D.J., and Alewood, P.F. (2021). Trends in peptide drug discovery. Nat. Rev. Drug Discov. *20*, 309–325.

75. Kavčič, B., Tkačik, G., and Bollenbach, T. (2020). Mechanisms of drug interactions between translation-inhibiting antibiotics. Nat. Commun. *11*, 4013.

76. Perez-Riverol, Y., Csordas, A., Bai, J., Bernal-Llinares, M., Hewapathirana, S., Kundu, D.J., Inuganti, A., Griss, J., Mayer, G., Eisenacher, M., et al. (2019). The PRIDE database and related tools and resources in 2019: improving support for quantification data. Nucleic Acids Res. *47*, D442–D450.

77. Schueler, M., Munschauer, M., Gregersen, L.H., Finzel, A., Loewer, A., Chen, W., Landthaler, M., and Dieterich, C. (2014). Differential protein occupancy profiling of the mRNA transcriptome. Genome Biol. *15*, R15.

78. Sandmann, C.-L., Schulz, J., Ruiz-Orera, J., Kirchner, M., Ziehm, M., Adami, E., Marczenke, M., Christ, A., Liebe, N., Greiner, J., et al. (2023). Evolutionary origins and interactomes of human young microproteins and small peptides translated from short open reading frames. Mendeley Data, V1. https://doi.org/10.17632/CKGDGTY885.1.

79. Altschul, S.F. (2005). BLAST algorithm. eLS. https://doi.org/10.1038/npg.els.0005253.

80. Lin, M.F., Jungreis, I., and Kellis, M. (2011). PhyloCSF: a comparative genomics method to distinguish protein coding and non-coding regions. Bioinformatics *27*, i275–i282.

81. Löytynoja, A. (2021). Phylogeny-aware alignment with PRANK and PAGAN. Methods Mol. Biol. *2231*, 17–37.

82. Dobin, A., Davis, C.A., Schlesinger, F., Drenkow, J., Zaleski, C., Jha, S., Batut, P., Chaisson, M., and Gingeras, T.R. (2013). STAR: ultrafast universal RNA-seq aligner. Bioinformatics *29*, 15–21.

83. Quinlan, A.R. (2014). BEDTools: the Swiss-army tool for genome feature analysis. Curr. Protoc. Bioinformatics *47*, 11.12.1–11.1234.

84. Lee, B.T., Barber, G.P., Benet-Pagès, A., Casper, J., Clawson, H., Diekhans, M., Fischer, C., Gonzalez, J.N., Hinrichs, A.S., Lee, C.M., et al. (2022). The UCSC Genome Browser database: 2022 update. Nucleic Acids Res. *50*, D1115–D1122. https://doi.org/10.1093/nar/gkab959.

85. Pertea, M., Pertea, G.M., Antonescu, C.M., Chang, T.C., Mendell, J.T., and Salzberg, S.L. (2015). StringTie enables improved reconstruction of a transcriptome from RNA-seq reads. Nat. Biotechnol. *33*, 290–295. https://doi.org/10.1038/nbt.3122.

CellPress
OPEN ACCESS

86. Smit, A., and Hubley, R. (2008–2015). RepeatModeler Open-1.0. RepeatModeler Open-1.0. http://www.repeatmasker.org.
87. R Core Team (2019). R: A Language and Environment for Statistical Computing (R Foundation for Statistical Computing). https://www.R-project.org/.
88. Reimand, J., Arak, T., Adler, P., Kolberg, L., Reisberg, S., Peterson, H., and Vilo, J. (2016). g:profiler-a web server for functional interpretation of gene lists (2016 update). Nucleic Acids Res. *44*, W83–W89.
89. Love, M.I., Huber, W., and Anders, S. (2014). Moderated estimation of fold change and dispersion for RNA-seq data with DESeq2. Genome Biol. *15*, 550.
90. Calviello, L., Sydow, D., Harnett, D., and Ohler, U. (2019). Ribo-seQC: comprehensive analysis of cytoplasmic and organellar ribosome profiling data. Preprint at bioRxiv. https://doi.org/10.1101/601468.
91. Kong, A.T., Leprevost, F.V., Avtonomov, D.M., Mellacheruvu, D., and Nesvizhskii, A.I. (2017). MSFragger: ultrafast and comprehensive peptide identification in mass spectrometry-based proteomics. Nat. Methods *14*, 513–520.
92. Mendoza, L., Deutsch, E.W., Sun, Z., Campbell, D.S., Shteynberg, D.D., and Moritz, R.L. (2018). Flexible and fast mapping of peptides to a proteome with ProteoMapper. J. Proteome Res. *17*, 4337–4344. https://doi.org/10.1021/acs.jproteome.8b00544.
93. MacLean, B., Tomazela, D.M., Shulman, N., Chambers, M., Finney, G.L., Frewen, B., Kern, R., Tabb, D.L., Liebler, D.C., and MacCoss, M.J. (2010). Skyline: an open source document editor for creating and analyzing targeted proteomics experiments. Bioinformatics *26*, 966–968.
94. Schindelin, J., Arganda-Carreras, I., Frise, E., Kaynig, V., Longair, M., Pietzsch, T., Preibisch, S., Rueden, C., Saalfeld, S., Schmid, B., et al. (2012). Fiji: an open-source platform for biological-image analysis. Nat. Methods *9*, 676–682.
95. Reynisson, B., Alvarez, B., Paul, S., Peters, B., and Nielsen, M. (2020). NetMHCpan-4.1 and NetMHCIIpan-4.0: improved predictions of MHC antigen presentation by concurrent motif deconvolution and integration of MS MHC eluted ligand data. Nucleic Acids Res. *48*, W449–W454.
96. Sandmann, C.-L., Schulz, J.F., Ruiz-Orera, J., Kirchner, M., Ziehm, M., Adami, E., Marczenke, M., Christ, A., Liebe, N., Greiner, J., et al. (2023). Evolutionary origins and interactomes of human young microproteins and small peptides translated from short open reading frames. https://doi.org/10.5281/zenodo.7553817.
97. Howe, K.L., Achuthan, P., Allen, J., Allen, J., Alvarez-Jarreta, J., Amode, M.R., Armean, I.M., Azov, A.G., Bennett, R., Bhai, J., et al. (2021). Ensembl 2021. Nucleic Acids Res. *49*, D884–D891.
98. Hecker, N., and Hiller, M. (2020). A genome alignment of 120 mammals highlights ultraconserved element variability and placenta-associated enhancers. GigaScience *9*, giz159. https://doi.org/10.1093/gigascience/giz159.
99. Weisman, C.M., Murray, A.W., and Eddy, S.R. (2020). Many, but not all, lineage-specific genes can be explained by homology detection failure. PLoS Biol. *18*, e3000862. https://doi.org/10.1371/journal.pbio.3000862.
100. Mason, V.C., Li, G., Minx, P., Schmitz, J., Churakov, G., Doronina, L., Melin, A.D., Dominy, N.J., Lim, N.T.-L., Springer, M.S., et al. (2016). Genomic analysis reveals hidden biodiversity within colugos, the sister group to primates. Sci. Adv. *2*, e1600633.
101. Vakirlis, N., and McLysaght, A. (2019). Computational prediction of de novo emerged protein-coding genes. Methods Mol. Biol. *1851*, 63–81.
102. Patraquim, P., Mumtaz, M.A.S., Pueyo, J.I., Aspden, J.L., and Couso, J.P. (2020). Developmental regulation of canonical and small ORF translation from mRNAs. Genome Biol. *21*, 128.
103. Ruiz-Orera, J., Verdaguer-Grau, P., Villanueva-Cañas, J.L., Messeguer, X., and Albà, M.M. (2018). Translation of neutrally evolving peptides provides a basis for de novo gene evolution. Nat. Ecol. Evol. *2*, 890–896.
104. Ruiz-Orera, J., Hernandez-Rodriguez, J., Chiva, C., Sabidó, E., Kondova, I., Bontrop, R., Marqués-Bonet, T., and Albà, M.M. (2015). Origins of de novo genes in human and chimpanzee. PLoS Genet. *11*, e1005721.
105. Friesen, M., Warren, C.R., Yu, H., Toyohara, T., Ding, Q., Florido, M.H.C., Sayre, C., Pope, B.D., Goff, L.A., Rinn, J.L., et al. (2020). Mitoregulin controls β-oxidation in human and mouse adipocytes. Stem Cell Rep. *14*, 590–602.
106. Chugunova, A., Loseva, E., Mazin, P., Mitina, A., Navalayeu, T., Bilan, D., Vishnyakova, P., Marey, M., Golovina, A., Serebryakova, M., et al. (2019). LINC00116 codes for a mitochondrial peptide linking respiration and lipid metabolism. Proc. Natl. Acad. Sci. USA *116*, 4940–4945.
107. Zhou, B., Ji, B., Liu, K., Hu, G., Wang, F., Chen, Q., Yu, R., Huang, P., Ren, J., Guo, C., et al. (2021). EVLncRNAs 2.0: an updated database of manually curated functional long non-coding RNAs validated by low-throughput experiments. Nucleic Acids Res. *49*, D86–D91.
108. Schneider, C.A., Rasband, W.S., and Eliceiri, K.W. (2012). NIH Image to ImageJ: 25 years of image analysis. Nat. Methods *9*, 671–675.
109. Rappsilber, J., Ishihama, Y., and Mann, M. (2003). Stop and go extraction tips for matrix-assisted laser desorption/ionization, nanoelectrospray, and LC/MS sample pretreatment in proteomics. Anal. Chem. *75*, 663–670.
110. Ritchie, M.E., Phipson, B., Wu, D., Hu, Y., Law, C.W., Shi, W., and Smyth, G.K. (2015). limma powers differential expression analyses for RNA-sequencing and microarray studies. Nucleic Acids Res. *43*, e47.
111. Keilhauer, E.C., Hein, M.Y., and Mann, M. (2015). Accurate protein complex retrieval by affinity enrichment mass spectrometry (AE-MS) rather than affinity purification mass spectrometry (AP-MS). Mol. Cell. Proteomics *14*, 120–135.
112. Shao, Y., Chen, C., Shen, H., He, B.Z., Yu, D., Jiang, S., Zhao, S., Gao, Z., Zhu, Z., Chen, X., et al. (2019). GenTree, an integrated resource for analyzing the evolution and function of primate-specific coding genes. Genome Res. *29*, 682–696.
113. The Gene Ontology Consortium; the Gene Ontology Consortium (2017). Expansion of the Gene Ontology KnowledgeBase and resources. Nucleic Acids Res. *45*, D331–D338. https://doi.org/10.1093/nar/gkw1108.
114. Couso, J.P. (2015). Finding smORFs: getting closer. Genome Biol. *16*, 189.
115. Vizcaíno, J.A., Deutsch, E.W., Wang, R., Csordas, A., Reisinger, F., Ríos, D., Dianes, J.A., Sun, Z., Farrah, T., Bandeira, N., et al. (2014). ProteomeXchange provides globally coordinated proteomics data submission and dissemination. Nat. Biotechnol. *32*, 223–226.
116. Deutsch, E.W., Sun, Z., Campbell, D.S., Binz, P.A., Farrah, T., Shteynberg, D., Mendoza, L., Omenn, G.S., and Moritz, R.L. (2016). Tiered human integrated sequence search databases for shotgun proteomics. J. Proteome Res. *15*, 4091–4100. https://doi.org/10.1021/acs.jproteome.6b00445.
117. Krokhin, O.V., Craig, R., Spicer, V., Ens, W., Standing, K.G., Beavis, R.C., and Wilkins, J.A. (2004). An improved model for prediction of retention times of tryptic peptides in ion pair reversed-phase HPLC: its application to protein peptide mapping by off-line HPLC-MALDI MS. Mol. Cell. Proteomics *3*, 908–919.
118. Giurgiu, M., Reinhard, J., Brauner, B., Dunger-Kaltenbach, I., Fobo, G., Frishman, G., Montrone, C., and Ruepp, A. (2019). Corum: the comprehensive resource of mammalian protein complexes-2019. Nucleic Acids Res. *47*, D559–D563.
119. Christensen, E.I., and Birn, H. (2002). Megalin and cubilin: multifunctional endocytic receptors. Nat. Rev. Mol. Cell Biol. *3*, 256–266.
120. Madani, F., Lindberg, S., Langel, U., Futaki, S., and Gräslund, A. (2011). Mechanisms of cellular uptake of cell-penetrating peptides. J. Biophys. *2011*, 414729.
121. Ruseska, I., and Zimmer, A. (2020). Internalization mechanisms of cell-penetrating peptides. Beilstein J. Nanotechnol. *11*, 101–123.

STAR★METHODS

KEY RESOURCES TABLE

REAGENT or RESOURCE	SOURCE	IDENTIFIER
**Antibodies**		
Mouse monoclonal anti-FLAG (M2), 1:500	Sigma-Aldrich	Cat#F1804; RRID: AB_262044
Rabbit monoclonal anti-ATPIF1 (D6P1Q), 1:1000	Cell Signaling Technology	Cat#13268; RRID: AB_10949890
Rabbit polyclonal anti-Clathrin Heavy Chain (P1663), 1:100	Cell Signaling Technology	Cat#2410; RRID: AB_2083156
Alexa Fluor 488 anti-rabbit, 1:500	Invitrogen	Cat#A11070; RRID: AB_142134
Alexa Fluor 594 anti-mouse, 1:500	Invitrogen	Cat#A11005; RRID: AB_141372
Rabbit monoclonal anti-V5-Tag (D3H8Q), 1:500	Cell Signaling Technology	Cat#13202; RRID: AB_2687461
Chicken polyclonal anti-BirA, 1:500	BioFront Technologies	Cat#BID-CP-100, RRID: not available
Alexa Fluor 488 anti-mouse, 1:500	Invitrogen	Cat#A11001, RRID: AB_2534069
Alexa Fluor 488 anti-chicken, 1:500	Invitrogen	Cat#A11039, RRID: AB_142924
Alexa Fluor 594 anti-rabbit, 1:500	Invitrogen	Cat#A-11037, RRID: AB_2534095
Mouse monoclonal anti-EEA1, 1:100	BD Transduction Laboratories	Cat#610457, RRID: AB_397830
Alexa Fluor 555 anti-mouse, 1:500	Invitrogen	Cat#A31570, RRID: AB_2536180
**Biological samples**		
human heart tissue of five adult cardiomyopathy patients:<br>Sample 1: female, DCM, age range 55-60<br>Sample 2: male, DCM, age range 30-35<br>Sample 3: male, HCM, age range 40-45<br>Sample 4: male, HCM, age range 55-60<br>Sample 5: female, HCM, age range 50-55	Harvard Medical School, Boston USA, previously used in van Heesch et al.[22]	N/A
**Chemicals, peptides, and recombinant proteins**		
Alexa 647-conjugated transferrin	Invitrogen	Cat#T23366
Lysyl Endopeptidase	Wako	Cat#125-05061
Trypsin Gold	Promega	Cat#V5280
PRISMA synthetic peptides on a cellulose membrane	JPT	PRISMA - peptides
Crude synthetic peptides for PRM assay	JPT	SpikeTides/Maxi SpikeTides
Proteinase K	Sigma-Aldrich	Cat#3115879001
Poly-D-Lysine	Sigma-Aldrich	Cat#P0899-50MG
cOmplete, EDTA-free Protease Inhibitor Cocktail	Roche	Cat#11873580001
synthetic peptides with TAT-sequence	Pepscan	custom
DAPI	Thermo Fisher	Cat#R37606
**Critical commercial assays**		
Duolink® In Situ Proximity Ligation Assay Starter Kit, Red, Mouse/Rabbit	Sigma-Aldrich	Cat#DUO92101-1KT
Alexa Fluor 594 Protein Labeling Kit	Thermo Fisher	Cat#A10239
Dual Glo® Luciferase Assay System	Promega	Cat#E2920
**Deposited data**		
Cataloged set of 7,264 Ribo-seq ORFs	Mudge et al.[2]	https://www.gencodegenes.org/pages/riboseq_orfs/
MS data: PRISMA (microproteins >15aa)	This paper	ProteomeXchange (via PRIDE[76]): PXD033629, PXD033630
MS data: microprotein pull-down with phosphosite identification	This paper	ProteomeXchange (via PRIDE[76]): PXD033631

(*Continued on next page*)

Continued

REAGENT or RESOURCE	SOURCE	IDENTIFIER
MS data: PRISMA (peptides$_{3\text{-}15\ aa}$)	This paper	ProteomeXchange (via PRIDE[76]): PXD033651
MS data: PRM	This paper	ProteomeXchange (via PRIDE[76]): PXD036997
RNA-seq data for LINC01128 knockout and wild type cell lines	This paper	European Nucleotide Archive (ENA): PRJEB57619
Public human ribosome profiling data used for detection of sORFs$_{3\text{-}15\ aa}$	van Heesch et al.[22] and Wang et al.[23]	left ventricular heart tissue: EGA accession code EGAS00001003263[22]; kidney: EGA accession code EGAS00001003263[22]; liver: EGA accession code EGAS00001003263[22] and ArrayExpress accession code E-MTAB-7247[23]; brain: ArrayExpress accession code E-MTAB-7247[23]; testis: ArrayExpress accession code E-MTAB-7247[23]
Ribosome profiling data of mouse and rat tissues	van Heesch et al.,[22] Wang et al.,[23] and Witte et al.[50]	mouse heart: ENA accession code PRJEB29208[22]; mouse liver: ArrayExpress accession code E-MTAB-7247[23]; mouse brain: ArrayExpress accession code E-MTAB-7247[23]; mouse testis: ArrayExpress accession code E-MTAB-7247[23]; rat heart: ENA accession code PRJEB38096[50] and rat liver: ENA accession code PRJEB38096[50]
RNA sequencing datasets of HEK293T cells	Schueler et al.[77]	NCBI Sequence Read Archive (SRA) SRR1107836 and SRR1107837
Microscopy data: original images from immunofluorescence stainings	This paper	Mendeley Data https://doi.org/10.17632/ckgdgty885.1[78]
Supplemental excel tables Tables S1, S2, S3, S4, and S5	This paper	Mendeley Data https://doi.org/10.17632/ckgdgty885.1[78]
**Experimental models: Cell lines**		
Brown Norway rat yolk sac carcinoma (BN16) cells	ATCC	ATCC® CRL-2180
HeLa cells	kindly provided by A. Woehler, MDC	N/A
HeLa LINC01128-MP KO cells and wild type cells	Synthego Inc.	N/A
HEK293T/17 cells	ATCC	ATCC® CRL-11268
K562 cells	kindly provided by T. Blankenstein, MDC	N/A
**Software and algorithms**		
BLASTp (v.2.7.1)	Altschul[79]	https://blast.ncbi.nlm.nih.gov/Blast.cgi
PhyloCSF	Lin et al.[80]	https://github.com/mlin/PhyloCSF/wiki
PRANK (v.170427)	Löytynoja[81]	http://wasabiapp.org/software/prank/
STAR (v.2.5.2b)	Dobin et al.[82]	https://github.com/alexdobin/STAR
BEDTools (v.2.27.1)	Quinlan[83]	https://bedtools.readthedocs.io/en/latest/
UCSC Liftover	Lee et al.[84]	https://genome.ucsc.edu/goldenPath/help/hgTracksHelp.html#Liftover
Stringtie (v.1.2.1)	Pertea et al.[85]	https://ccb.jhu.edu/software/stringtie/
RepeatMasker (v.4.1.0)	Smit and Hubley[86]	https://www.repeatmasker.org/RepeatMasker/
MaxQuant (v.1.5.2.8. and v.1.6.0.1)	Cox and Mann[56]	https://www.maxquant.org/
R (v.3.6.1)	R Core Team[87]	https://www.r-project.org/
IUPred (v.1.0)	Dosztányi et al.[36]	https://iupred2a.elte.hu/
'elm_classes.tsv' file (version 1.4; 15 January 2018)	Gouw et al.[37]	http://elm.eu.org/

(*Continued on next page*)

Continued		
REAGENT or RESOURCE	SOURCE	IDENTIFIER
gProfiler2 (v0.2.0)	Reimand et al.[88]	https://cran.r-project.org/web/packages/gprofiler2/index.html
DESeq2 (v1.26.0)	Love et al.[89]	https://bioconductor.org/packages/release/bioc/html/DESeq2.html
RiboseQC	Calviello et al.[90]	https://github.com/ohlerlab/RiboseQC
PRICE (v1.0.3b)	Erhard et al.[47]	https://github.com/erhard-lab/price
TIS Transformer	Clauwaert et al.[48]	https://github.com/jdcla/TIS_transformer
MSFragger	Kong et al.[91]	https://msfragger.nesvilab.org/
Trans-Proteomic Pipeline	Deutsch et al.[58]	http://www.tppms.org/
Proteomapper (v.1.5)	Mendoza et al.[92]	http://www.tppms.org/tools/pm/
Skyline (v21.02)	MacLean et al.[93]	https://skyline.ms/project/home/software/Skyline/begin.view
Fiji	Schindelin et al.[94]	https://imagej.net/software/fiji/
NetMHCpan-4.1	Reynisson et al.[95]	https://services.healthtech.dtu.dk/service.php?NetMHCpan-4.1
ORFquant (v1.00)	Calviello et al.[46]	https://github.com/lcalviell/ORFquant
Other		
Python, R and Bash scripts used for the analysis	This paper	Zenodo https://doi.org/10.5281/zenodo.7553817[96]<br>Github: https://github.com/jorruior/riboseq_orfs_analyses

RESOURCE AVAILABILITY

Lead contact

Further information and requests for resources and reagents should be directed to and will be fulfilled by the lead contact, Norbert Hubner (nhuebner@mdc-berlin.de).

Materials availability

This study did not generate new unique reagents.

Data and code availability

- All MS and RNA-seq data created in this study have been deposited online and are publicly available as of the date of publication. Microscopy data reported in this paper have been deposited to Mendeley Data and are publicly available as of the date of publication. The DOI is listed in the key resources table. This paper also analyzes existing, publicly available data. These accession numbers for the datasets are listed in the key resources table.
- All original code has been deposited at GitHub as well as Zenodo and is publicly available as of the date of publication. The DOI is listed in the key resources table.
- Any additional information required to reanalyze the data reported in this paper is available from the lead contact upon request.

EXPERIMENTAL MODEL AND SUBJECT DETAILS

Cell culture

Human female HeLa cells (kindly provided by A. Woehler, MDC), HeLa LINC01128-MP KO cells (Synthego Inc., Redwood City, CA) and human female HEK293T/17 cells (CRL-11268, ATCC) were cultured in a humidified incubator at 37°C with 5% CO_2 using Dulbecco's modified eagle medium (DMEM) with high glucose (4.5 g/l), 10% fetal bovine serum (FBS), 2 mM L-glutamine and 1 mM sodium pyruvate. Brown Norway rat yolk sac carcinoma (BN16) cells (CRL-2180, ATCC, sex unknown) were cultivated in DMEM supplemented with 10% FBS and 1% Penicillin/Streptomycin. Human female K562 cells (kindly provided by T. Blankenstein, MDC) were cultured in RPMI medium supplemented with 10% FBS, 1% Penicillin/Streptomycin, 1 mM sodium pyruvate, 1 mM non-essential amino acids and 0.05 mM beta-mercaptoethanol. The medium was renewed every two to three days and cells were passaged at 80-90% confluency using standard trypsinization methods. Since K562 cells grow in suspension they were passaged without trypsin treatment.

Human primary material

Targeted proteomics (PRM) experiments for *in vivo* detection of microproteins and peptides$_{3\text{-}15\ \text{aa}}$ were performed on human heart tissue of adult cardiomyopathy patients with HCM (n = 3; mutations in MYH7 (2x) and MYBPC3) and DCM (n = 2; mutations in LMNA (2x)) obtained from Harvard Medical School, Boston USA, approved by the Mass General Brigham Human Research Protection Committee (Protocol 1999P010895); Harvard Longwood Campus Institutional Review Board (Protocol M11135). Samples were previously used in a study by van Heesch et al.[22]

METHOD DETAILS

Retrieval of sORF and control sequences

We retrieved a set of 7,264 Ribo-seq ORFs longer than 15 aa (denoted sORFs) annotated as part of the Phase I GENCODE ORF annotation project (Table S1).[2] For our analyses, we combined sORFs annotated as downstream overlapping ORFs (doORFs) and downstream ORFs (dORFs), since doORFs are a rare category that represents only 0.8% of all sORFs. This resulted in five considered sORF biotypes: lncRNA ORFs (lncORFs, encoded by presumed long non-coding RNAs and processed transcripts), upstream ORFs (uORFs, encoded by 5′ UTR sequences), upstream overlapping ORFs (uoORFs, encoded by 5′ UTR sequences and partially overlapping an annotated CDS in an alternative frame), internal ORFs (intORFs, fully overlapping an annotated CDS in an alternative frame), and downstream ORFs (dORFs, encoded by 3′ UTR sequences).

In order to determine the significance of our findings in subsequent evolutionary analyses, we defined two additional control sets. Firstly, we selected a set of 527 annotated CDS sequences (Ensembl v.101[97]) from genes in which all annotated protein isoforms were shorter than 100 amino acids (aa), selecting the longest CDS per gene and discarding incomplete CDSs without annotated start and/or stop codons (sCDS, Table S1). Secondly, we generated negative controls of untranslated ORF sequences. To this end we sampled length-matched sequences starting with ATG codons from non-coding regions of genes hosting uORFs and lncORFs and we translated them *in silico*. These untranslated regions did not overlap any annotated CDS or translated sORF sequence included in this study. We excluded genes hosting ORFs overlapping with conserved CDS sequences (uoORF, intORF, dORF), since the overlapping coding sequences can impose additional constraints on these ORFs. We generated a set of 2,068 and 2,914 length-matched untranslated regions in lncRNAs and 5′ UTRs, respectively. This set covers 93.54% of the genes containing uORFs and lncORFs included in the analysis. For the rest of the cases, we could not extract any compatible sequence from the corresponding untranslated regions.

Whole-genome alignments across mammalian species

We downloaded a comparative genomics resource that comprises pre-built whole-genome nucleotide alignments across 120 mammalian species[98] to calculate the extent of conservation of all human ORFs across the mammalian lineage. Additionally, we downloaded Cons30 multiple alignments from UCSC[84] comprising 27 different primate species (non-primate species were removed from the alignment). For a set of ten species with high-quality genomes (rhesus macaque, mouse, cow, dog, horse, elephant, opossum, chicken, western clawed frog, zebrafish) we also retrieved whole-genome Liftover chains from UCSC.[84] We included chicken, western clawed frog, and zebrafish as evolutionary outgroups to find potential ORFs with vertebrate conservation that extended beyond the mammalian clade. Finally, for every ORF we designed a custom script to define local multiple alignments including the species where the region could be fully aligned, discarding partial or ambiguous alignments.

Protein sequence conservation of sORFs and sCDS

Standard homology-detection approaches are not adequate for discovering sORF homologues in full transcriptomes or genomes due to the extense search space and the short length of the query sequences.[49,99] Hence, we instead estimated the levels of protein similarity of sORF- and sCDS-encoded microproteins (>15 aa), as well as untranslated ORF controls, to evaluate the significance of the similarity across a reduced set of previously aligned counterpart regions in mammals extracted from whole-genome alignments. When at least one genome region was aligned to an ORF, we ran BLASTp (v.2.7.1)[79] against species-specific databases containing all aligned regions and extracted the E-value of the alignment of the ORF against each specific counterpart region. Next, we calculated a conservation score (CS) for each encoded microprotein, defined as the negative logarithmic value ($-\log_{10}$) of the median E-values across all aligned species. Since each species-specific database contained variable numbers of counterpart regions due to differences in genome quality and divergence, we decided to use E-values instead of bit-scores as they are adjusted by the size of the sequence databases. However, pairwise alignment bit-scores computed by BLAST can also clearly distinguish between young and conserved sequences (Figure S1C). Because of the high genome similarity across primate species, we limited this score to non-primate mammalian species, where unconstrained genome sequences show higher divergence. We therefore excluded all mammalian species that were classified as primates or colugos (primatomorpha). Phylogenetic reconstructions have shown that the genomes of colugo and primate species are quite related, hence colugos can be phylogenetically classified as a sister taxon to primates.[100] Unaligned species were not considered, so counterpart regions which evolved across different branches of mammalian evolution were evaluated for conservation.

Finally, we selected a CS significance cutoff ≥ 8 for consistent amino acid *sequence* similarity conservation across non-primate mammals. We estimated a FDR < 0.01 by randomly extracting 10,000 size-matched sequences from untranslated regions of the

same genes that host sORFs and calculating the distribution of CS scores for these sequences. Hence, amino acid sequences below the CS threshold are defined as ‘conserved proteins’ through mammalian evolution, as opposed to the rest of sequences that encode ‘evolutionarily young’ proteins without conserved protein homologues in non-primate mammals. Of the 7,264 cataloged sORFs and 527 sCDS, 758 sORF-encoded microproteins (10.43%) and 379 sCDS-encoded microproteins (71.37%) were conserved across mammals, including at least 375 sORF-encoded microproteins and 257 sCDS-encoded microproteins with significant conservation in some vertebrate species (translated orthologous regions of chicken, western clawed frog, and/or zebrafish with pairwise E-value < 10^{-4}). Conserved proteins were aligned to an average of 71 non-primate mammalian species, with 95% of ORF sequences aligned to 5 or more species. 69.3% and 59.7% of the non-conserved sORFs could be aligned to at least one and more than five non-primate mammalian species, respectively.

Of note, the fraction of sORF-encoded microproteins with detectable homologues in non-primate mammals was higher for ORFs overlapping protein-coding sequences (~30-35% of uoORFs and intORFs) compared to other ORF biotypes (~4-5% of lncORFs, uORFs and dORFs) (Figure S1D). Conserved sORF-encoded microproteins were longer than young ones (median length, 64 versus 37 amino acids, Figure S1A) and exhibited higher levels of protein similarity than both non-conserved sORF-encoded microproteins as well as untranslated ORF sequences from the same transcripts (Figure S1B).

Furthermore, we searched for signatures of evolutionary protein-coding potential in the set of young sORFs, sCDS, and untranslated controls by running PhyloCSF[80] with default parameters. PhyloCSF scores were calculated across the retrieved multiple alignments for primates and for mammals. Young sORFs displayed similar PhyloCSF[80] scores to untranslated control sequences across primates and mammals, indicating that the codon sequences of young sORFs were not constrained at the protein-coding level (Figure S1E).

Conservation of sORF structures

We firstly adapted a previously published method[101] and used PRANK v.170427[81] to reconstruct ancestral sequences based on the built Cons120 and Cons30 whole-genome multiple alignments. Next, we evaluated the conservation of young sORF, sCDS and untranslated ORF structures (structural conservation) across ancestral sequences in the primatomorpha lineage. An ORF *structure* was considered as conserved in an ancestral region if the ATG translation initiation site (TIS) was present in the same position or within an in-frame window 6 nt downstream of the human ATG position, and if $\geq$ 70% of the sequence did not contain stop codons truncating the ORF. For the cases with different alignments in Cons120 and Cons30, we selected the one with the highest level of conservation. The considered lineages were humans (<6-8 Million years ago (Mya), age 0), old world monkeys (~35 Mya, age 1) and primatomorpha (including primates and the only two living colugo species; ~88 Mya, age 2). Hence, these categories define the most distant ancestral primatomorpha sequence predicted to contain the full ORF structure. These ORFs could be fixed across all species from the lineage, they could be segregating and only be present in a subset of species from the lineage, or they might have evolved convergently in independent primate and non-primate lineages (although with highly divergent protein sequences).

The numbers of species assigned to each lineage and the genome releases used to map sequencing datasets are described in Table S1.

Expression and translation of sORFs in mammals

To determine if the absence of a conserved ORF *structure* in a specific species was supported by the absence of ribosome occupancy and/or translation in the same region, we downloaded human, macaque and mouse Ribo-seq data from brain tissue, three replicates each (ArrayExpress accession number E-MTAB-7247[23]). Ribo-seq reads were trimmed for adapters, filtered to remove common rRNA, tRNA and mitochondrial RNA contaminants, and mapped to the human, macaque, or mouse genome using STAR v2.5.2b[82] with maximum of 2 mismatches. Next, we ran ORFquant 1.00[46] to call translated ORFs in the human brain and subsetted 830 translated sequences that were also found in the set of 7,264 cataloged sORFs. Subsequently, we extracted local sORF coordinates from non-human species genomes using LiftOver chain alignments and ran BEDTools v2.27.1[83] to quantify the number of reads overlapping these sequences. In both macaque and mouse species, the absence of conservation of sORF structures led to a strong significant drop in the number of mapped Ribo-seq reads (macaque: median of 79 reads vs 14 reads for conserved and non-conserved ORF structures, Wilcoxon signed rank test p-value = 9.11 x 10^{-33}; mouse: median of 97 reads vs 8 reads for conserved and non-conserved ORF structures; Wilcoxon signed rank test, p-value = 2.58 x 10^{-61}; Figures 1F; Figure S1G), while the levels of ribosome occupancy remained constant in human. We additionally applied a simple binomial probability test to determine how many of the macaque and mouse brain counterpart regions had significant Ribo-seq periodicity biases (p-value < 0.01), as previously done by Patraquim and colleagues.[102] In support of our method, counterpart regions of human brain ORFs without conserved structures displayed lower periodicity biases in macaque and mouse (Figure S1H). However, we still found a group of ORFs without conserved structures but with significant periodicity in macaque (27%) and mouse (18%). Thus, a small proportion of the non-conserved ORFs are still translated into shorter ORFs (<70% of human length sequence) or use alternative translation initiation or splice sites.

Moreover, we ran ORFquant to call translated ORFs in the macaque brain and found that counterpart regions of human ORFs without conserved structures were depleted from actively translated ORFs in macaque (Test of equal proportions, p-value < 0.05; Figure 1G). In addition, we retrieved a unified set of translated ORFs from several mouse tissues[103] and determined how many of these ORFs overlapped regions aligned to human sORFs with different levels of conservation (Figure S1I). As expected, mouse regions that could be aligned to evolutionarily young ORFs contained a very low proportion of translated ORFs (5.30%) compared to

conserved sORFs present in mammals (39.66%). This indicated that, for human brain translated ORFs, the corresponding truncated ORF sequences were not actively translated in other non-human species.

We next estimated how many counterpart regions potentially containing cataloged sORFs were supported by RNA expression evidence in other species. Chimpanzee, macaque, mouse, cow, dog, horse, elephant and opossum transcriptomes and genomes were retrieved from Ensembl v.98. Chimpanzee and macaque RNA-seq data from brain, heart, liver and testis were downloaded from Gene Expression Omnibus with accession code GSE69241.[104] RNA-seq reads were trimmed for adapters and mapped to each corresponding genome using STAR v2.5.2b[82] with a maximum of 4 mismatches. Chimpanzee and rhesus macaque RNA-seq data from the four tissues were combined to assemble a species-specific reference-guided transcriptome with Stringtie v1.2.1[85] (parameters -M 0.5 -j 3 -p 4 -f 0.2). For each species, we then searched for annotated and/or assembled transcripts overlapping the previously generated LiftOver regions. We found that 90-93% and 74-92% of the counterpart regions of conserved sORFs overlapped transcripts expressed in primate and non-primate mammalian lineages, respectively. In contrast, evolutionarily young sORFs were less commonly expressed in rodents (60%), especially in more distant mammalian lineages, such as ferae (32%). This indicated that, in mammals, the presence of conservation is often linked to the presence of expression (Figure S1J). A similar trend could be observed for the expression of primate-specific *versus* mammalian- and vertebrate-specific $sORF_{3\text{-}15\ aa}$ (Figure S4B).

Mode of evolution of sORFs

To identify the mode of evolution of evolutionarily young sORFs, we ran BLASTp to inspect whether any of the ORF sequences displayed significant homology to any other annotated protein in the human, macaque, mouse, cow, dog, horse, elephant and opossum proteomes (Ensembl v.98). For sORFs with significant matches (E-value < 10^{-4}), we further assessed if the candidate orthologous protein was translated in the same aligned genomic region or a different one by analyzing the previously generated LiftOver coordinates for these species. Orthologs found in a different region were defined as 'CDS duplications', while the ones found in the same aligned genomic region were defined as 'CDS fissions', provided that both the sORF and the rest of the human protein exhibited homology to different parts of the same protein in the other species.

For the remainder of the sequences, we re-analyzed the generated multiple and LiftOver alignments to trace the sORFs back to their evolutionary origins. We classified an sORF as '*de novo*' if the age of the aligned region predated the age of the ORF, hence being able to spot the mutations responsible for the birth of the ORF sequence. For the cases where the region could not be aligned beyond the lineages where the ORF *structure* was conserved, we downloaded transposable element annotations from RepeatMasker v.4.1.0[86] and inspected whether both the ORF and the region emerged as a result of the insertion of an endogenous retrovirus (EVR) or *alu* element (category 'EVR/*Alu* derived'). The mode of evolution of the remaining sORF sequences could not be correctly assessed with the available data, since the ancestral region evolved at the same time as the 'orphan' ORF sequence, and we classified these cases as 'not known'. A comparison of the main findings of our approach and the one recently developed by Vakirlis and colleagues[7] is available in the following section.

Comparison of the modes of evolution with a previous resource

We compared our resource on modes of evolution with a recent study from Vakirlis et al. that evaluated the modes of evolution of 715 ORFs structrures.[7] Vakirlis et al. used aligned sequences from the UCSC 100-way phylogenetic tree to reconstruct the ancestral sequences and evaluate frame conservation –independent of the presence or absence of an initiating ATG codon- and the expression of the loci in other species. This set of 715 ORFs was extracted from a published ORF dataset,[6] which was one of the seven datasets included in the GENCODE sORF resource that we included in our study. Of these 715 ORFs evaluated by Vakirlis et al., 452 overlapped our sORF list. The reasons why 263 ORFs were excluded from our dataset were diverse: some overlapped in-frame annotated coding sequences and pseudogenes, had a length under the selected cutoff (< 16 aa), or could not be fully mapped to annotated GENCODE transcripts. Vakirlis et al. found 155 out of 715 ORFs to have evolved *de novo*, of which 94 overlapped with our ORF set. We similarly classified 60 out of these 94 (63.82%) ORFs as *de novo* evolved, while for 31 cases we could not assign any mode of evolution ('not known'). Only for 3 out of 94 (3.19%) cases we found a duplication event that would disqualify the ORF as having emerged *de novo* as per our analysis. These are: (1) c10riboseqorf103 (RPARP-AS1_104210065_114aa), which is encoded by an antisense lncRNA and the ORF contains a partial duplicated region which overlaps with the 3′ UTR of the sense gene *c10orf95*, (2) c19riboseqorf102 (ZNF585A_37701369_35aa), which is a uORF harboring a partially duplicated region of the local neighbor gene *AC012309.1*, and (3) cXnorep31 (ZNF81_47696378_89aa), which contains an exon derived from an *AluSz* element that is also integrated in the coding sequences of several primate genes. Moreover, Vakirlis et al. reported 7 *de novo* ORFs as being human specific. Three out of these 7 overlapped the ORF list evaluated in our study and we also classified them as emerged *de novo*, although we defined an older primatomorpha origin for one of the ORFs (MALAT1_65266767_39aa / c1riboseqorf84) and a human origin for two of them (PTPRF_43996733_27aa / c1riboseqorf84 and TTC9C_62496064_18aa / c11riboseqorf70). Our study additionally identified 160 human-specific *de novo* ORFs, including 147 cases that were not evaluated previously by Vakirlis et al. and likely therefore not yet reported as *de novo* human-specific ORFs. We classified an additional 13 ORFs as being human specific that Vakirlis et al. reported as being conserved in non-primate mammals. Although our results generally overlap very well, the observed discrepancies in the mode of evolution for some of the cases might be due to differences in the parameters for the computational methods (*i.e.*, we require conservation of the initial ATG codon) and the selection of distinct genome-wide alignments with different numbers of primate, mammalian and vertebrate species.

Candidate selection for PRISMA of microproteins translated from recently evolved sORFs

We selected 45 evolutionarily young microproteins (CS < 8, longer than 15 aa) to investigate their interactomes with PRISMA. This included 41 microproteins putatively encoded by cataloged Ribo-seq ORFs (32 lncORFs, 5 uORFs, 4 uoORFs) as well as 4 microproteins putatively encoded by novel sORFs outside annotated gene regions discovered by us in a recent study (3 lncORFs and 1 uORF).[22] We additionally included 15 microproteins translated from conserved mammalian sORFs to compare the interactomes of young and conserved proteins of similar protein sizes. Four out of the 15 selected conserved microproteins were translated from lncORFs, three from uORFs and eight were sCDS (annotated small proteins). Four of the conserved sCDS (MIEF1-MP, MTLN, NDUFB3 and MRPL33) evolved during vertebrate evolution and had been included in interactomics studies before.[30,31,105,106] The properties, conservation and mode of evolution of each candidate were calculated and reported in Table S2.

In addition to the levels of conservation, we based the selection of the PRISMA candidates on the likelihood of these sORFs to be translated into stable microproteins. Therefore, we collected evidence for sORF translation in multiple datasets, as well as the exogenous and endogenous detectability of their translation products (microproteins). Moreover, we considered the potential relevance of the sORF or the host gene in disease. Details on how we collected all this information are described below.

Ribo-seq datasets

We counted the number of Ribo-seq studies that supported the translation of candidate sORFs. To this end, we retrieved evidence from the study of Mudge and colleagues that collected data from seven Ribo-seq studies to create a first consolidated sORF catalog.[2]

ORF databases

We investigated how many of the candidate sORFs were reported in three public ORF annotation databases: SmProt,[25] MetamORF[26] and sORF.org.[27] Nine were detected in one database, 12 in two and 16 in all three databases.

In vitro *translation evidence*

We searched for candidate sORFs that were able to produce detectable microproteins in coupled *in vitro* transcription:translation assays, as described and reported in our previous study,[22] and found 23 out of 24 candidates that were tested.

Antibody-based microprotein detection after overexpression

We surveyed our previous study[22] as well as three others[5,6,28] for sORFs that produced detectable microproteins after ectopic expression in cultured human cells. In all four studies, epitope-tagged microproteins were overexpressed in human cell lines and detected either via immunoblotting or immunofluorescence. Additionally, in the current study we overexpressed 31 microproteins with a C-terminal 3xFLAG-tag and detected protein expression of 26 candidates by immunofluorescence (details in STAR Methods section detection of overexpressed microproteins by immunofluorescence and Data S1).

Endogenous mass spectrometric evidence

We collected protein expression evidence of 15 candidate sORF-encoded microproteins from our previous study, based on targeted mass spectrometry (selected reaction monitoring, SRM) data,[22] and acquired evidence for four more candidates (PVT1-MP, MRPL33, NDUFB3 and MIEF1-MP) in a targeted mass spectrometry assay (parallel reaction monitoring, PRM) performed in this study (details in STAR Methods section parallel reaction monitoring (PRM) proteomics). We additionally searched for identified peptide spectrum matches (PSMs) that uniquely mapped to sORF-encoded microproteins in a previous meta-analysis of 16 published MS searches, including both shotgun MS and HLA peptidomics studies.[2] For 32 sORFs, we found at least one PSM mapping to their encoded amino acid sequences. We also retrieved evidence of sORFs protein expression from PeptideAtlas (2022-01).[29] For seven sORFs, we found evidence of PSMs uniquely mapping to their respective translated microproteins in HLA peptidomics datasets. However, for five out of seven cases this evidence was limited to single peptide identifications. While these peptides were not assigned to any other translated sequences in the human proteome, further manual curation will be required to validate the collected MS evidence, especially when only supported by a unique PSM.

Candidate disease relevance and prior indications of potential microprotein function

We collected indications for disease relevance to prioritize candidates. To this end, we took into account whether the microprotein host gene was a presumed lncRNA that had been implicated in disease, anticipating that the encoded, but previously missed microprotein might have contributed to the observed phenotypic effect. We interrogated the manually curated database EVLncRNAs 2.0,[107] which contains lncRNAs whose function and disease association was validated by low-throughput and targeted experiments. Similarly, we searched for sORFs experimentally interrogated in different functional assays to find candidates with reported biologically active roles. First, we used the output data from the functional assay on 553 ORFs from Prensner and colleagues[5] and found 11 ORFs whose microproteins were included in our PRISMA design. We extracted two functional scores based on CRISPR phenotype and transcriptional activity score (transcriptional perturbation after overexpression). An ORF scored positive if the CRISPR phenotype was significant in the original study (CRISPR phenotype = 1) and/or the transcriptional activity score was higher than or equal to 0.2. None of the 11 microproteins had an effect on cell growth, but five young (RP11-140K17.3-MP, DANCR-MP, PRR34-MP, SNGH8-MP, and SNHG6-MP) and three conserved microproteins (MTLN, MKKS-uORF-MP, and MIEF1-MP) altered cellular transcription profiles after overexpression. Second, we selected 771 sORFs translated in the dataset published by Chen and colleagues,[6] one of the studies selected for the GENCODE Ribo-seq ORF catalogue, and retrieved CRISPR and Perturb-seq scores from the same study for 10 ORFs encoding microproteins included in our PRISMA design. An ORF was positive if the CRISPR score was significant in the original study (p-value $\leq$ 0.5) and/or the Perturb-seq assay found pathways affected significantly after knocking-out the ORF. Four conserved microproteins (RNF10-uORF-MP, TUG1-MP, MIEF1-MP and IFRD1-uORF-MP) influenced cell viability and one

young microprotein (PPP1R15A-uORF-MP) and three conserved microproteins (MIEF1-MP, LINC00881-MP and IFRD1-uORF-MP) scored in the Perturb-seq assay. Combined, the merger of both studies yielded 13 ORFs whose microproteins were included in our PRISMA design that either affected cellular growth or transcription profiles in at least one of the two investigated studies, of which seven would fall into the 'young' category, and six would be considered conserved. Additionally, of the 221 peptides$_{3\text{-}15\ aa}$, 23 had been previously interrogated by Chen and colleagues, while Prensner and colleagues only included candidates >15 aa. Of those peptides$_{3\text{-}15\ aa}$ included by Chen and colleagues, six scored positive, *i.e.*, impacted cell viability.

Summary of selection criteria

The selection criteria for each candidate are summarized in Table S2. Briefly, 30 out of 45 young and 11 out of 15 conserved sORFs were found to be translated in at least two independent sORF studies. Exogenous protein-level support for encoded microproteins was obtained for 44 sORFs from either *in vitro* translation assays (n_{young} = 21/45; $n_{conserved}$ = 3/15) or antibody-based detection after overexpression (n_{young} = 39/45; $n_{conserved}$ = 13/15). Mass spectrometric detection supported endogenous production of 29 young and 12 conserved microproteins in human cell lines and tissues. Seven young and six conserved microproteins were translated from ORFs with hits in CRISPR and overexpression screens, and 22 were translated from lncRNAs implicated in disease, including cardiovascular disease (10) and cancer (17).

Detection of overexpressed microproteins by immunofluorescence

Synthetic gene fragments containing the codon-optimized coding sequence of candidate microproteins with a C-terminal 3xFLAG tag were synthesized and cloned into a customized plasmid for mammalian expression by Genewiz Europe (Leipzig, Germany; constructs available upon request). The 3xFLAG-tagged human microproteins were overexpressed in HeLa cells and visualized using immunofluorescence as described by us previously.[22] Human HeLa cells were grown on glass slides in 12-well plates for 24 h and transfected with plasmids encoding c-terminally 3xFLAG-tagged microproteins using Lipofectamine 2000 according to manufacturer's instructions. The plasmids used are available upon request. 24 h post transfection cells were fixed with 4% paraformaldehyde (PFA) for 10 min at room temperature (RT) and washed three times with ice cold phosphate-buffered saline (PBS). Cells were permeabilized and blocked for 1 h with 2.5% bovine albumin serum (BSA), 10% anti-goat serum (NGS) and 0.1% Triton X in PBS. After washing the cells, overexpressed microproteins were stained with anti-FLAG mouse monoclonal antibody (1:500 in PBS with 5% BSA, F1804, Sigma Aldrich) for 1 h at RT. Mitochondria (1:1000 rabbit anti-ATPIF1 in PBS with 5% BSA, #13268, Cell Signaling Technology, Danvers, MA, USA) and in the case of LINC01128-MP overexpression clathrin-coated vesicles (1:100 rabbit anti-Clathrin Heavy Chain (P1663) in PBS with 5% BSA, #2410, Cell Signaling) were co-stained in this step. Afterwards cells were washed and incubated with fluorochrome-labeled secondary antibodies (1:500 in PBS with 5% BSA, Alexa Fluor 488 anti-rabbit and Alexa Fluor 594 anti-mouse (Invitrogen, Carlsbad, CA, USA) for 30 minutes at RT. Cells were washed again, stained with 4-6-diamidino-2-phenylindole (NucBlue Fixed Cell ReadyProbes Reagent, R37606, Thermo Fisher) for 5 minutes at RT and mounted onto glass slides using ProLongTM Gold antifade reagent (Molecular Probes; InvitrogenTM). Images were taken with a LEICA SP8 confocal microscope using a 63x objective and analyzed using ImageJ (v1.53c).[108]

PRISMA of microproteins translated from cataloged sORFs

Experimental setup

We adapted the PRISMA assay from the PRISMA assay introduced previously by others and ourselves.[17–20] Each of the 60 putative microproteins was divided into 15 aa long, overlapping peptides (tiles) with an offset of eight aa. This resulted in 478 tiles (minimum of two, maximum of 21, average of eight tiles per microprotein). To evaluate if arbitrary peptides derived from untranslated RNA sequences would serve as a suitable control for our screen, we generated a set of arbitrary peptides which we translated *in silico* from non-coding regions (5′ UTRs or lncRNA exons without translation in our Ribo-seq data). These sequences start with ATG codons and are located in the genes hosting the 271 microproteins and peptides included in the PRISMA analysis, with length-matched distributions. We only considered regions that were not covered by annotated coding sequences nor by sequences from the set of 7,264 sORFs$_{>15\ aa}$ and 221 sORFs$_{3\text{-}15\ aa}$ analyzed in our manuscript. We found that the 45 young microproteins included in the PRISMA analysis present similar amino acid compositions compared to untranslated sequences, as observed in the PCA (Figure S2D). This is in line with our observation that most of the young microproteins recently emerged *de novo* from non-coding RNA sequences. Moreover, we generated 10,000 sets of shuffled sequences for each of the 45 young microproteins included in the PRISMA screen and compared the numbers of SLiMs predicted in these sequences. The numbers of SLiMs in young microproteins and shuffled sequences are not statistically different (978 SLiMs in young microproteins and an average of 948 SLiMs in shuffled sequences, p-value = 0.16, Figure S2E). Because of this, we expect the extent and specificity of the interactomes of untranslated sequences, shuffled sequences and young microproteins to be rather similar, indicating that arbitrary or random peptides would not serve as a helpful control. Instead, we included four characterized control peptides derived from the proteins SOS1 and GLUT1 that had been investigated in previous proteomic interaction screens.[18,32] In total, 490 peptides were spot-synthesized (SPOT synthesis technology) on three cellulose membranes (JPT Inc., Berlin, Germany), with the GLUT1 and SOS1 peptide controls present on each membrane. Each spot carries approximately five nmol of peptide covalently bound to the cellulose-ß-alanine-membrane. Peptides were acetylated at their free N-termini to enhance stability and to better recapitulate the uncharged nature of a protein backbone. All three biologically distinct membranes were ordered and processed in triplicates. Sequences of spotted tiles can be found in Table S3. The spotted peptides have been referred to as "baits", the proteins that are bound by these peptides as "prey".

Protein lysate preparation

HEK293T/17 cells were grown in 14 cm dishes (Sarstedt) as described above. All following steps were performed on ice and only ice-cold buffers were used. Cells were washed with PBS, scraped, transferred into falcon tubes and centrifuged for 5 min at 1000 g. After an additional wash with PBS cell pellets were resuspended in lysis buffer (50 mM HEPES pH 7.6 at 4°C, 150 mM NaCl, 1 mM EGTA, 1 mM MgCl2, 10% Glycerol, 0.5% Nonidet P-40, 0.05% SDS, 0.25% sodium deoxycholate and cOmplete™ EDTA-free protease inhibitor (Roche) (0.7 mL per 14 cm dish) and incubated for 30 min on ice. Five μL (1250 U) of Benzonase (Merck) were added, followed by another 15 min incubation and 15 min centrifugation step at 20,000 g. The supernatant was transferred into a fresh tube and the protein concentration was determined with the Pierce™ BCA Protein Assay Kit (Thermofisher Scientific) following manufacturer's instructions. The protein concentration was adjusted to 5 mg/mL with lysis buffer. The protein extract was directly used for the PRISMA assay.

Sample preparation for mass spectrometric analysis

Membranes with spot-synthesized peptides were equilibrated at RT for 15 min in wash buffer (50 mM HEPES pH 7.6 at 4°C, 150 mM NaCl, 1 mM EGTA, 1 mM MgCl2, 10% Glycerol), blocked with 1mg/mL tRNA (Invitrogen; diluted in wash buffer) for 10 min and washed again twice with wash buffer for 5 min. Afterwards, membranes were incubated with HEK239T protein lysate (5 mg/mL) for 2 h at 4 °C while shaking, followed by 3 washing steps with a wash buffer for 5 min at 4 °C, and were dried for 15 min at RT.

Peptide spots were punched out using a 2 mm mouse ear puncher and transferred directly into 20 μL of urea sample buffer (6M Urea, 2M Thiourea, 10mM HEPES). Samples were reduced in 12 mM dithiothreitol (DTT) solution for 30 min at RT and alkylated in 40 mM chloroacetamide for 45 min at RT in the dark. To digest proteins bound to the spotted peptides, samples were diluted with 100 μL of 50mM ammonium bicarbonate (pH 8.5) buffer containing trypsin (Promega; 5 μg/mL) and LysC (Wako; 5 mAU/mL), and incubated overnight at RT. The proteolytic digestion was stopped by adding 4 μL 25% trifluoroacetic acid. Peptides were extracted and desalted using StageTip protocol.[109]

LC-MS/MS

Peptides were eluted using Buffer B (80% acetonitrile and 0.1% formic acid), organic solvent was evaporated using a speedvac (Eppendorf) and samples were diluted in Buffer A (3% acetonitrile and 0.1% formic acid). Peptides were separated on a 20 cm reversed-phase column (inner diameter 75 μm, packed with ReproSil-Pur C18-AQ 3 μm resin (Dr. Maisch GmbH)) using a 45 min gradient with 250 nl/min flow rate of increasing Buffer B concentration on a High Performance Liquid Chromatography (HPLC) system (ThermoScientific). Peptides were ionized using an electrospray ionization (ESI) source (ThermoScientific) and analyzed on an Orbitrap Fusion instrument (ThermoScientific). Precursor survey scans were performed at 120K resolution with a 2×10^5 ion count target. Dynamic exclusion for selected precursor ions was 30 s. MS/MS was performed with a 1.6 m/z isolation window, HCD fragmentation with normalized collision energy of 32, ion count target of $1x10^4$ and maximum injection time of 300 ms. The instrument was operated in top speed mode with 3 s cycles. Replicates were measured in batches with a different run order for each batch. A blank run was placed after each analytical run.

Data analysis

The resulting raw files were analyzed using the MaxQuant software package 1.6.0.1.[56] The internal Andromeda search engine was used to search MS2 spectra against a decoy human UniProt database (Human.2019-07) and an in-house database containing PRISMA peptide and microprotein sequences. The search included variable modifications of methionine oxidation, N-terminal acetylation, deamidation (N and Q) and carbamidomethyl cysteine as fixed modification. The FDR was set to 1% for peptide and protein identifications. Unique and razor peptides were considered for quantification. Retention times were recalibrated based on the built-in nonlinear time-rescaling algorithm. MS2 identifications were transferred between runs with the "match between runs" function. The integrated LFQ quantitation algorithm was applied.

Following analyses were done using R v.3.6.1[87] and adapted from Meyer and colleagues[18] with slight modifications. The resulting text files were filtered to exclude reverse database hits, potential contaminants, and proteins only identified by site. Missing LFQ-values were imputed with random noise simulating the detection limit of the mass spectrometer. Imputed values were taken from a log normal distribution with 0.3x the standard deviation of the measured, logarithmized values, down-shifted by 1.8 standard deviations. By doing this, we obtained a distribution of quantitative values for each protein across samples. We excluded replicates with sample identifications of over two standard deviations away from its other replicates, as well as samples whose correlation value was over two standard deviations away from the other correlations between replicates. This led to the exclusion of two peptide spots from interactome analyses (Table S3). For determination of specific interactions, *i.e.*, to separate specific binders from background, we compared protein identifications in each peptide spot against all other peptide spots excluding spots of the same microproteins using moderated t-tests (limma v3.40.6[110]). Only proteins with at least two valid values for the peptide spot were considered. The resulting p-values were adjusted using Benjamini-Hochberg correction. Adjusted p-values and fold-changes (log_2 space) were plotted as volcano plots. To determine significance cutoffs, we used a graphical formula combining a fold-change and p-value cutoff[18,111]: $-log\,10\,(p) \geq \frac{c}{|x| - x0}$ with x: enrichment factor of a protein, p: p-value of adjusted moderated t-test, x_0: fixed minimum enrichment, c: curvature parameter. The curvature parameter c determines the maximum acceptable p-value for a given enrichment x. The parameters c and x_0 can be optimized based on prior knowledge of known true and false positives.[32,111] Here, cutoffs were chosen according to known interaction partners of the SOS1 and GLUT1 control peptide.[111] This resulted in a cutoff of x0 = 3, c = 4 that was applied to all other peptide spots. Ultimately, the PRISMA approach enabled us to define significant interactors for

synthesized peptides individually. The total interactome of one microprotein was defined as the summary of interactors detected in all synthesized peptides that were derived from that microprotein.

Quality control by replicate measurement assessment

Principal component analysis showed that no batch bias was identified between the replicate membranes (data not shown). Further, for all three membranes triplicate measurements of each spotted peptide correlated well with a median Pearson's R of 0.73, 0.72, and 0.87, respectively, and were significantly higher than the median of correlations of random triplets (Figure S2A). This was similar to what was observed in a previous peptide array screen.[18] The number of identified proteins per peptide spot ranged from 159 to 2,380 and was comparable across membranes (median of 973, 1,012 and 1,011 IDs for membrane 1, 2 and 3, respectively) (Figure S2B).

Quality control by evaluation of assay control peptides

The spotted SOS1 peptide is known to interact with SH3-domain containing proteins via its proline-rich motif, while motif disruption in the mutant peptide leads to the loss of this interaction capability (loss-of-interaction mutant).[18,32] In the case of GLUT1, a proline (P) to leucine (L) mutation creates a dileucine motif in the mutant peptide which mediates the binding to adaptor and clathrin proteins.[18] Interactions with clathrins do not occur in the wild type peptide and lead to aberrant endocytosis of GLUT1 in GLUT1 deficiencies (gain-of-interaction mutant).[18] Reassuringly, we only found clathrins to be significantly enriched in pulldowns of the GLUT1 mutant peptide but not of the wild type peptide (Figures 2D and S2C) and detected up to eight of the nine known SOS1 binding partners (Figures 2C and S2C). As expected, mutation of the SOS1 proline-rich motif led to the loss of most SH3 domain proteins except for CD2AP, GRB2 and BIN1 (Figure S2C).

Overall, the assay controls demonstrated the ability of our peptide array approach to detect biologically relevant PPIs as well as its specificity to the spotted peptide sequence. For 481 out of 488 analyzed peptides, we identified between 1 and 94 significant protein interactors (with a median of 14), resulting in 13 to 333 interactors per microprotein (with a median of 107 interactors) (Figure 2B; Table S3). Interactors that were detected in the interactomes of multiple microproteins tended to have lower interaction scores (product of fold change and p-value) than interactors that were found in fewer or only one microprotein interactome. However, this is likely due to the comparison approach against all other peptides, which will penalize proteins that are detected in many peptide pulldown (Figures 2B and S2F).

Quality control based on bait identification for PRISMA of young microproteins

As part of the PRISMA quality control we assessed if the spotted peptides (baits) were identified and enriched in the expected samples, i.e., in the samples that contained the part of the membrane the bait peptide was synthesized on. We reasoned that the high amount of synthesized peptides should lead to their identification by peptide-spectrum matches in case a suitable tryptic peptide is produced upon enzymatic digestion. Thus, we only considered bait identifications that were detected "by MS/MS" in at least two replicates, while hits derived "by matching" were excluded. Because of the short length of the baits (15 aa), the possibilities to produce tryptic peptides suitable for MS is rather limited and indeed we do not detect all, but 193 out of 480 unique baits. 108 of those (56%) were exclusively identified in the expected samples (data not shown). 79 baits were enriched in the expected sample, but also in other samples. However, for 71 of those the median LFQ intensity across all three replicates was higher in the correct samples compared to LFQ intensities in unexpected samples. In seven cases the bait was detected with a higher intensity in an unexpected sample than in the correct sample, and seven baits were only enriched in unexpected samples (data not shown). In total four samples were completely excluded from the analysis and 13 were flagged and only used to determine the interactome of the entire microprotein but not to investigate motif-driven interactions (Table S3).

Expression of microproteins and peptides$_{3\text{-}15\ aa}$ in HEK293 cells

Since we used a protein lysate from HEK293T cells for the PRISMA analysis, we assessed the expression level of the microprotein and peptide-encoding genes in this cell line. Therefore, we downloaded a public RNA sequencing dataset of HEK293T cells (NCBI Sequence Read Archive (SRA) accession codes SRR1107836 and SRR1107837[77]) and calculated the counts per million (CPM) from the mapped and quantified raw reads. A gene was determined as expressed if the mean of the two runs per gene was above or equal to 1. We detected the genes of 39/60 microproteins (65%) (Table S3) and 194/221 peptides$_{3\text{-}15\ aa}$ (88%) (Table S4) as expressed in HEK293 cells.

Annotation and enrichment of essential proteins detected in microprotein interactomes

We downloaded a list of proteins that were shown to be essential for survival of human cells in a study by Blomen and colleagues.[33] We only included genes that affected cell viability in both tested cell lines (KMB7 and HAP1). This resulted in a list of 1,734 proteins based on which we annotated microprotein interactors as essential proteins. We calculated if specific microprotein interactomes were enriched for essential proteins using Fisher's exact test (Table S3).

Phylogenetic origin of microprotein interaction partners

We extracted the phylogenetic origins of microprotein interactors from a public resource published by Zhang and colleagues (http://gentree.ioz.ac.cn/download.php; "Ensembl Ver95 (hg38)"),[112] who assigned annotated protein-coding genes to 14 different evolutionary branches. We used this resource to annotate the evolutionary origin of 2,357 out of 2,423 microprotein interactors identified in PRISMA (Table S3). We collapsed the 14 branches into four evolutionary groups: Vertebrates (Branch 0 - 2: Euteleostomi, Tetrapoda

and Amniota), Mammals (Branch 3 - 7: Mammalia, Theria, Eutheria, Boreoeutheria, Euarchontoglires), Primates (Branch 8 - 12: Simiiformes, Catarrhini, Hominoidea, Hominidae, Homininae) and Humans (Branch 13).

Computational prediction of disordered regions and short linear motifs within microproteins and peptides

Intrinsically unstructured (disordered) regions in peptide sequences were predicted using IUPred v.1.0 in the "short" disorder mode and disorder values were averaged over the sequence.[36] For the detection of short linear motifs (SLiMs), also called eukaryotic linear motifs (ELMs), the 'elm_classes.tsv' file (version 1.4; 15 January 2018) was downloaded from the ELM resource for functional sites in proteins.[37] We then filtered the peptide sequences for matches to any of the motifs falling in regions with an average disorder value $\geq$ 0.5. Five of the 60 microproteins subjected to PRISMA did not harbor any predicted SLiMs, the remaining 55 microproteins contained 429 SLiMs within disordered regions. Of those, 412 were captured within 159 of the tiled peptides spotted for the PRISMA screen (Table S3). Moreover, 174 of 221 peptides$_{3\text{-}15\text{ aa}}$ contained 514 SLiMs within disordered regions all captured in the PRISMA approach (Table S4).

Detection of protein domain-SLiM matches in PRISMA interactomes

We first annotated known protein domains for each microprotein and peptide interactor using the R packages "ensembldb" and "EnsDb.Hsapiens.v86."[97] Next we extracted SLiMs that were known to bind the respective protein domains from a public resource published by Kumar and colleagues (http://elm.eu.org/; "elm_interactiondomains.tsv").[41] We reported a domain-SLiM match when a SLiM was present in a microprotein tile or peptide$_{3\text{-}15\text{ aa}}$ that bound an interactor carrying the SLiM-binding protein domain. In total we detected 47 protein domain-SLiM matches within disordered regions of 34 microprotein tiles (Table S3) and 30 protein domain-SLiM matches within 18 peptides$_{3\text{-}15\text{ aa}}$ (Table S4).

Kinase-enrichment by microproteins with kinase-related SLiMs

In total, 117 out of 481 microprotein tiles were predicted to carry one of 35 different kinase phosphorylation and docking motifs within putative disordered microprotein regions extracted from the ELM resource for functional sites in proteins.[37] We detected 158 microprotein-kinase interactions in the entire interactome screen, 17 of which were detected in the interactomes of fifteen tiles (derived from six young and three conserved microproteins) of the 117 tiles harboring a kinase phosphorylation or docking motif. Fisher's exact test revealed that kinases were not enriched in interactomes of microprotein tiles that carry a kinase phosphorylation or docking motif within disordered regions (p-value = 0.079, Fisher's exact test).

Phosphorylation of kinase-binding microproteins with domain-SLiM matches

We investigated the phosphorylation of the nine microproteins from the SLiM-domain match analysis that interacted with kinases and carried phosphorylation or kinase docking motifs (RP11-12K22.1-MP, JHDM1D-AS1-MP, RP11-620J15.3-MP, SLCO5A1-uORF-MP, GAS5-MP, ABR-uORF-MP, MKKS-uORF-MO, RP11-140K17.3_2-MP and THAP7-uORF-MP, Table S3). Therefore, 3xFLAG-tagged microproteins were overexpressed, immunoprecipitated and analyzed by MS as performed previously[22] with slight modifications. HEK293T/17 were seeded in triplicates on poly-D-Lysine (Sigma, Germany) coated 10 cm dishes and transfected with 28 μg plasmid-DNA of FLAG-tagged microproteins using TransFectin (BioRad, California) following manufacturer's instructions. Two days post transfection cells were washed twice with ice-cold phosphate-buffered saline (PBS), scraped in 1.5 mL ice-cold PBS and transferred into Eppendorf tubes. After centrifugation at 950 g for five min at 4 °C, cell pellets were lysed in 200 μL lysis buffer (150 mM NaCl, 50 mM Tris pH 7.5, 1% IGPAL-CA-630, 2x Complete protease inhibitor without EDTA) for 30 min on ice. Lysates were centrifuged at 20,800 g for 15 min at 4 °C and supernatants were added to 30 μL 50% antibody-coupled magnetic bead solution (M2-magnetic beads, Sigma, Germany) and 300 μL wash buffer 1 (150 mM NaCl, 50 mM Tris pH 7.5). Beads were washed 3x in 150 μL wash buffer 1 before usage. After incubating the samples for 2 h at 4 °C in an overhead shaker, samples were washed once with 1 mL wash buffer 2 (150 mM NaCl, 50 mM Tris pH 7.5, 0.05% IGPAL-CA-630) and three times with wash buffer 1. Supernatants were removed and magnetic beads were frozen at 80 °C until analyzed by mass spectrometry. Beads were resuspended in 20 μL urea buffer (6 Murea, 2 Mthiourea, 10 mM HEPES, pH 8.0), reduced for 30 min at 25C in 12 mM DTT solution, followed by alkylation in 40 mM chloroacetamide for 20 min in the dark at 25 °C. Samples were first digested with 0.5 μg endopeptidase LysC (Wako, Osaka, Japan) for 4 h. After adding 80 μL 50 mM ammonium bicarbonate (pH 8.5) samples were digested with 1 μg sequence grade trypsin (Promega) overnight at 25 °C. The peptide-containing supernatant was removed and collected into a fresh tube. Beads were washed twice with 50 μL 50mMammonium bicarbonate (pH 8.5) and the supernatants were pooled. Samples were acidified by adding 1 μL formic acid to stop the digestion.

Peptides were extracted, desalted and diluted as described in the previous section for PRISMA. Peptides were separated on a reversed-phase column (20 cm fritless silica microcolumns with an inner diameter of 75 μm, packed with ReproSil-Pur C18-AQ 1.9 μm resin (Dr. Maisch GmbH)) using a 90 min gradient with a 250 nL/min flow rate of increasing Buffer B concentration (from 2% to 60%) on a High-Performance Liquid Chromatography (HPLC) system (Thermo Fisher Scientific) and ionized using an electrospray ionization (ESI) source (Thermo Fisher Scientific) and analyzed on an Thermo Q Exactive Plus instrument, which was run in data dependent mode selecting the top 10 most intense ions in the MS full scans, selecting ions from 350 to 2000 *m/z*, using 70 K resolution with a 3×10^6 ion count target and 50 ms injection time. Tandem MS was performed at a resolution of 17.5 K. The MS2 ion count target was set to 5×10^4 with a maximum injection time of 250 ms. Only precursors with charge state 2–6 were sampled for MS2. The dynamic exclusion duration was set to 30 s with a 10-ppm tolerance around the selected precursor and its isotopes.

Data were analyzed using MaxQuant v1.5.2.8. The internal Andromeda search engine was used to search MS2 spectra against a human UniProt database (HUMAN.2017-01) and an in-house bait protein sequence database containing forward and reverse sequences. The search included variable modifications of methionine oxidation, N-terminal acetylation and serine, threonine and tyrosine phosphorylation, and fixed modification of carbamidomethyl cysteine. Minimal peptide length was set to seven amino acids and a maximum of 3 missed cleavages was allowed. The FDR was set to 1% for peptide and protein identifications. Unique and razor peptides were considered for quantification. Retention times were recalibrated based on the built-in nonlinear time-rescaling algorithm. MS2 identifications were transferred between runs with the "Match between runs" option for biological replicates, in which the maximal retention time window was set to 0.7 min. We detected four phosphorylated tryptic peptides from three of the overexpressed microproteins (RP11-12K22.1-MP, JHDM1D-AS1-MP and THAP7-uORF- MP). Two of these peptides contained phosphorylation motifs (Table S3)."

Gene ontology analysis

Gene ontology (GO)[113] enrichment on small peptide and microprotein interactomes identified with PRISMA was performed with gProfiler2 v0.2.0,[88] with default parameters. As a custom background, we used all identified proteins in the respective PRISMA screen.

Co-localization analysis of PVT1-MP with SRFS2 and SRSF6

Synthetic gene fragments containing the codon-optimized coding sequence of PVT1-MP with a C-terminal V5-tag was synthesized and cloned into a customized plasmid for mammalian expression by Genewiz Europe (Leipzig, Germany; construct available upon request). Overexpression plasmids for SRSF2-FLAG and SRSF6-BirA-Myc-His were kindly provided by M. Gotthardt, MDC (constructs available upon request). PVT1-MP-V5 was co-overexpressed in HeLa cells with SRSF2-FLAG and SRSF6-BirA-Myc-His in equimolar ratios, respectively. Briefly, human HeLa cells (35 000) were grown on 8-well chamber slides for 24 h and transfected with the respective plasmids using Lipofectamine 3000 according to manufacturer's instructions. 24 h post transfection cells were fixed with 4% paraformaldehyde (PFA) for 10 min at room temperature (RT) and washed three times with ice cold phosphate-buffered saline (PBS). Cells were permeabilized and blocked for 1 h with 2.5% bovine albumin serum (BSA), 10% anti-goat serum (NGS) and 0.1% Triton X in PBS. After washing the cells, overexpressed PVT1-MP-V5 was stained with anti-V5 rabbit monoclonal antibody (1:500 in PBS with 5% BSA, #13202, Cell Signaling Technology), SRSF2-FLAG with anti-FLAG mouse monoclonal antibody (1:500 in PBS with 5% BSA, F1804, Sigma Aldrich), and SRSF6-BirA-Myc-His with anti-BirA chicken polyclonal antibody (1:500 in PBS with 5% BSA, BID-CP-100, BioFront Technologies) for 2 h at 4 °C. Afterwards cells were washed and incubated with fluorochrome-labeled secondary antibodies (1:500 in PBS with 5% BSA, Alexa Fluor 488 anti-mouse, Alexa Fluor 488 anti-chicken and Alexa Fluor 594 anti-rabbit (Invitrogen, Carlsbad, CA, USA) for 1 h at RT. Cells were washed again and stained with 4-6-diamidino-2-phenylindole (NucBlue Fixed Cell ReadyProbes Reagent, R37606, Thermo Fisher) for 5 minutes at RT. Images were taken with a LEICA SP8 confocal microscope using a 63x objective and analyzed using ImageJ (v1.53c).[108]

Proximity ligation assay

In situ proximity ligation assay (PLA) was performed to corroborate the interaction between PVT1-MP and SRSF2 using the Duolink® In Situ Proximity Ligation Assay Starter Kit (Red, Mouse/Rabbit, DUO92101-1KT, Sigma-Aldrich) according to manufacturer's instructions. V5-tagged PVT1-MP and FLAG-tagged SRSF2 were co-overexpressed in HeLa cells as described above for co-localization experiments. Following fixation with 4% paraformaldehyde (PFA) for 10 min at RT, cells were permeabilized in 0.1% Triton X in PBS for 1 h at RT, and blocked in Duolink® blocking solution for 1 h at 37 °C. PVT1-MP-V5 and SRSF2-FLAG were stained overnight at 4 °C with anti-V5 rabbit monoclonal antibody (1:500 in Duolink® antibody diluent, #13202, Cell Signaling Technology) and with anti-FLAG mouse monoclonal antibody (1:500 in Duolink® antibody diluent, F1804, Sigma Aldrich), respectively. Cells were washed twice for 5 minutes in 1x Duolink® Wash Buffer A at RT and incubated with PLUS and MINUS PLA probes (1:5 in the Duolink® Antibody Diluent) for 1 h at 37 °C. After washing cells 3x for 5 min in 1x Duolink® Wash Buffer A at RT, cells were incubated for 30 min at 37 °C with Duolink® ligase in 1x ligation buffer and washed again in 1x Duolink® Wash Buffer A at RT. For amplification, cells were incubated with Duolink® polymerase (1:80) in 1x amplification buffer for 100 min at 37 °C. Cells were washed 2x 10 min in 1x Duolink® Wash Buffer B, 1x 1 min in 0.1x Duolink® Wash Buffer B, and stained with 4-6-diamidino-2-phenylindole (NucBlue Fixed Cell ReadyProbes Reagent, R37606, Thermo Fisher) for 5 minutes at RT. As negative controls, transfected cells were stained with only anti-V5 rabbit monoclonal antibody, only anti-FLAG mouse monoclonal antibody, or no primary antibodies and PLA probes. Additionally, untransfected cells were stained with both primary antibodies and PLA probes. Images were taken with a LEICA SP8 confocal microscope using a 63x objective and analyzed using ImageJ (v1.53c).[108]

Generation of LINC01128-MP knock-out cells and RNA-seq analysis

A HeLa LINC01128-MP knock-out (KO) cell pool was generated by Synthego Inc. (Redwood City, CA) using CRISPR/Cas9 with GAUCCAAGGCAGGCACUCAA as guide RNA targeting the N-terminal region of the LINC01128-MP encoding sORF. Non-homologous end joining (NHEJ) following the Cas9-mediated double-strand break led to insertions and deletions (indels) that cause premature STOP codons and ultimately a disruption of the microprotein-encoding sORF (Figure S3F).

At passage four, RNA-sequencing was performed for triplicates of wild type (WT) cells and KO cell pools to assess i) the proportion of indels within the targeted cell pool and ii) the potential impact the introduced indels have on the expression of *LINC01128* transcripts. RNA-seq data was analyzed by trimming adapters and mapping each sample to the human genome (hg38, Ensembl v.98) using STAR v2.5.2b[82] with a maximum of 4 mismatches.

Our analyses revealed that 93.7% of all reads mapped to sequences carrying different indels that all lead to a frameshift resulting in premature STOP codons and ultimately disruption of the sORF encoding LINC01128-MP, while only 6.3% of reads mapped to the wild type sequence. A single adenine insertion was most common within the KO cell pools (69.4%), while a single guanine deletion, a 14 bp deletion and a 13 bp deletion contributed with 9%, 9.5% and 5.7%, respectively (Figure S3G). From these data, we can estimate that at least 87.4% of cells carry a mutation on both alleles.

Differential RNA-seq expression analysis was performed using DESeq2 v1.26.0.[89] Differentially expressed genes were selected based on adjusted p-values ($p < 0.05$) and log fold changes ($logFC < -0.18$ or $logFC > 0.18$) and showed that *LINC01128* transcript expression was not significantly altered through the introduced indels in comparison to wild type cells (Figures 3L and S3I).

The role of LINC01128-MP in transferrin endocytosis

For studying the uptake of fluorescently labeled transferrin-Alexa 647 in HeLa WT and HeLa LINCO1128-MP KO cells, cells from the same batch of cells that had been analyzed by RNA-seq (see above) were washed in PBS and incubated in DMEM without FBS for 30 min at 37°C. Subsequently, 5 μg/mL transferrin-Alexa 647 in DMEM were added to the cells for another 10 min at 37°C. Cells were washed several times in PBS and fixed in 4% PFA for 8 min on ice. Standard immunocytochemical analysis was carried out by incubation of cells with primary mouse anti-EEA1 antibody (1:100; BD Transduction Laboratories). Bound primary antibody was visualized using secondary antiserum conjugated with Alexa Fluor 555 (1:500; Invitrogen). Alexa 647-conjugated transferrin was purchased from Invitrogen (T23366). Nuclei were counterstained with DAPI (1:8000; Roche). Image acquisitions were carried out with a Leica TCS SP8 confocal microscope using a 63x PL APO CS2 oil immersion objective (NA 1.4). Manders' coefficient tM1 was determined with the Coloc 2 plugin from ImageJ/Fiji. Student's t-test was applied using Graph Pad Prism 7. Three independent experiments were performed. Per experiment and cell line, an average of 30 cells were analyzed for quantification.

Detection of sORFs$_{3\text{-}15\ aa}$ in human tissues

The first GENCODE annotation set of Ribo-seq ORFs only included sequences with a minimum length of 16 amino acids.[2] To identify very small novel ORFs translated in human tissues, we applied our previously published approach to detect actively translated ORFs in human left ventricular heart tissue (European Genome-Phenome Archive (EGA) accession code EGAS00001003263).[22] We decided to use this dataset as discovery tissue due to the high sample size (n = 80) and the high average codon periodicity (average of 85.4% in-frame reads for read lengths of 29 base pairs). We modified our original method for ORF detection[22] and removed the minimum length cutoff for ORF assignment, leading to the identification of 287 new translated ORFs starting with ATG and with a size of 15 amino acids or shorter (denoted sORFs$_{3\text{-}15\ aa}$). 221 of these ORFs met our translation rate cutoff, which required high levels of translation (minimum number of 290 raw P-sites and a minimum of 70% in-frame P-sites), resulting in their selection for further analysis (Table S4). We next inspected the translation of these 221 sORFs$_{3\text{-}15\ aa}$ in other human tissues by analyzing four publicly available ribosome profiling datasets corresponding to kidney, (n = 6, EGA accession code EGAS00001003263[22]), liver (n = 7, EGA accession code EGAS00001003263[22] and ArrayExpress accession code E-MTAB-7247[23]), brain (n = 3, ArrayExpress accession code E-MTAB-7247[23]) and testis (n = 3, ArrayExpress accession code E-MTAB-7247[23]).

Conservation of sORFs$_{3\text{-}15\ aa}$ in mammalian tissues

We defined the levels of conservation of sORF$_{3\text{-}15\ aa}$ by extending our analysis of conservation of sORF *structures* (see STAR Methods section 'conservation of sORF structures') to include non-primate mammals and three vertebrates, including four additional branches: euarchontoglires (~95 Mya), boreoeutheria (~102 Mya), placentalia (~107 Mya), mammalia (~225 Mya) and vertebrata (>300 Mya).

For comparison, we inspected the translation of 183 sORFs$_{3\text{-}15\ aa}$ with conserved structures in rodents. To this end, we retrieved datasets corresponding to mouse heart (n = 6, European Nucleotide Archive (ENA) accession code PRJEB29208[22]), mouse liver (n = 3, ArrayExpress accession code E-MTAB-7247[23]), mouse brain (n = 3, ArrayExpress accession code E-MTAB-7247[23]), mouse testis (n = 3, ArrayExpress accession code E-MTAB-7247[23]), rat heart (n = 30, ENA accession code PRJEB38096[50]) and rat liver (n = 30, ENA accession code PRJEB38096[50]). We additionally retrieved chicken brain Ribo-seq samples (n = 3, ArrayExpress accession code E-MTAB-7247[23]) to inspect the translation of USP10-uORF, an sORF$_{3\text{-}15\ aa}$ with conserved structure and length in mammals and birds. Ribo-seq datasets were filtered and mapped following a similar approach as for the mammalian Ribo-seq datasets (details in STAR Methods section 'expression and translation of sORFs in mammals'). Using the mapped data from the described tissues, we called translated ORFs per sample running ORFquant 1.00[46]. Next, we pooled the datasets by tissue and we extracted P-site counts with RiboseQC.[90] In-frame P-site counts were quantified for each annotated CDS and sORF$_{3\text{-}15\ aa}$. Finally, raw P-site counts were subjected to a normalization procedure (estimateSizeFactorsForMatrix; DESeq2 v1.26.0[89]) and divided by the total number of codons in each sequence. Additionally, we calculated the PhyloCSF[80] scores with default parameters of all 221 sORFs$_{3\text{-}15\ aa}$ (Figure S4E). PhyloCSF scores were calculated across the retrieved multiple alignments for primates and for

mammals. We found that a total of 30 (13.57%) and 44 sORFs$_{3\text{-}15\ aa}$ (19.91%) have positive PhyloCSF scores in primates and mammals. It is important to note that PhyloCSF performs better for sequences >30 nucleotides and can fail to determine the constraints of very short sequences,[80,114] which is why the determined PhyloCSF results can not be 100% reliable.

Effect of genomic location and length in the conservation of sORF structures

Compared to sORFs$_{3\text{-}15\ aa}$, cataloged sORFs (Ribo-seq ORFs, longer than 15 aa) contained a higher proportion of lncORFs (30% vs 8%), and a lower proportion of uORFs (42% vs 92%). Therefore, we inspected whether this difference in the biotype proportions had an effect on the observed patterns of conservation between cataloged sORFs and novel sORFs$_{3\text{-}15\ aa}$. We considered two main sORF biotypes: upstream ORFs (uORF, which contains 3,083 cataloged sORFs and 203 sORFs$_{3\text{-}15\ aa}$) and lncRNA ORFs (lncORF, which contains 2,208 cataloged sORFs and 17 sORFs$_{3\text{-}15\ aa}$). Next, we checked how many of the sORFs had conserved structures in five different species: mouse, rat, cow, horse and cat. An sORF structure was considered as conserved in the counterpart region of the compared species if the ATG translation initiation site (TIS) was present in the same position or within a window 6 nt down-stream of the human ATG position, and if $\geq$ 70% of the sequence did not contain stop codons truncating the ORF. For comparison, we randomly sampled non-translated ORF sequences from the same regions harboring translated sORFs (5′ UTRs for uORFs, lncRNA exons for lncORFs). We observed that both translated and non-translated lncRNA sequences were less highly conserved than 5′ UTR sequences (Figure S4G). UTR regions are enriched in promoters, secondary structures and binding motifs that are evolutionary constrained. Hence, we concluded that sORFs$_{3\text{-}15\ aa}$ are more likely to overlap these elements and be maintained over longer evolutionary times.

Secondly, we evaluated whether the intrinsic length differences between cataloged sORFs and sORFs$_{3\text{-}15\ aa}$ affected the different observed patterns of conservation between both groups. We consistently observed a continuum, in which the proportion of sORFs with conserved structures decreased with ORF length in the five analyzed species (Figure S4G). For the set of untranslated random sequences extracted from 5′ UTRs and lncRNAs, we also observed a similar negative trend between ORF length and conservation of ORF structures. This indicates that, even if not translated, disabling substitutions in very short ORFs (< 15 aa) are less likely to occur across evolutionary time.

Additional evidences of sORF$_{3\text{-}15\ aa}$ translation

We alternatively validated the translation of the set of 221 sORFs$_{3\text{-}15\ aa}$ in the same discovery set of 80 human left ventricular tissue Ribo-seq samples by running PRICE v1.0.3b.[47] This method uses an expectation–maximization algorithm to compute probabilistic inferences of codon activities. sORFs$_{3\text{-}15\ aa}$ with a p-value < 0.05 were defined as translated. Moreover, we retrieved a public resource containing the relative probabilities of each human transcript position to contain a translation initiation site (TIS). These probabilities were calculated using TIS Transformer,[48] a deep learning model based on information embedded in processed transcript sequences. Per each transcript containing a sORF$_{3\text{-}15\ aa}$, we extracted and ranked the probabilities of all possible ATG triplets in the transcript sequence.

Detection of sORF-translated peptides$_{3\text{-}15\ aa}$ in public mass spectrometry data

We collected protein expression evidence for translated sORFs$_{3\text{-}15\ aa}$ from previously published datasets.[6,51,52,54,55] Therefore, we re-analyzed the raw spectra using MaxQuant v1.6.10[56] with settings indicated in the respective dataset, added a false discovery rate (FDR) filter of <0.01 using the reverse-sequence based target decoy approach implemented in MaxQuant,[56] and disabled the protein FDR filter, as it was previously done for the identification of small proteins.[45,57] We added the peptide sequences together with the human UniProt database (HUMAN.2019-07) into the search space. We also searched the analyzed results of a published dataset of translated sORFs for our candidate peptides.[53] Moreover, we included the peptides$_{3\text{-}15\ aa}$ in the search database of the recent Human HLA 2022-09 PeptideAtlas build, in which the 51 million MS/MS spectra from 49 immunopeptide datasets deposited to ProteomeXchange[115] were processed with the PeptideAtlas build pipeline.[29,58] The reference database used was the comprehensive THISP level 3 database[116] plus the candidate peptide sequences. Processing was performed with MSFragger[91] and the Trans-Proteomic Pipeline[58] using a non-specific (no protease) search strategy and other parameters as appropriate for each of the 49 datasets. The Human HLA 2022-09 PeptideAtlas build contains 15 million peptide-spectrum matches (PSMs), among which 794 PSMs mapped to 16 of the short peptide sequences described herein. This PeptideAtlas build also included two of the individually analyzed studies.[6,51,52,54,55] All peptides detected in the respective datasets can be found in Table S4. Of note, only 116/221 peptides$_{3\text{-}15\ aa}$ are theoretically detectable with standard shotgun MS, because they produce tryptic peptides above six aa after enzymatic digest. To ensure a proper identification, peptides additionally require to be unique within the digested proteome, which further reduces the number of peptides able to be detected. 193/221 peptides are theoretically detectable in immunopeptidomics, since no digestion is required. To ensure the validity of peptide identifications, each identification was required to be unique within the proteome. This was assessed with the tool Proteomapper v.1.5[92] (http://www.peptideatlas.org/map/), which maps input sequences to the proteome, in order to exclude that the identified peptides stem from fragments of longer proteins. Our analysis showed that all identified peptides are either unique within the proteome or, if present within other proteins, cannot be cleaved into the identified peptides because of lacking tryptic restriction sites. However, four of the identified peptides could theoretically stem from semi-tryptic digestion of canonical proteins and four might be derived from longer alternative sORF isoforms encoded by the same gene that had been detected in previous ribosome profiling studies[2] (Table S4).

PRM proteomics

The parallel reaction monitoring (PRM) analysis was performed similarly to the targeted mass spectrometry approach carried out by us previously,[22] except for some modifications. To select appropriate tryptic and LysC-proteolytic signature peptides, we first digested candidate microproteins and peptides$_{3\text{-}15\ aa}$ *in silico* with i) trypsin and ii) LysC using the online tool MS-Digest (http://prospector.ucsf.edu). We selected tryptic or LysC-proteolytic peptides that were unique across the human proteome digested with the respective enzyme, had a minimum length of six aa and fell into a mass to charge (m/z) range of 10 - 1850 m/z. Selected peptides were purchased as synthetic peptides of crude quality (JPT Inc., Berlin, Germany), which were resuspended in 20% acetonitrile (100 mM ammonium bicarbonate) and measured (1 pmol per peptide) on a Q-Exactive HF-X mass spectrometer (Thermo Fisher Scientific) using data dependent acquisition mode (DDA) with a mass resolution of 60,000 for the MS scans and 15,000 for the MS/MS scans, considering precursor ions with charge state 1 to 6. Precursor fragmentation efficiency was evaluated using normalized collision energies (NCE) ranging from 25 to 35. The recorded spectra were analyzed using MaxQuant v1.6.3.4[56] applying a custom-made database containing the predicted sequences, with carbamidomethylation of cysteines as fixed and oxidation of methionines as variable modification. Based on the observed precursor signal, retention time, charge state and MS/MS fragment information, a library based PRM method was developed using the Skyline software package v3.6[93] with the following settings: precursor charge state 1 to 4; ion charge state 1 and 2; ion types y, p, b, a, z; auto-selection of matching transitions enabled; ion match tolerance = 0.05 m/z; method match tolerance = 0.055 m/z. For each candidate the most abundant variant together with the corresponding fragment ions (five or more) were selected. In total, we included 42 tryptic and 16 LysC precursor targets for 24 microproteins, and 77 tryptic and 193 LysC precursor targets for 149 peptides$_{3\text{-}15\ aa}$ (Table S5). A subset of precursor targets did not generate fragmentation patterns suitable for PRM analysis and were therefore excluded. The final set consisted of 39 tryptic and 9 LysC precursor targets for 24 microproteins, and 73 tryptic and 155 LysC precursor targets for 129 peptides$_{3\text{-}15\ aa}$. As positive controls, proteotypic peptides from high abundant housekeeping genes (tryptic peptides: GAPDH, ACTA, and HIST1H2; LysC peptides: GAPDH and H2BC1) and sCDS were included (tryptic peptides: PLN and MIEF1-MP; lysC peptides: NDUFB3, MTLN, MPRL33, and MIEF1-MP). Based on their retention time profile, targets were split into two (tryptic peptides) and three (LysC peptides) PRM inclusion lists. Parameters for the positive controls were added to each list. Analytical PRM measurements were performed on a High Performance Liquid Chromatography (HPLC) system (ThermoScientific) using a 98 min gradient of increasing Buffer B concentration (from 2% to 60%, 250 nl/min flow rate) coupled to an Q-Exactive HF-X mass spectrometer (Thermo Fisher Scientific). Instrument parameters were set to 30,000 resolution with 2e5 AGC target value, 100 ms maximum ion injection time, 30 min retention time window and adjusted normalized collision energy (NCE) values for each target.

Analytical PRM measurements were performed on human tissue and cell line samples. Therefore, pulverized human heart tissue of five patients and three biological replicates of PBS-washed cell pellets of HEK239T, HeLa and K562 cells were resuspended in lysis buffer (1% weight per volume (w/v) sodium deoxycholate (SDC), 10 mM DTT, 40 mM chloroacetamide (CAA), 1 mM ethylenediaminetetraacetic acid (EDTA), 100 mM Tris, pH 8.5) and boiled for 10 min at 95°C. After cooldown, samples were incubated with Benzonase (Merck Germany) to digest nucleic acids. For tryptic peptide samples, 10 μg of protein extract was digested with 0.2 μg sequence grade endopeptidase LysC (Wako, Osaka, Japan) and 0.2 μg sequence grade trypsin (Promega). LysC peptide samples were obtained by digesting 10 μg of protein extract with 0.2 μg sequence grade endopeptidase LysC only (Wako, Osaka, Japan). All digests were performed at 37°C for 16 hours. The digest was stopped by acidifying each sample to pH < 2.5 by adding 10% trifluoroacetic acid solution. After centrifugation to pellet insoluble material (14,000 rpm, 10 min), the peptides were extracted and desalted using the StageTip protocol.[109] Peptide samples were eluted from StageTips (80% acetonitrile, 0.1% formic acid), dried down, resolved in sample buffer (3% acetonitrile and 0.1% formic acid) and analyzed on the mass spectrometer as described above. For heart tissue samples, two technical replicates for each biological replicate were performed. Cell line samples were analyzed by one analytical run per replicate.

PRM data analysis was carried out using the Skyline software package v21.02.[93] Analytical runs of synthetic peptide mixtures as well as the internal library-based fragment ranking were used to manually confirm the peak assignment. Peptides detected in at least two biological replicates with a dot product of ≥ 0.7 were considered robust identifications. For these peptides, also peaks with a dot product ≥ 0.6 were reported in the remaining samples. For peaks passing the quality filter, the total peak area, retention time and dot product values were exported and are available for all technical and biological replicates (Table S5). We were able to detect four out of the six sCDS. The control proteins GAPDH, ACTA, HIST1H2, and H2BC1 were robustly identified in all replicates of the five heart samples and the three cell lines. We detected signature peptides for two microproteins and for 18 peptides$_{3\text{-}15\ aa}$. Of the latter 18, two peptides could also stem from semi-tryptic digestion of canonical proteins and four might be also derived from longer alternative sORF isoforms encoded by the same gene (Table S4).

PRISMA of peptides translated from sORFs$_{3\text{-}15\ aa}$

Setup, Preparation, LC-MS/MS & Data analysis

The PRISMA approach described above was used for the peptide dataset of 221 candidate peptides with minor adaptations. We decided to use the same PRISMA controls as for the microproteins after analysis of amino acid frequencies (Figure S2C): four peptides derived from SOS1 and GLUT1 (SOS1wt, SOS1mt, GLUT1wt, GLUT1mt) that had been previously investigated.[18,32] All 221 candidate peptides as well as the four PRISMA controls were spot-synthesized onto a cellulose membrane (JPT Peptide Technologies Inc., Berlin, Germany). We were able to synthesize each peptide in full length due to their short size. The assay was performed in

triplicates, *i.e.*, three membranes with identical peptides. The sequences of all spotted peptides were identical to all identified peptides and are listed in Table S4 together with the sequences of the four control peptides. Preparation for MS (*i.e.*, incubation of the membrane with HEK239T protein lysate, punching out the peptide spots, digesting the peptide spots with trypsin and LysC, preparation of the StageTips), LC-MS/MS (*i.e.*, elution of the peptides, separation of the peptides on a HPLC system, ionization and analysis of the peptides on an Orbitrap Fusion instrument with above settings), as well as raw data analysis and filtering steps for identification of PPIs were performed as described in the above PRISMA screen for microproteins.

Quality control by replicate measurement assessment

No batch bias was identified between the replicate membranes (Figures S5A and S5B) and the triplicates of the same peptide correlated with a mean correlation coefficient (Pearson's R) of 0.82, indicating good data reproducibility (Figure S5C). Six samples were excluded at this step because their correlation or number of interactors deviated more than two standard deviations from the other two samples. Taken together, a total of 3,784 unique binding proteins (including 64 bait peptides) were identified, with a range of 191 to 2,711 proteins binding to the individual peptides (mean identifications per peptide: 1,376 proteins, Figure S5D). The wide range of binding proteins could be expected due to the exploratory nature of the screen: some peptides might not be biologically relevant and will therefore not display any binding capacity, which in turn adds greater weight to the ones that do bind other proteins specifically. In order to distinguish between specific (transient) interactions and unspecific background binding, each triplicate was analyzed with regard to all other peptide pull-downs (used as background) via label-free quantification.

Quality control by evaluation of assay control peptides

We used the known interaction partners of GLUT1 and SOS1 to determine the appropriate significance cutoff,[18,32] resulting in a cutoff of c=6 and x_0=2.8, equal to two standard deviations (Figures S5F and S5G). The expected binding behavior of GLUT1 and SOS1 is described above (PRISMA "Quality control by evaluation of assay control peptides"). For GLUT1, reassuringly, we find all known binding partners enriched in the mutant but not in the wild type peptide. For SOS1, we find all previously reported binding partners in the wild type, and only 3/9 in the mutant. Additionally, we find four previously unreported proteins with SH3 domains in the wild type but not in the mutant. Our very specific assay controls demonstrate the ability to detect previously reported, biologically relevant proteins, as well as its specificity to the spotted peptide sequence. In total, we identify between zero to 212 proteins per peptide (mean identification of 11 proteins per peptide, Figure S5H). We found that the shortest peptides (below 6 aa) have slightly lower numbers of interaction partners overall.

Quality control based on bait identification for PRISMA of peptides$_{3\text{-}15\ aa}$

Similar to what was described for PRISMA, we assessed if the spotted peptides (baits) were identified and enriched in the expected samples (Figure S5E). We detected 64 baits in total. It was to be expected that not all bait peptides could be detected, as the short length of the peptides reduces the possibility to produce tryptic peptides suitable for MS. 29/64 baits were found exclusively in expected samples. 28 were found in multiple samples but with higher mean LFQ intensity across the three triplicates in the expected sample compared to the unexpected sample. One bait was found exclusively in an unexpected sample, but it was only identified "by matching" and not "by MS/MS" and also had a relatively low LFQ intensity. As explained above, the high amount of peptide synthesized on the membrane should lead to a robust identification by peptide-spectrum matches and hits identified only "by matching" are more likely false positives. Six baits were not significantly enriched in any sample.

Peptide properties

Peptide hydrophobicity values were calculated using the Krokhin model.[117] For prediction of the peptides' MHC presentation, we used the tool NetMHCpan-4.1[95] which predicts binding of peptides to MHC molecules of known sequence using a neural network. We selected the 12 indicated MHC supertype representatives for binding analysis. In total, 105 peptides were reported to be presented on one or more MHC supertype representatives. The results, indicating for how many MHC supertype representatives a peptide is predicted to be presented, are listed in Table S4.

Analysis of CORUM complexes in interactomes of peptides$_{3\text{-}15\ aa}$

CORUM complexes[118] were downloaded from http://mips.helmholtz-muenchen.de/corum/ (Human_CORUM_coreComplexes_2018-09-03_symbol.gmt). We annotated a complex when at least two proteins of a CORUM complex were present in the interactome of a specific peptide.

Luciferase assay for ribosome-binding peptides

We identified 16 peptides that clustered together in the global hierarchical clustering, indicating that their interactomes were very similar. For the reporter assay, we selected five out of 16 peptides encoded by sORFs$_{3\text{-}15\ aa}$ within 5′ UTRs. We created two mutants for each of the five candidates: one with a mutated start codon (ATG > ACG) that impedes translation of the uORF, and one where we mutated charged arginines to non-charged, inert alanines. We created this second control (i.e., the charge mutant) in order to assess the impact of the peptide's sequence and/or charge on its effect on downstream (luciferase) translation. The respective 5′ UTRs were synthetized and cloned into the backbone in front of the Renilla luciferase using the NheI restriction site (psiCHECK-2, constructs available upon request). We then confirmed the plasmid sequences using Sanger Sequencing. For the reporter assay (Dual Glo Luciferase Assay System, Promega, E2920), human HeLa cells were grown in a white 96-well plate with a transparent bottom. All plasmids including internal controls were transfected using LipoJet Reagent. The assay was performed 48 hours after transfection, according

to the manufacturer's manual. 50 μL of Dual Glo Luciferase Agent was added to the cells, which were then incubated for 30-40 min at RT. The cells were checked for complete lysis under a microscope to ensure proper read out of the luminescence signal. The level of luminescence was measured using the fluorescent plate reader Safire2 (Tecan) and the program Magellan with a luminescence integration time of 100 ms. Immediately after the measurement, 50 μL of the Stop & Glo Reagent was added. The mixture was incubated for exactly the same time span as after the addition of the first reagent. After incubation, the luminescence levels were measured again using the same plate reader, program and settings. We performed four biological replicates with three to four technical replicates each. The resulting luminescence levels were analyzed in Excel and plotted in R. First, the background of untransfected cells was subtracted from the values of transfected samples. Then, the ratio of Renilla luciferase to Firefly luciferase was calculated, followed by the calculation of the fold changes in respect to the ATG mutant for each biological replicate individually. An ANOVA was performed for statistical analysis.

Endocytosis assay for adaptor-protein-binding peptides$_{3\text{-}15\ aa}$

Experimental setup

The impact of peptides on receptor-mediated endocytosis was tested in an endocytosis assay in cultured BN16 cells expressing the endocytic receptor low-density lipoprotein receptor-related protein 2 (LRP2/megalin).[119] To do so, we ordered synthetic peptides including three candidate peptides and one control peptide from Pepscan (Lelystad, Netherlands) with a TAT-sequence at the N-terminus (GRKKRRQRRRPQ) to facilitate the entry into the cytoplasm of the cells, a linker sequence (Ahx), and a fluorescent 5(6)-FAM to be able to visualize the internalized peptides (FAM-{Ahx}-TAT-peptide). We decided to use a TAT-peptide for shuttling the peptides into the cells, as we believed that it would not influence the results of the assay. The mode of cell entry of TAT is still not completely clear and likely depends on the type of cargo and target cell.[120,121] Endocytosis, macropinocytosis, but also direct membrane translocation or inverted micelle formation are mechanisms proposed for TAT delivery into cells.[121] To additionally assure that the TAT has no impact on the assay results, we added a control peptide into the assay that did not bind any endocytosis-related proteins in the PRISMA screen (PPARD-uORF-peptide). If the TAT itself would affect endocytosis levels more than the delivered peptide, we assume that all tested TAT-peptides would produce similar results.

Endocytosis assay and analysis

For studying the uptake of fluorescently labeled receptor-associated protein (RAP), the protein was recombinantly expressed and purified to homogeneity from E. coli and labeled with Alexa Fluor 594 (Alexa Fluor 594 Protein Labeling Kit, A10239, Thermo Fisher). BN16 cells were seeded at a density of 50k cells/well (24-well-plate) in DMEM supplemented with FBS as described above. The next day, cells were incubated in DMEM without FBS for 30 min at 37°C, followed by pre-treatment with 50μM peptides, DMSO or dynasore in DMSO for 60 min at 37°C. Then, 10 μg/ml RAP was added to the cell supernatant for another 60 min at 37°C. At this point, images of the cells were acquired with a fluorescence microscope directly using the cell plates. For the fluorescence quantification, cells were washed several times in PBS and lysed in RIPA fluorescence lysis buffer (50 mM Tris pH 7.4, 150 mM NaCl, 1% NP40, protease inhibitor (cOmplete, Merck)) for one hour on ice. Lysates were cleared by centrifugation at 12,000 rpm for 10 min at 4°C. Uptake of RAP by LRP2 was then assessed by measuring the fluorescence of RAP in cell lysates, which was measured in triplicates in black 96-well-plates (655079, Greiner Bio-One) using a fluorescence reader (Tecan). The results were normalized to total protein content. Values of blank samples (without RAP) were subtracted from all measurements. Values of the various experimental conditions are displayed as normalized to the condition with RAP only (set to 100%).

Immunofluorescence of peptides$_{3\text{-}15\ aa}$

Human HeLa cells were grown in ibidi chambers for 24 h. Cells were starved (*i.e.*, kept in DMEM medium without FCS) for 30 minutes and then treated with 50 μM of respective peptide for 1 h (sequences see "Endocytosis assay" above). Cells were washed 3x with PBS for 5 minutes each and subsequently fixed with 200 μL of ice cold 4% PFA per ibidi well for 10 minutes at RT. Afterwards, cells were washed on a shaker twice with ice cold PBS for 5 min each. Cells were stored in PBS at 4°C in the dark until imaging. For staining the mitochondria, cells were blocked for 1 h at RT in the dark using 3% BSA, 10% NGS, and 0.1% Triton X, and washed again three times for 5 minutes each. Cells were stained with an antibody against mitochondria (ATPIF1, 1:1000, ab110277, abcam) for 1h at RT or overnight. Afterwards, cells were washed three times in ice-cold PBS for 5 minutes each and incubated with the secondary antibody against mouse (1:500, Alexa 594 anti-mouse, Invitrogen) for 30 minutes at RT. Cells were washed again and stained with 4′,6-Diamidin-2-phenylindol (DAPI, NucBlue Fixed Cell ReadyProbes Reagent, R37606, Thermo Fisher) for 5 minutes at RT and stored at 4°C in the dark until visualization. Images were visualized with a Leica SP8 confocal microscope using a 63x objective and the corresponding software. Negative controls with only secondary antibody were used in order to avoid false positive results (autofluorescence). Images were analyzed using Fiji.[94]

QUANTIFICATION AND STATISTICAL ANALYSIS

The generation of figures and statistical analyses were performed using custom scripts and R v.3.6.1.[87] A detailed list of software used for data processing, quantification and analysis is stated in the respective STAR Methods sections and the key resources table. Statistical parameters such as the value of n, mean/median, standard deviation (SD) and significance level (including the type of statistical test) are reported in the STAR Methods, figures and/or in the figure legends. The values of "n" represent sample numbers of

CellPress
OPEN ACCESS

human or animal tissue (STAR Methods sections "experimental model and subject details" and "detection of sORFs3-15aa in mammalian tissues"), the number of sORFs (results text, Figures 1A, 1E, 1G, and S1), number of microproteins (Figure S2K), number of experiments (Figure 3K) and the number of sORFs$_{3\text{-}15\ aa}$ (Figures 4F and S4B), uORFs and lncORFs (Figure S4G). Statistical parameters used to indicate differential expression were derived from DESeq2 (STAR Methods: section "generation of LINC01128-MP knock-out cells and RNA-seq analysis"), or otherwise the type of statistical test (e.g., Mann-Whitney U test or t test) is annotated in the figure legend and indicated in the STAR Methods segment specific to each analysis. Unless stated otherwise, statistical analyses are two-sided tests performed using R. In the PRISMA analysis, samples were excluded that deviated more than two standard deviations away from its other replicates regarding number of sample identifications as well as samples whose correlation value was over two standard deviations away from the other correlations between replicates. False discovery rate (FDR) was estimated using the Benjamini & Hochberg method.

Molecular Cell, Volume *83*

Supplemental information

Evolutionary origins and interactomes of human, young microproteins and small peptides translated from short open reading frames

Clara-L. Sandmann, Jana F. Schulz, Jorge Ruiz-Orera, Marieluise Kirchner, Matthias Ziehm, Eleonora Adami, Maike Marczenke, Annabel Christ, Nina Liebe, Johannes Greiner, Aaron Schoenenberger, Michael B. Muecke, Ning Liang, Robert L. Moritz, Zhi Sun, Eric W. Deutsch, Michael Gotthardt, Jonathan M. Mudge, John R. Prensner, Thomas E. Willnow, Philipp Mertins, Sebastiaan van Heesch, and Norbert Hubner

Supplemental Figures and Legends

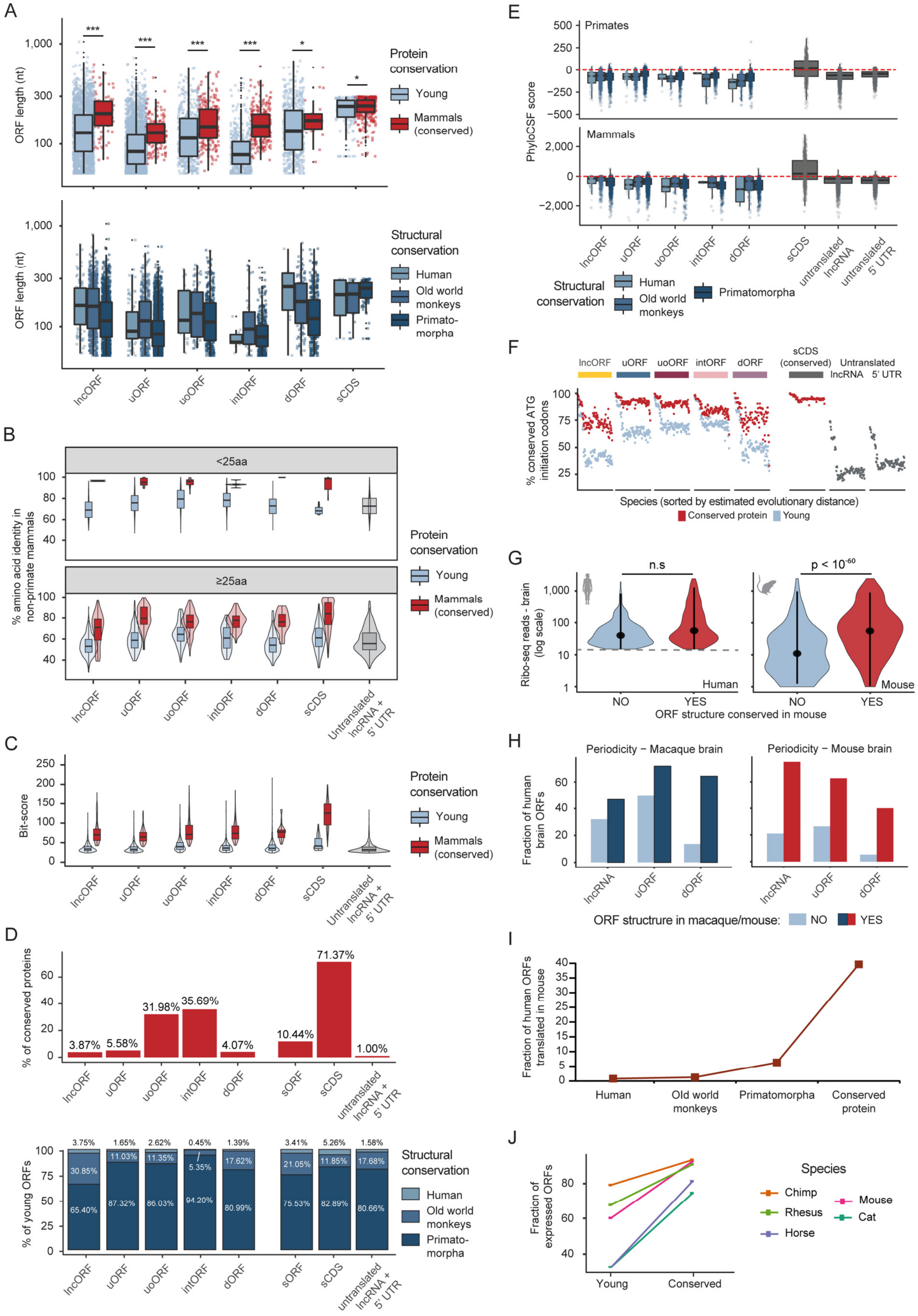

Figure S1. The majority of human sORFs are young and have emerged *de novo*, Related to Figure 1.

(A) Boxplots with length of sORFs (n = 7,264) and sCDS (n = 527) divided by ORF biotype and conservation of amino acid (aa) sequences (top) or ORF structures (bottom, only young ORFs (CS < 8)). Significance of difference was assessed by Wilcoxon test (*, $p < 0.05$; ***, $p < 10^{-5}$).

(B) Boxplots with percentages of average aa identity across non-primate mammals for sORF- and sCDS-encoded microproteins as well as untranslated controls (n = 4,982) divided by ORF biotype, length, and conservation of aa sequences.

(C) Boxplots with ORF alignment bit-scores calculated with BLASTP across the counterpart regions of non-primate mammals for 7,264 sORF- and 527 sCDS-encoded microproteins, as well as 4,982 untranslated RNA regions. ORFs are divided by biotype and conservation of aa sequences.

(D) (Top) Bar plots with the percentages of sORFs, sCDS and untranslated controls whose amino acid sequences are significantly conserved (CS ≥ 8). (Bottom) Bar plots with the percentages of young sORFs (n = 6,506), young sCDSs (n = 148 and untranslated controls (n = 4,982) by evolutionary age based on the conservation of ORF structures. Conserved proteins (CS ≥ 8) are not included.

(E) Boxplots with PhyloCSF scores calculated across all aligned primate (top) and mammalian (bottom) genomes, for young sORFs, sCDSs and untranslated controls. sORFs are divided by ORF biotype and conservation of ORF structures. The distribution of PhyloCSF scores in sORFs is not statistically higher than in untranslated regions (Wilcoxon one-sided signed rank test $\text{p-value}_{\text{primates}} = 1$, $\text{p-value}_{\text{mammals}} = 1$)

(F) Dot plots displaying the percentage of conserved ATG initiation codons in the non-human counterpart sequences of sORF, sCDS, and untranslated ORFs. ORFs are divided by biotype and conservation of aa sequences. ATG initiation codons from young sORFs are significantly more conserved than the ones from untranslated lncRNA and 5' UTR regions (Wilcoxon signed rank test, P-value = 1.45×10^{-21}).

(G) Violin plots with the numbers of mapped Ribo-seq reads (log-scale) of sORFs translated in the human brain (n = 830), by presence (light blue) or absence (red) of conservation in mouse. Mapped reads correspond to human brain (left) or mouse brain (right). Ribo-seq samples had three biological replicates each[1]. Statistical differences in the number of Ribo-seq reads were assessed by Wilcoxon signed rank test. Dot and vertical bar represent the median and the distribution of minimum to maximum values, limited to the most extreme data point that is no more than 1.5x interquantile range (IQR), respectively.

(H) Bar plots with the percentages of human brain translated sORFs with aligned counterpart regions displaying significant Ribo-seq periodicity biases in macaque (left) and mouse (right) brain. sORFs are divided by biotype and by presence (light blue) or absence (dark blue, red) of conservation in macaque or mouse. Upstream overlapping ORFs (uoORFs) and internal ORFs (intORFs) were not considered since the periodicity signal could be masked by translated coding sequences in alternative frames. Significance of periodicity bias was assessed by Binomial test ($p < 0.01$).

(I) Fraction of human sORFs translated in public mouse datasets[1,2] by estimated evolutionary age category.

(J) Fraction of young and conserved human sORFs expressed in chimpanzee, rhesus macaque, horse, mouse, and cat transcriptomes.

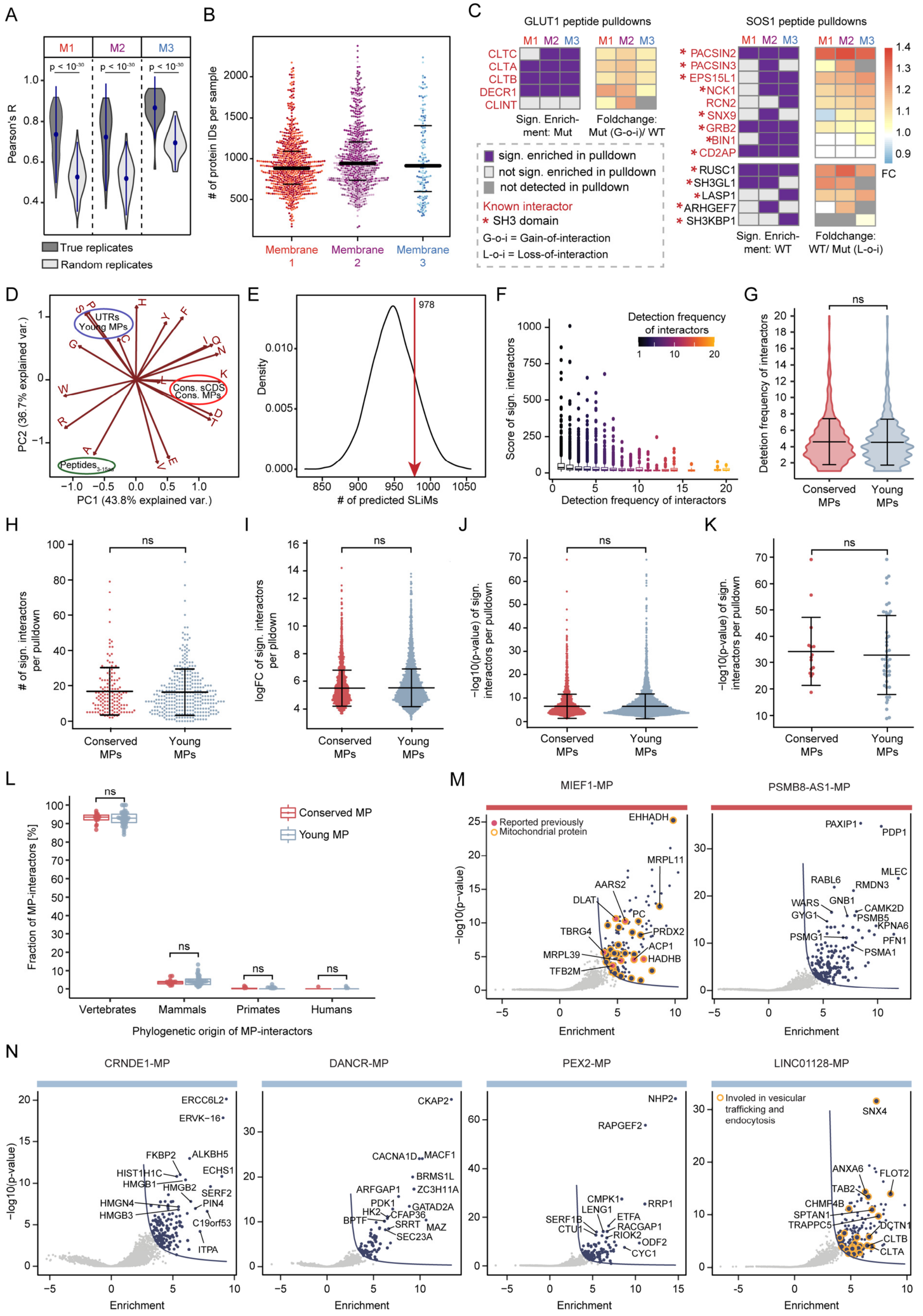

Figure S2. Interactome profiling of microproteins translated from young sORFs with PRISMA, Related to Figure 2.

(A) Pearson's correlation of true replicates is significantly higher than correlations of random samples for each membrane as assessed by Welch's t-test. Indicated are the mean ± 2 standard deviations.

(B) Distribution of total numbers of identified proteins per membrane. Dots are colored by replicates. The horizontal lines indicate 25, 50 and 75% quartiles, respectively.

(C) Left: four of the five known interactors of the GLUT1 gain-of-interaction (G-o-i) mutant peptide can consistently be detected across all three biologically distinct membranes M1, M2 and M3. Fold change (FC) of LFQ intensity of GLUT1 gain-of-interaction (G-o-i) versus wild type (WT) peptide: as expected, known interactors are enriched in G-o-i peptide pulldown (FC > 0) showing that depicted interactors bind preferentially to the mutant sequence in comparison to the wild type sequence. Right: up to eight of the nine known interactors as well as five novel SH3-domain-containing interactors of SOS1 wild type peptide can be detected across the three biologically distinct membranes M1, M2 and M3. Fold change of LFQ intensity of SOS1 WT versus Loss-of-interaction (L-o-i) mutant: as expected, most known interactors are enriched in the WT peptide (FC > 0) showing that depicted interactors bind preferentially to the WT sequence.

(D) Principal component analysis (PCA) of the amino acid frequencies of sequences included in the PRISMA dataset (45 young microproteins, 15 conserved microproteins, 221 small peptides), 271 arbitrary peptides derived from *in silico* translation of untranslated regions from 5' UTRs and lncRNAs, and 379 conserved annotated microproteins (sCDS). Frequencies were determined by calculating the number of occurrences of each amino acid per group, divided by the summed length of the sequences of each group. Methionines were excluded from the frequency calculations.

(E) Density plot with the distribution of the numbers of predicted SLiMs in 10,000 sampled sets of shuffled amino acid sequences for each of the 45 young microproteins included in the PRISMA screen. The number of SLiMs predicted in the true set of 45 young microproteins is depicted with a red arrow (978) and is not statistically different from the distribution of the number of SLiMs in shuffled sequences (Pearson's χ^2 test, p-value = 0.16).

(F) Boxplot of detection frequencies of microprotein interactors. The detection frequency of interactors describes in how many microprotein interactomes a single interactor was detected. The detection frequency decreases with the interaction score of the interactor (defined as product of p-value and fold change).

(G-K) Comparisons of **(G)** detection frequency of interactors, **(H)** number of interactors, **(I)** fold change of interactors, **(J)** p-value of interactors, and **(K)** p-value of top interactors per microprotein between conserved (n = 15) and young (n = 40) microproteins show no significant differences. Assessed by two-tailed student's t-test. Horizontal lines indicate the mean ± standard deviation.

(L) Phylogenetic origins of interactors of conserved and young microproteins. There is no significant difference when fractions of interactors per phylogenetic age are compared between young and conserved microproteins (Wilcoxon rank sum test, FDR adjusted > 0.05). For both, the majority of interactors (> 90%) originated in vertebrates.

(M) Volcano plots with interactome results of the conserved and annotated mitochondrial sCDS MIEF1-MP and the conserved microprotein PSMB8-AS1-MP (interactors from all tiles are summarized). Known interactors and novel mitochondrial interactors of MIEF1-MP are highlighted in pink and with an orange circle, respectively.

(N) Volcano plots with interactome results of the four evolutionarily young microproteins CRNDE1-MP, DANCR-MP, PEX2-MP and LINC01128-MP (interactors from all tiles are summarized). Interactors of LINC01128-MP that are involved in vesicular trafficking and/ or endocytosis are highlighted with an orange circle

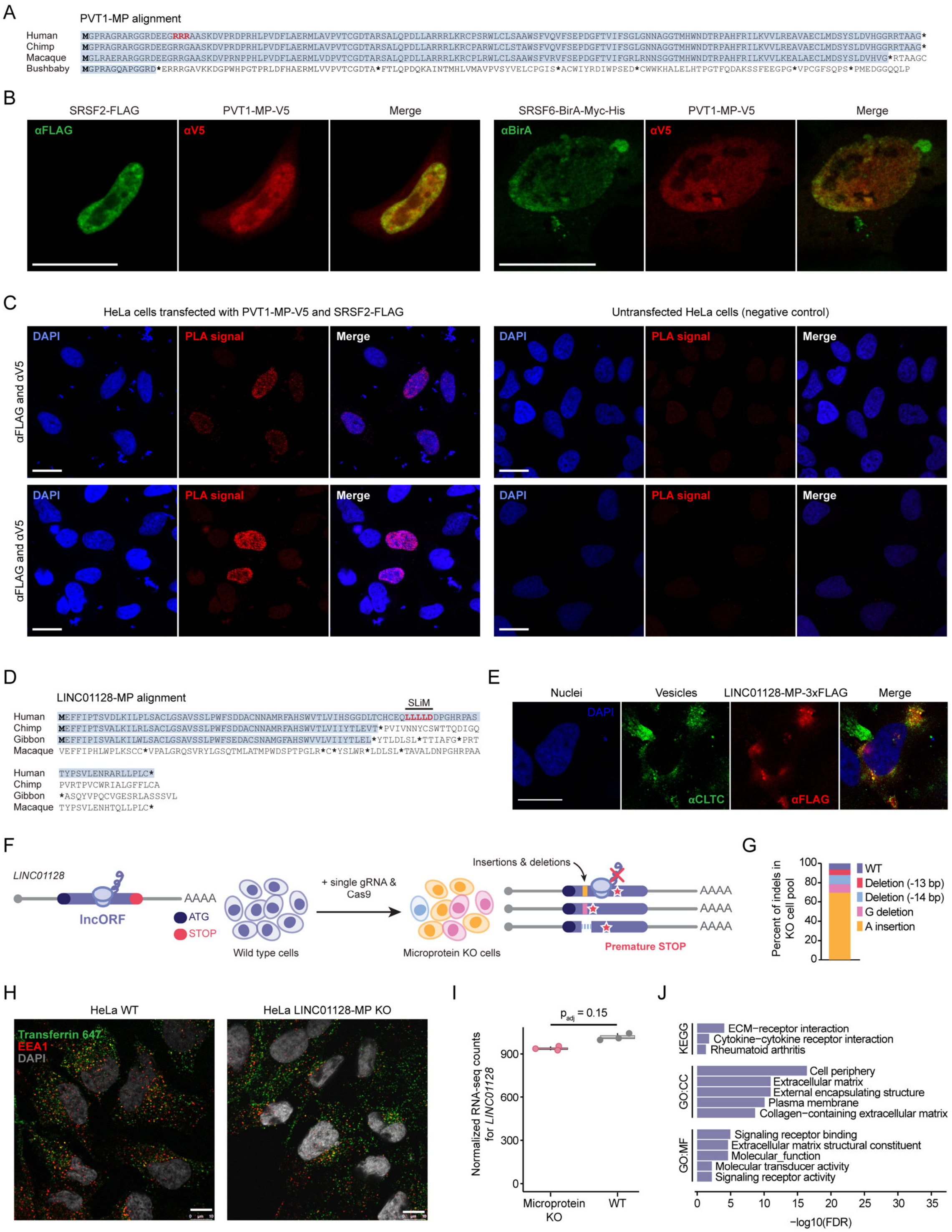

Figure S3. Short linear motifs (SLiMs) may drive microprotein-protein interactions, Related to Figure 3.
(A) Amino acid sequence alignment of the human microprotein PVT1-MP and its orthologous regions in three selected primate genomes. The stretch of three arginines that potentially mediate PVT1-MP interactions with splicing factors is highlighted in red. Intact open reading frame structures are highlighted in light blue.

(B) Partial co-localization of V5-tagged PVT1-MP with FLAG-tagged SRSF2 (left) and BirA-tagged SRSF6 (right) after overexpression in HeLa cells. PVT1-MP was stained with anti-V5 antibody, SRSF2 with anti-FLAG antibody and SRSF6 with anti-BirA antibody. Scale bars represent 20 µm.

(C) Proximity ligation assay (PLA) with anti-V5 and anti-FLAG antibodies in HeLa cells transfected with V5-tagged PVT1-MP and FLAG-tagged SRSF2 (left) and untransfected HeLa cells as negative control (right) to complement **Figure 3G**. Red fluorescent spots indicate PVT1-MP-V5 and SRSF2-FLAG interactions. Cell nuclei were stained with DAPI (blue). Scale bar represent 20 µm.

(D) Amino acid sequence alignment of the human microprotein LINC01128-MP and its orthologous regions in three selected primate genomes. Intact open reading frame structures are highlighted in light blue. The C-terminal region that carries the clathrin box motif (highlighted in red) is specific to humans.

(E) FLAG-tagged LINC01128-MP co-localizes with clathrin heavy chain protein (CLTC) after overexpression in HeLa cells. Single channel images to complement images in **Figure 3I**. Cell nuclei were stained with DAPI, CLTC with anti-CLTC antibody and LINC01128-MP with anti-FLAG antibody. Scale bar represents 20 µm.

(F) Schematic of knock-out strategy to disrupt the sORF encoding LINC01128-MP using CRISPR/Cas9 in HeLa cells. Non-homologous end joining (NHEJ) following the Cas9-mediated double-strand break leads to insertions and deletions (indels) that cause premature STOP codons and ultimately a disruption of the microprotein-encoding sORF. For RNA-seq and endocytosis experiments, a pool of cells carrying different indels was used.

(G) Proportion of indels within the CRISPR/Cas9 targeted cell pool based on RNA-seq data. The analysis was based on 76 RNA-seq reads covering the region of interest within *LINC01128*. Displayed is the average of three replicates.

(H) Representative images of fluorescently labeled transferrin (green) and EEA1 (red) detection in HeLa wild type and LINC01128-MP knock-out (KO) cells complementing **Figure 3J**. Cell nuclei were stained with DAPI (gray) and EEA1 with anti-EEA1 antibody. Scale bar represents 10 µm.

(I) Boxplots with DESeq2-normalized RNA-seq counts of *LINC01128* transcripts in HeLa wild type and LINC01128-MP-KO cells. Genome-wide corrected p-values are given.

(J) GO enrichment analysis of genes differentially expressed upon LINC01128-MP-KO in HeLa cells. The top five significant terms are plotted per group.

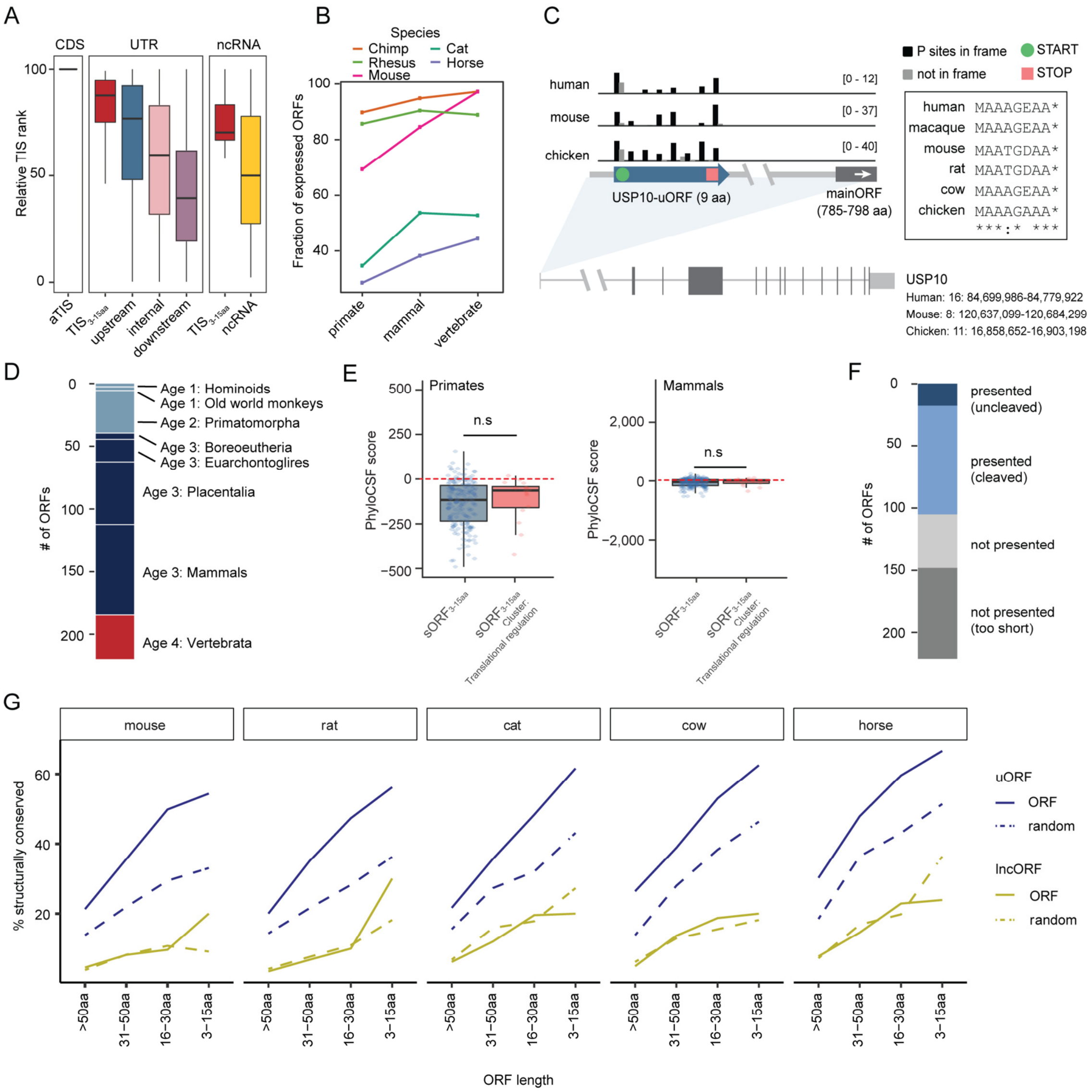

Figure S4. sORFs smaller than 16 aa (sORFs$_{3\text{-}15aa}$) are highly translated in multiple tissues and often conserved across mammals, Related to Figure 4.

(A) Results of all sORFs$_{3\text{-}15aa}$ analyzed with TIS (translation initiation site) transformer[3]. The y-axis represents the relative rank of the TIS compared to the rest of ATGs in the respective transcript. A rank of 100% means that the TIS is the ATG predicted with the highest probability. aTIS: annotated TIS. p-values calculated by Wilcoxon test, in UTRs: TIS$_{3\text{-}15aa}$ vs. upstream: $p = 1.29*10^{-9}$; TIS$_{3\text{-}15aa}$ vs. internal: $p = 9.37*10^{-37}$; TIS$_{3\text{-}15aa}$ vs. downstream: $p = 5.17*10^{-82}$; in ncRNAs: TIS$_{3\text{-}15aa}$ vs. ncRNA: $p = 0.04$.

(B) Fraction of human sORFs$_{3\text{-}15aa}$ (n = 221) expressed in chimpanzee, rhesus macaque, horse, mouse, and cat transcriptomes by conservation category.

(C) Genomic view and sequence alignment of the highly conserved USP10-uORF locus in three species[1].

(D) Conservation of ORF structures based on alignment of the 221 sORFs$_{3\text{-}15aa}$.

(E) Boxplots with PhyloCSF scores calculated across all aligned primate (left) and mammalian (right) genomes, for all sORFs$_{3\text{-}15aa}$ and a subset of sORFs$_{3\text{-}15aa}$ related to protein translation (see also **Figures 6A-D** and **Figures S6A-F**). The distribution of PhyloCSF scores for all sORFs$_{3\text{-}15aa}$ is not

statistically different compared to the subset of translational sORFs$_{3\text{-}15aa}$ (Wilcoxon one-sided signed rank test p-value$_{primates}$ = 0.77 and p-value$_{mammals}$ = 0.32).

(F) Prediction of MHC presentation of the peptides$_{3\text{-}15aa}$ translated from sORFs$_{3\text{-}15aa}$ by MHCpan-4.1[4].

(G) Fraction of uORFs (n = 3,286, including novel sORFs$_{3\text{-}15aa}$, blue) and lncORFs (n = 2,225, including novel sORFs$_{3\text{-}15aa}$, yellow) with conserved structures per length interval, for five selected mammalian species. For comparison, non-translated random ORF sequences were sampled from the same transcript regions (5' UTRs or lncRNAs, dashed line). See also **STAR Methods**.

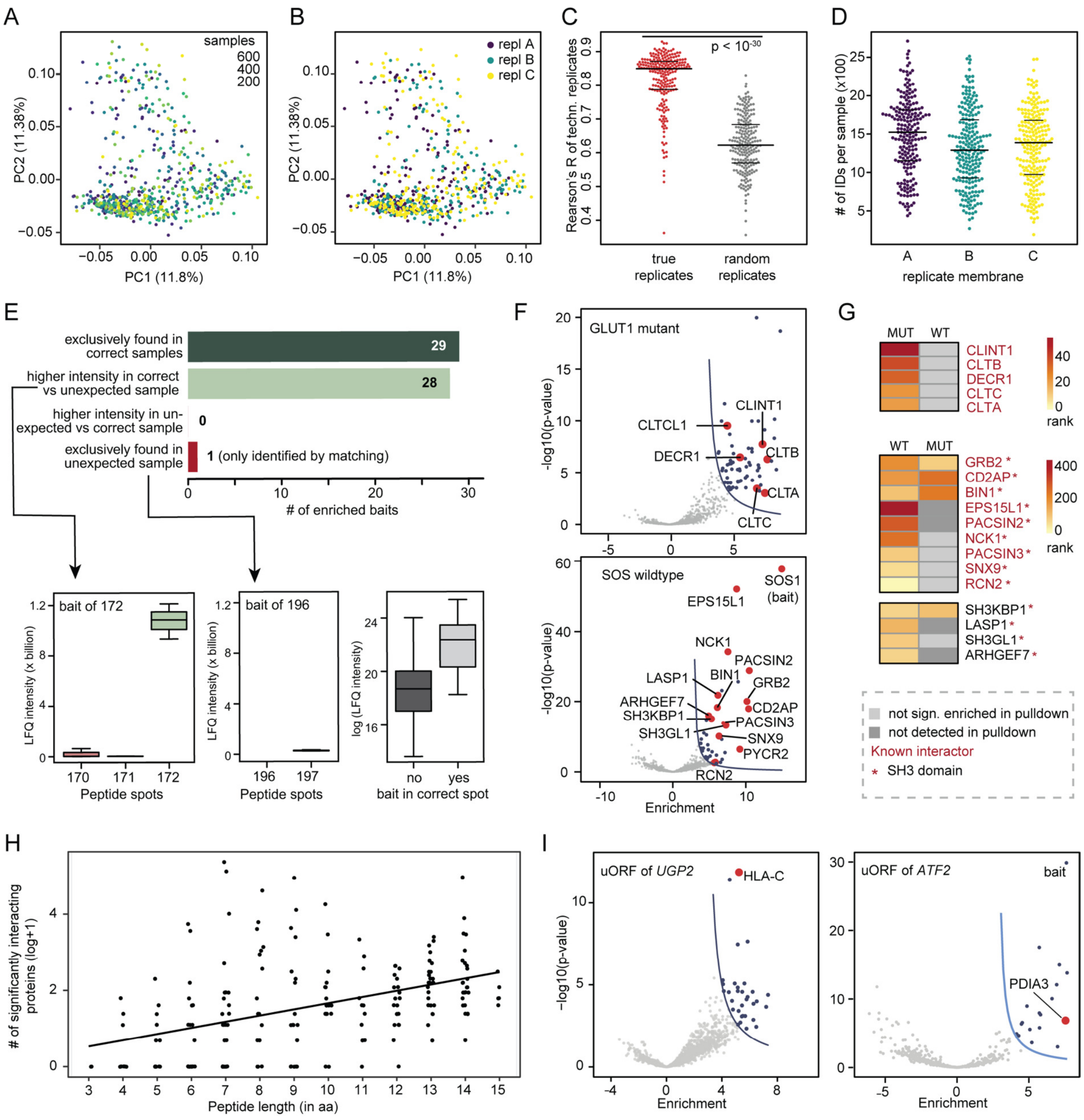

Figure S5. Peptides encoded by sORFs$_{3\text{-}15aa}$ have distinct interaction profiles, Related to Figure 5.
(A) PCA analysis of all individual samples of all replicates (colored by number) analyzed by PRISMA showing no bias.

(B) PCA analysis of the three replicates per PRISMA sample (colored by replicate) showing no bias.

(C) Pearson's correlation of true PRISMA replicates is significantly higher than correlations of random samples as assessed by Welch's t-test. The horizontal lines indicate 25, 50 and 75% quartiles, respectively.

(D) Distribution of total numbers of identified proteins per replicate. The horizontal lines indicate 25, 50 and 75% quartiles, respectively.

(E) Analysis of bait identification (**STAR Methods**).

(F) Volcano plots of GLUT1 mutant (top) and SOS1 wild type (bottom) with known interactors highlighted. All five known interactors of GLUT1 mutant were detected in the mutant and not in the wild type (see also **Figure S5G**). All nine known interactors of SOS1 wild type and four novel interactors with SH3-domain were detected in the wild type and mostly not in the mutant (see also **Figure S5G**).

(G) Top: all five known interactors of GLUT1 mutant, colored by their interaction ranks (calculated as product of fold change and p-value). As expected, the interactors are significantly binding to the mutant and not to the wild type. Bottom: all nine known interactors of SOS1 wild type and four novel interactors with SH3-domain, colored by their ranks. As expected, the interactors bind significantly to the wild type and mostly not to the mutant. An interactor that is not significantly enriched in a specific pulldown is depicted in light gray, an interactor that is not found at all in the respective pulldown is colored in dark gray.

(H) Number of significantly interacting proteins per peptide$_{3\text{-}15aa}$ by peptide length, baits were excluded.

(I) Volcano plots with the interactomes of the uORF-peptides$_{3\text{-}15aa}$ of *UGP2* (left) and *ATF2* (right). Highlighted are two proteins that are involved in MHC presentation. Both uORF-peptides are predicted to be presented on MHC *in silico* and ATF2-uORF was additionally detected in immunopeptidomics datasets (**Table S4**).

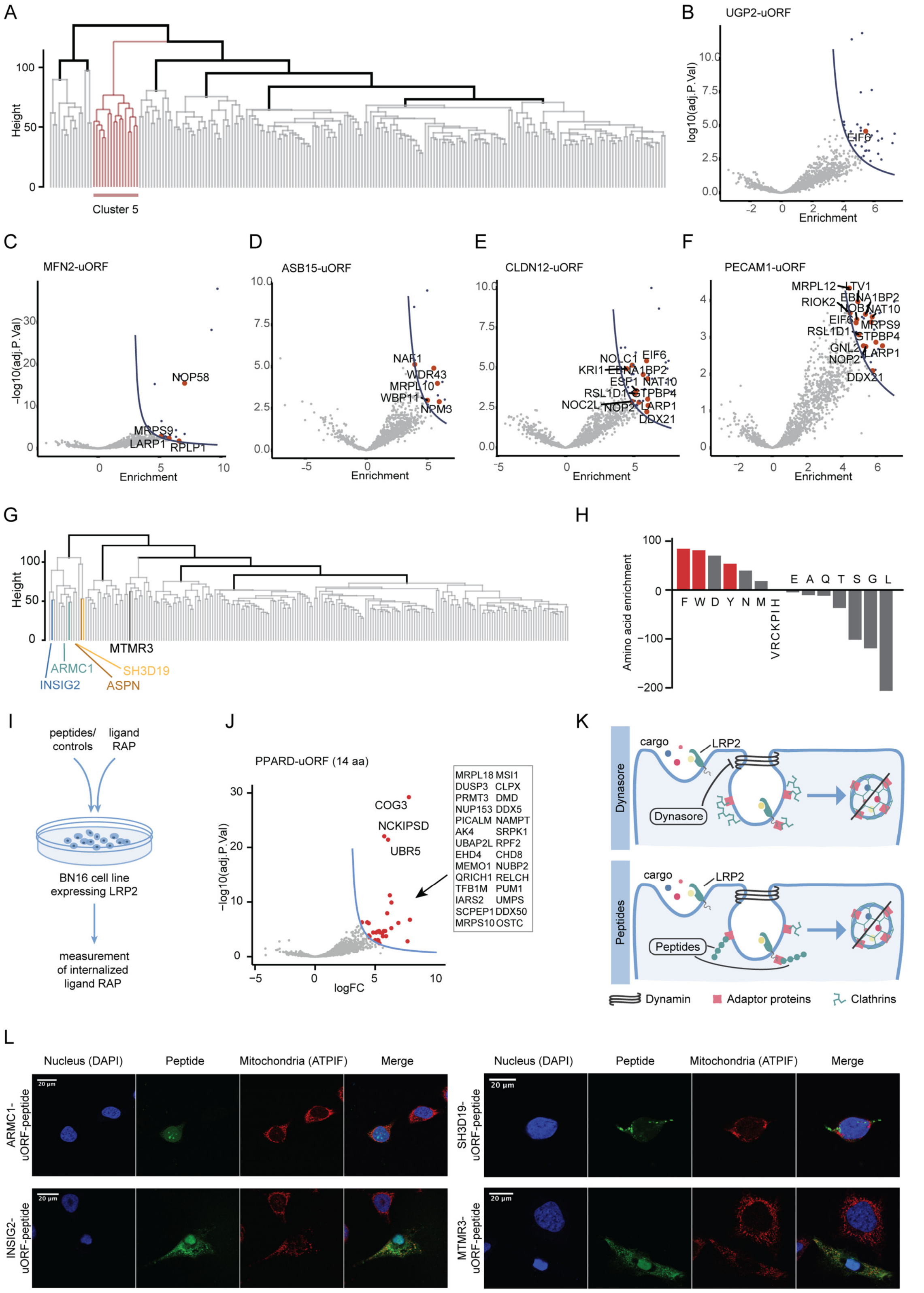

Figure S6. Peptide interactomes can predict modulators of cellular function, Related to Figure 6.
(A) Cluster of ribosome-binding peptides$_{3\text{-}15aa}$ highlighted in the global clustering overview.

(B-F) Volcano plots presenting interactomes of the five peptides$_{3\text{-}15aa}$ used in the luciferase assay. Interacting proteins highlighted in red are associated with the GO terms *translation* (GO:0006412) and *ribosome assembly* (GO:0042255).

(G) Localization within the global cluster overview of the four AP-binding uORF-peptides$_{3\text{-}15aa}$ translated from *ASPN, SH3D19, INSIG2* and *ARMC1*, as well as the uORF-peptides$_{3\text{-}15aa}$ translated from *MTMR3* that interacted with clathrins.

(H) Relative amino acid composition of the four endocytosis-related uORF-peptides$_{3\text{-}15aa}$ of *ASPN*, *SH3D19*, *ARMC1* and *INSIG2*. The enrichment is calculated by the formula: 100 - (% aa in the four endocytosis-peptides*100 / % aa in all peptides). Highlighted in red are aromatic, hydrophobic amino acids.

(I) Schematic overview of the endocytosis assay in BN16 cells. We selected the three uORF-peptides$_{3\text{-}15aa}$ of *INSIG2*, *ARMC1* and *SH3D19* for the assay, as the uORF-peptides$_{3\text{-}15aa}$ of *ASPN* also bound several proteins related to RNA-binding and splicing (**Figure 6E and Table S4**). For the assay, we measured the uptake of a physiological ligand (receptor-associated protein, RAP) by the potent endocytic receptor low-density lipoprotein (LDL) receptor-related protein 2 (LRP2/megalin) in Brown Norway rat choriocarcinoma (BN16) cells[5] in the presence or absence of peptide treatment.

(J) Volcano plot of the uORF-peptide$_{3\text{-}15aa}$ of the gene *PPARD* that does not bind any adaptor proteins and was used as a negative control in the endocytosis assay (**Figure 6H and 6I**).

(K) Top: schematic of how Dynasore inhibits dynamin and thereby endocytic vesicle formation. Bottom: possible mechanism for peptide-mediated inhibition of endocytosis.

(L) Immunofluorescence images of the synthetic, fluorescently labelled peptides$_{3\text{-}15aa}$ used in the endocytosis assay (**Figure 6H and 6I**). Additionally shown is the uORF-peptide$_{3\text{-}15aa}$ of *MTMR3* (**Figure 5C–5E**). All peptides have a TAT-sequence attached at the N-terminus and enter the cell. Cell nuclei were stained with DAPI and mitochondria with antibodies against ATPIF1.

Supplemental Items

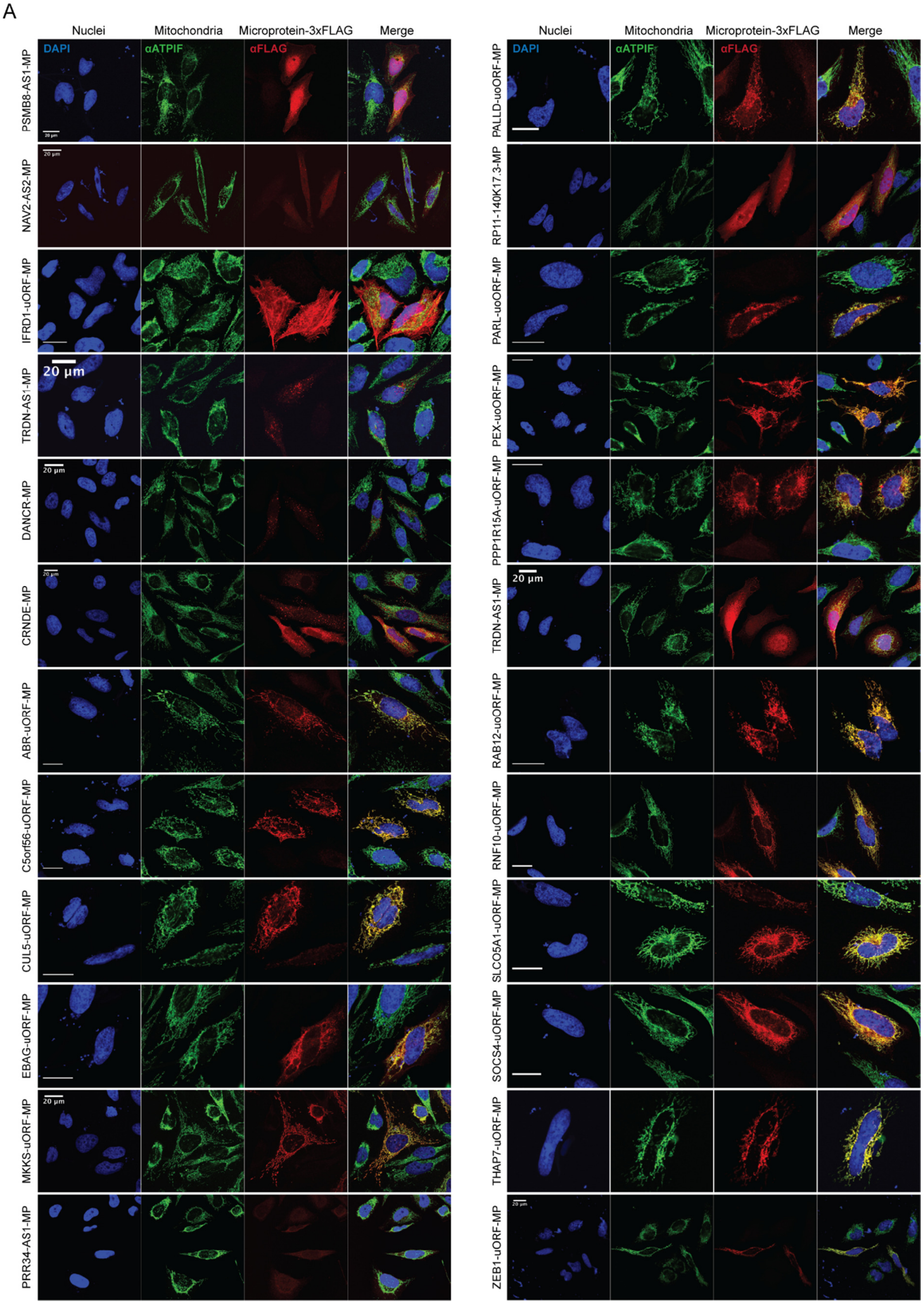

Data S1. Detection of overexpressed microproteins using immunofluorescence (IF) stainings, related to Figure 2.

(A) Twenty-six out of 31 FLAG-tagged microproteins were detected upon overexpression in HeLa cells using IF stainings. Twenty-four of the 26 are displayed above. PVT1-MP and LINC01128-MP are displayed in **Figure 3F and I; Figure S3E.** Cell nuclei were stained with DAPI, mitochondria with anti-ATPIF antibody and microproteins with anti-FLAG antibody. Scale bars represent 20 µm

Supplemental References

1. Wang, Z.-Y., Leushkin, E., Liechti, A., Ovchinnikova, S., Mößinger, K., Brüning, T., Rummel, C., Grützner, F., Cardoso-Moreira, M., Janich, P., et al. (2020). Transcriptome and translatome co-evolution in mammals. Nature *588*, 642–647.

2. Ruiz-Orera, J., Hernandez-Rodriguez, J., Chiva, C., Sabidó, E., Kondova, I., Bontrop, R., Marqués-Bonet, T., and Albà, M.M. (2015). Origins of De Novo Genes in Human and Chimpanzee. PLoS Genet. *11*, e1005721.

3. Clauwaert, J., McVey, Z., Gupta, R., and Menschaert, G. TIS Transformer: Re-annotation of the human proteome using deep learning. 10.1101/2021.11.18.468957.

4. Reynisson, B., Alvarez, B., Paul, S., Peters, B., and Nielsen, M. (2020). NetMHCpan-4.1 and NetMHCIIpan-4.0: improved predictions of MHC antigen presentation by concurrent motif deconvolution and integration of MS MHC eluted ligand data. Nucleic Acids Res. *48*, W449–W454.

5. Christensen, E.I., and Birn, H. (2002). Megalin and cubilin: multifunctional endocytic receptors. Nat. Rev. Mol. Cell Biol. *3*, 256–266.
